# Genome mining and functional characterization of four type I sesquiterpene synthases from the Tiger Milk Mushroom *Lignosus rhinocerus*

**DOI:** 10.1007/s13659-025-00578-9

**Published:** 2026-02-03

**Authors:** Ming-Xuan Gao, Li-Li Guo, Meng-Ting Wang, Xin-Yi Zhang, Xinyang Li, Shin-Yee Fung, He-Ping Chen, Ji-Kai Liu

**Affiliations:** 1 International Cooperation Base for Active Substances in Traditional Chinese Medicine in Hubei Province, School of Pharmaceutical Sciences, South-Central Minzu University, Wuhan, China; 2 Medicinal Mushroom Research Group (MMRG), Mycological Sciences Laboratory, Department of Molecular Medicine, Faculty of Medicine, Universiti Malaya, 50603 Kuala Lumpur, Malaysia

**Keywords:** *Lignosus rhinocerus* (Cooke) Ryvarden, Tiger Milk Mushroom, Sesquiterpene synthase, Heterologous expression

## Abstract

The Tiger Milk mushrooms (*Lignosus* spp.) have long been used as traditional folk medicines throughout Southeast Asia. However, the chemical constituents of these species remain largely unexplored. In this study, four type I sesquiterpene synthases (LrhTS1−LrhTS4) from *Lignosus rhinocerus* TM02^®^ were functionally characterized through genome mining, in vitro enzymatic assays, and heterologous expression in an *Escherichia coli* host engineered to overexpress an exogenous mevalonate pathway. The enzyme products were analyzed by GC−MS and further purified and structurally elucidated by NMR spectroscopy. A total of 11 sesquiterpenes (**1**−**11**) were identified, among which two were unstable and converted to stable derivatives (**2** to **2a** and **7** to **7a**). Notably, compounds **1**−**10** are reported from the genus *Lignosus* for the first time. LrhTS1 and LrhTS3 exhibited high catalytic specificity, whereas LrhTS2 and LrhTS4 displayed product promiscuity. This work expands the knowledge of terpene synthase from mushroom, and advances the understanding of the chemical basis underlying the bioactivity of the *Lignosus rhinocerus* TM02®.

**Graphical Abstract:**

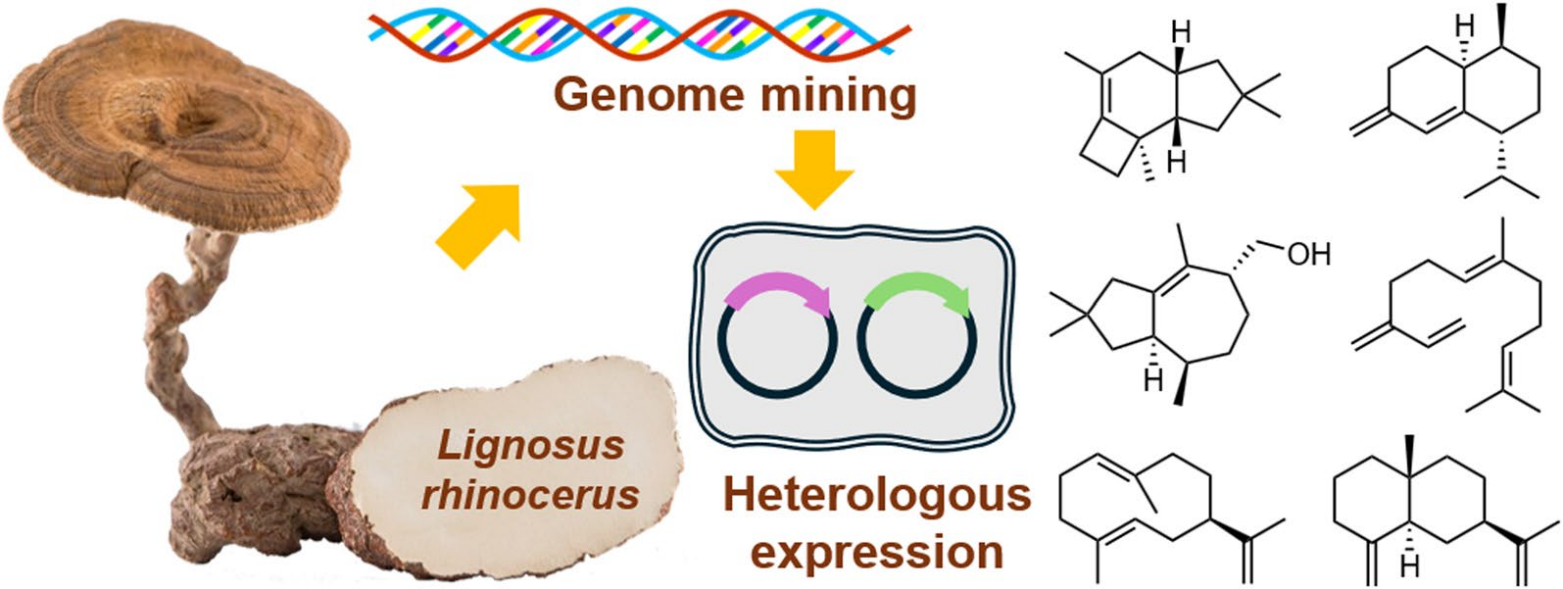

**Supplementary Information:**

The online version contains supplementary material available at 10.1007/s13659-025-00578-9.

## Introduction

Basidiomycetous mushrooms, a distinct subgroup of fungi featuring fruiting bodies-forming, are particularly significant as sources of bioactive secondary metabolites, including terpenoids, alkaloids, and polyketides [1]. Exploration of the medicinal and pharmacological potential of these underexplored mushroom species offers both scientific and biotechnological benefits [2–4]. Tiger Milk mushrooms (*Lignosus* spp.), which belong to the Polyporaceae family, are rare medicinal fungi found only in tropical rainforests across regions such as Southern China, Thailand, Malaysia, Indonesia, Philippines, and Papua New Guinea [5]. Recognized as a national treasure of Malaysia, three *Lignosus* species have been reported there, the *L. rhinocerus*, *L. tigris*, and *L. cameronensis* [6, 7]. These mushrooms are valued in traditional medicine for their healthy and therapeutic properties, including the treatment of ailments such as cancer, asthma, and bronchitis, as well as for their role in enhancing immunity [6]. However, research on the secondary metabolite profiles of *Lignosus* spp. has lagged far behind their widespread medicinal usage, only a few volatile components, amino acids, and polysaccharides, and specialized metabolites have been reported. Given this, the specialized metabolites of *Lignosus* spp. are worthy of further investigation. Unfortunately, the natural population of Tiger Milk mushrooms is extremely limited, unable to meet the growing market demand in recent years. The genomic sequence of *L. rhinocerus* TM02^®^ was reported in 2014 [8] with a genome size of 34.3 Mb and encoding 10,742 putative genes, which offers an opportunity for investigating its natural product profiles by genome mining.

Genome mining, facilitated by heterologous expression in genetically modified microbial hosts, is a proven technique for exploring the chemical potential of genes encoded in basidiomycetous genomes that are difficult to obtain under natural or standard laboratory conditions [9]. Genome annotation of *L. rhinocerus* TM02^®^ revealed that it contains numerous genes involved in the production of specialized metabolites, including polyketides, nonribosomal peptides, and terpenoids [6, 10–12]. Previous studies on three sesquiterpene synthase genes (*GME3634*, *GME3638*, *GME9210*) from *L. rhinocerus* by heterologous expression in *Saccharomyces cerevisiae* led to the identification of several sesquiterpenes, including the two cadinanes, the (+)-torreyol and *α*-cadinol, which were characterized by NMR [13]. Other sesquiterpenes were identified by GC–MS analysis, with their EI-MS fragmentation peaks compared to those in NIST (National Institute of Standards and Technology) Mass Spectral Libraries database, a widely used reference database for identifying chemical compounds based on EI-MS data [14]. In this study, we revisited the genome of *L. rhinocerus* TM02^®^, from which revealed a considerable number of uncharacterized terpene synthase genes. Here, we report the mining of terpene synthase genes from *L. rhinoceros* TM02^®^ through bioinformatical analysis, along with the characterization of their enzymatic products via in vitro and in vivo experiments using *Escherichia coli* as a host.

## Results and discussion

### Construction of an artificial pathway to overproduce DMAPP and IPP precursors in* Escherichia coli*

**Fig. 1:**
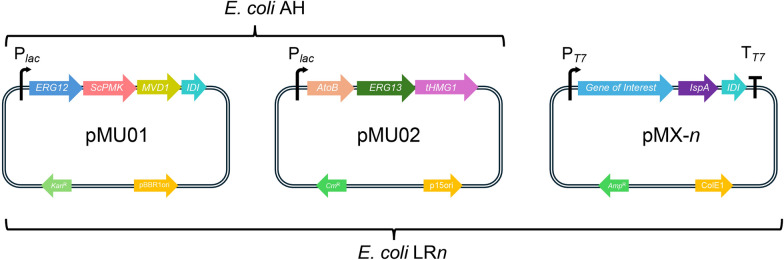
The plasmids and *E. coli* hosts used in this study

The widely used and effective strategy for enhancing the production of dimethylallyl pyrophosphate (DMAPP) and isopentenyl pyrophosphate (IPP) in *Escherichia coli* involves the overexpression of combinatorial mevalonate pathway genes from various sources. Therefore, prior to characterizing the terpene synthases, an engineered *E. coli* strain with elevated terpene precursor levels was constructed (Fig. 1). Following the work of Zhu et al. [15], the first plasmid containing the genes *atoB* (acetoacetyl-CoA thiolase), *erg13* (3-hydroxy-3-methylglutaryl-CoA synthase), *thmg1* (a truncated version of 3-hydroxy-3-methylglutaryl-CoA reductase) were constructed and named pMU02. The second plasmid containing the genes *erg12* (mevalonate kinase), *mvd1* (mevalonate pyrophosphate decarboxylase), *erg8* (codon optimized, phosphomevalonate kinase) and *idi* (isopentenyl pyrophosphate isomerase) were assembled to obtain pMU01. Notably, the genes in each plasmid were transcribed as a single polycistron, driven by the *lac* promoter. Both pMU01 and pMU02 were individually and sequentially transformed into *E. coli* BL21(DE3) to obtain the strain *E. coli* AH. The dual-plasmid system, which harbors the essential genes of the mevalonate pathway, was confirmed to work effectively in *E. coli* BL21(DE3) in our laboratory (data not shown).

### Genetic characterization of terpene synthases from *Lignosus rhinocerus*

The genomic sequence of the mushroom *Lignosus rhinoceros* TM02^®^ has been published [8]. The genomic data was downloaded and analyzed bioinformatically using Basic Local Alignment Search Tool (BLAST) with the amino acid sequences of ApLS [16], Copu9 [17], and AcCop4 [18], which were identified from the mushrooms *Agrocybe pediades*, *Coniophora puteana*, and *Antrodia cinnamomea*, respectively. The analysis revealed 13 genes in the *L. rhinoceros* genome that are putatively involved in encoding sesquiterpene synthases. These DNA sequences were further analyzed by phylogenetic analysis with the reported sesquiterpene synthases (Figure S1). Four genes were selected for further study: *LrhTS1*-*LrhTS4*. The introns of these genes have been revised by analysis with the published similar sequences from Protein Data Bank. The amino acid sequences of these genes all contain the typical and conserved domains of class I terpene synthase: the aspartate-rich DDXXD motif, the NSE triad, the highly conserved Arg residues [19], which play a role as pyrophosphate sensor and are typically located 46 residues upstream of the NSE triad, and the highly conserved C-terminal WXXXXXRY motif [16]. These features suggest that these enzymes are likely Mg^2+^-dependent, as they include binding sites for Mg^2+^. Beyond the conserved motifs mentioned above, *LrhTS1-4* share up to 75.23% sequence similarity with ApLS, Copu9, and AcCop4.

### In vitro characterization of LrhTS1-LrhTS4

To further investigate the function of the putative genes, the coding regions of *LrhTS1*-*LrhTS4* were synthesized and cloned into the pET-22b expression vector. The resulting plasmids were then expressed in *Escherichia coli* BL21(DE3). The four sesquiterpene synthases *LrhTS1*-LrhTS4 were expressed in soluble forms with a C-terminally tagged hexahistidine fusion protein, and purified by Ni^2+^ affinity chromatography. Sodium dodecyl sulfate polyacrylamide gel electrophoresis (SDS-PAGE) analysis confirmed that the sizes of the proteins matched the predicted molecular weights (Figure S2).

**Fig. 2:**
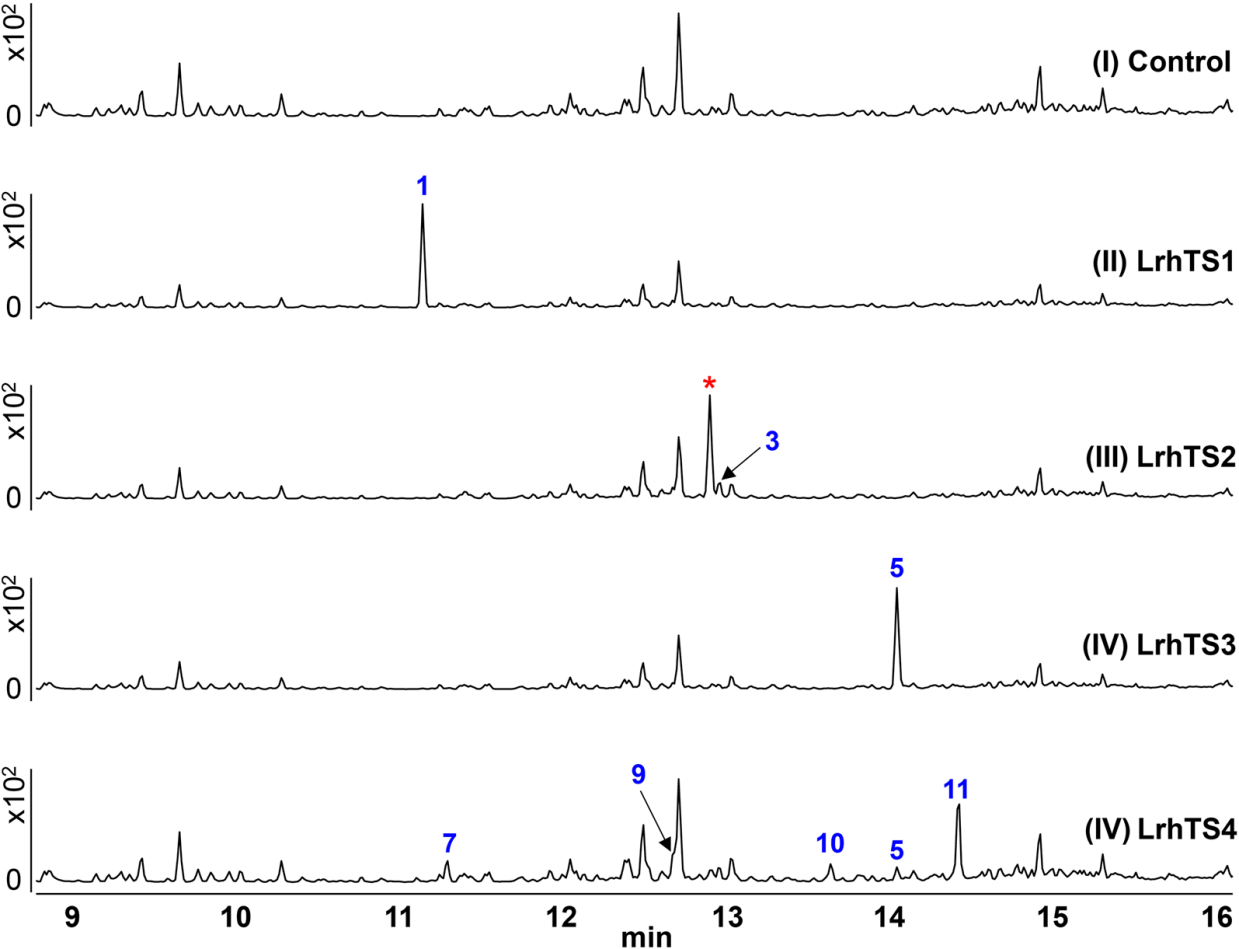
The GC–MS profile of the in vitro assays of the control group (I), and LrhTS1-LrhTS4 (I–IV, Blue: characterized by NMR; Red: not been isolated)

With these soluble proteins prepared, in vitro assays were carried out with farnesyl pyrophosphate (FPP) as substrates. The *n*-hexane layers of the in vitro assays were analyzed by gas chromatography coupled with electron impact mass spectrometry (GC–EI-MS) (Fig. 2). The Mg^2+^-omitted groups showed no additional peaks compared to the negative control (Figure S3-S6), confirming that all the four proteins were Mg^2+^-dependent terpene synthases. Besides, the GC–MS results also suggested that these enzymes only accepted FPP as the substrate.

The GC–MS data of the in vitro screening of LrhTS1 and LrhTS3 gave only one prominent product, with the molecular ion peak at *m/z* 204 [M]^+^ and 222 [M]^+^, respectively. In contrast, LrhTS2 and LrhTS4 exhibited low selectivity, generating multiple products. Notably, the main cyclization products of all four terpene synthases displayed different retention times and fragmentation patterns, but there are also several compounds with similar properties. The fragment ion patterns of the new products were compared with the NIST (National Institute of Standards and Technology) database, respectively (Figure S7–S18).

### Characterization of LrhTS1-LrhTS4 by heterologous expression in* E. coli*

To elucidate the structures of the enzymatic products, a synthetic biology approach was employed to produce sufficient quantities of the products for spectroscopic analysis. By using the *E. coli* AH as the chassis, each terpene synthase gene-containing pET22b plasmids (pET22b-*LrhTS1*-*LrhTS4*) was modified by introducing two additional genes, one is the farnesyl diphosphate synthase (*ispA*) gene (GenBank MBC0950805.1), the other is isopentenyl pyrophosphate isomerase (*idi*) gene (GenBank MBB0920478.1), resulting in plasmid pMX1-4 (Fig. 1), respectively. Both genes were amplified from the *E. coli* genome, with a ribosomal binding site sequence (5′-AAGGAG-3′) added before the start codon. These genes, along with the terpene synthase genes, were transcribed as a polycistron unit with the order *TS-ispA-idi* under the control of the T7 promoter. The plasmids pMX1-4 were individually transformed into the *E. coli* AH chassis and screened by ternary antibiotics (ampicillin, kanamycin, and chloramphenicol) to obtain *E. coli* strain LR1-4 (Fig. 1). After shaking fermentation of the resulting *E. coli* strain in terrific broth (TB) for three days, aliquots of the cultures were partitioned against *n*-hexane. The cultures were then harvested and partitioned against ethyl acetate, followed by concentration in vacuum to obtain crude extracts. Flash column chromatography eluting with petroleum ether were first employed on the crude extracts to remove background materials and enrich the terpenoid fractions. Semi-preparative high-performance liquid chromatography was then applied to further purify the products.

**Fig. 3:**
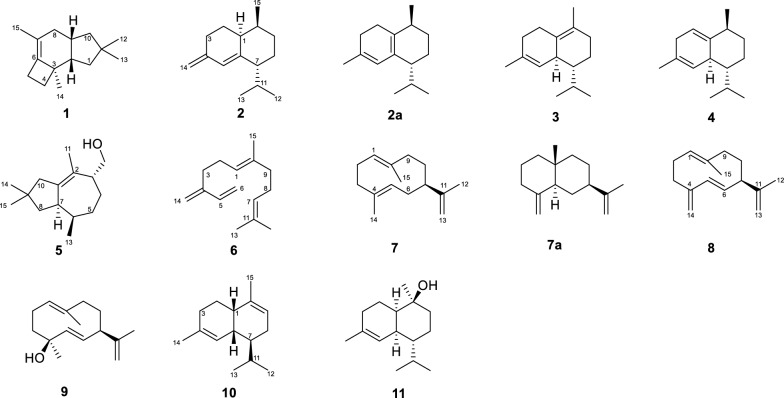
Chemical structures of isolated compounds

Compound **1** (Fig. 3) was isolated as a colorless oil from *E. coli* LR1 (containing *Lrh*TS1). The retention time and fragmentation pattern of **1** in GC–MS analysis was same as the peak in the in vitro assay of LrhTS1 (Figure S3), suggesting that compound **1** was stable during the isolation procedures. The ^13^C NMR data revealed 15 carbon signals. Notably, the carbons were free of oxygen attached according to the chemical shifts. These data, in combination with the molecular weight of 204 Da as deduced from GC–MS, suggested that **1** was tricyclic and possessed a double bond to satisfy the unsaturation requirements. These data were identical with those of previously described sesquiterpene Δ^6^-protoilludene [20]. Compound **1** was thus determined. Notably, when using the EI-MS fragment ion peaks of compound **1** as a query to search the NIST database, none of the returned results suggested that compound **1** was a protoilludane sesquiterpene (Figure S7). The best match result was 1,3,4,5,6,7-hexahydro-2,5,5-trimethyl-2*H*-2,4a-ethanonaphthalene, an acid-treated rearrangement product of thujopsene [21, 22]. Although the EI-MS fragmentation patterns of the two compounds were nearly identical, however, their ^1^H NMR spectroscopic data displayed distinct differences.

Compounds **2**, **3**, and **4** are all catalytic products of LrhTS2 (Fig. 3), which produces sesquiterpenes in a bicyclic system by catalyzing the substrate FPP. Meanwhile, *Lrh*TS4 also generate a small amount of compound **3** and a major compound (marked with asterisk in Fig. 2). A variety of isolation methods were attempted, but none yielded pure products.

Compound **2**, a colorless oil, was isolated from the cultures of *E. coli* LR2 (containing LrhTS2). Interestingly, the GC–MS analysis of **2** showed that it was absent from the in vitro assays of LrhTS2 (Figure S4). The ^13^C NMR spectrum of **2** exhibited fifteen carbon resonances, which were identical to those of naphthene [23], a cadinene-type sesquiterpene isolated from the soft coral *Nephthea* sp. Nephthene has only been reported with ^13^C NMR data, and lacks ^1^H NMR data as well as information on its absolute configuration. In this study, the ^1^H NMR data of naphthene were reported (Supplementary Material), which were assigned through interpretation of the HSQC spectrum. The EI-MS data of compound **2** was the same as the best match record in NIST database (Figure S8). Therefore, the absolute configuration of **2** was determined.

Notably, compound **2** was unstable when dissolved in deuterated chloroform for NMR spectra measurements and eventually transformed into compound **2a** after a few days of storage [24]. Compound **2a** was purified and identified by NMR. Comparison of the ^1^H NMR data (Supplementary Material) with those of obtained from compound **2** suggested that the protons at *δ*_H_ 4.68 (1H, s, H-14a) and *δ*_H_ 4.63 (1H, s, H-14b) disappeared and an additional methyl group (*δ*_H_ 1.79, 3H, s, H-14) appeared instead. The NMR spectroscopy analysis revealed that it was an isomerization product of compound **2**, which contains a diene moiety. Isomerization of compound **2** to compound **2a** involves conversion of an exocyclic double bond to a thermodynamically more stable endocyclic double bond.

Compound **3** (Fig. 3) was isolated as a colorless oil from the cultures of *E. coli* LR2 (containing LrhTS2). The retention time and fragmentation pattern of compound **3** in GC–MS analysis was same as the peak in in vitro assay of LrhTS2 (Figure S4). The ^1^H NMR spectrum of compound **3** revealed a series of singlets, corresponding to four methyl moieties, indicating that the compound is a sesquiterpene. The data highly resembled those of (−)-*δ*-cadinene, a sesquiterpene isolated from *Streptomyces clavuligerus* by genome mining [25]. It is noteworthy that the best hit of compound **3** in NIST database is also a cadinene sesquiterpene, which differs in the position of double bond (Figure S9).

Compound **4** (Fig. 3) was isolated as a colorless oil from the cultures of *E. coli* LR2 (containing LrhTS2). This compound was not detected in GC–MS analysis of the in vitro assay of LrhTS2 (Figure S4). The ^1^H and ^13^C NMR spectroscopic data of compound **4** were similar with those of the sesquiterpene (+)-cubenene [26]. However, the chemical shifts of C-13, and C-14 of (+)-cubenene in the original report (C-13: *d*_C_ 18.5; C-14: 21.8) had been assigned erroneously after carefully examined the assignment data. By comparison with the NMR data of compounds **2**–**3**, the correct assignments for C-13 and C-14 are 21.8, and 18.5 ppm, respectively. The EI-MS data of compound **4** was identical to the best hit in NIST database (Figure S10).

Compound **5** (Fig. 3) was isolated as a colorless oil from *E. coli* LR3 (containing LrhTS3). The retention time and fragmentation mode of compound **5** in GC–MS analysis are same as the peak in the in vitro assay of LrhTS3 (Figure S5). The in vitro and in vivo studies suggested that LrhTS3 showed high specificity as only one product (compound **5**) was detected. The ^1^H spectrum of compound **5** exhibited characteristic signals for four methyl groups and one oxygen-attach methylene group. The data indicated that compound **5** is a bicyclic sesquiterpene, which is highly consistent with the reported iltremulanol A [27], a tremulane-type sesquiterpene produced by the terpene synthase ILIS, which was characterized from the higher fungus *Irpex lacteus*. Interestingly, despite a relatively low percent identity of only 27.22% between LrhTS3 and ILIS, both enzymes produce the same sesquiterpene compound. Notably, compound **5** was included in NIST prediction (Figure S11).

The catalytic selectivity of LrhTS4 is low, and compounds **5**–**11** are all the enzymatic products of LrhTS4 (Figure S6). Among them, compounds **6** and **8** were not detected in in vitro assays of LrhTS4, these two compounds may be by-products of heterologous expression. It is also worth noting that compound **5** has been detected in both LrhTS3 and LrhTS4 catalytic reaction.

Compound **6** (Fig. 3) was isolated as a colorless oil from *E. coli* LR4 (containing LrhTS4). When detecting the in vitro assay of LrhTS4, this compound was not detected, which may be a by-product of heterologous expression. The 1D NMR spectroscopic analysis clearly showed eight double bond carbons at *d*_C_ 131.5 (s, C-2), 124.2 (d, C-3), 135.6 (s, C-6), 124.5 (d, C-7), 146.3 (s, C-12), 139.1 (d, C-13), 113.2 (t, C-14), 115.9 (t, C-15), five methylenes, and two methyls. These data were highly consistent with the those of (*E*)-*β*-farnesene [28], and this structure is consistent with the prediction of the NIST database (Figure S12).

Compound **7** (Fig. 3), a colorless oil, was isolated from the cultures of *E. coli* LR4 (containing LrhTS4). It was detected in in vitro enzymatic reaction of LrhTS4. The ^13^C NMR data of compound **7** clearly showed signals to three double bonds, *δ*_C_ 126.5 (d, C-1), 129.1 (s, C-4), 131.8 (d, C-5), 138.3 (s, C-10), 153.9 (s, C-11), 107.4 (t, C-13), as well as three methyls. The data of this compound is quite similar to that of a known compound germacrene A [29], which is deca-membered ring sesquiterpene. According to previous studies [30], this compound is prone to rearrangement reactions, resulting in a bicyclic system. In this study, when compound **7** was stored in deuterated chloroform for NMR spectroscopic analysis, it converted to compound **7a** several days later. Compared to the reported NMR data, it was determined that compound **7a** is *β*-selinene [31]. Notably, compound **7** was not included in NIST prediction (Figure S13).

Compound **8** was isolated as a colorless oil from *E. coli* LR4 (containing LrhTS4). This compound was absent in in vitro enzyme reaction of LrhTS4. The ^13^C NMR chemical shifts displayed fifteen carbon resonances, including eight olefinic carbons of which three were non-protonated, three were mono-protonated, and two bi-protonated. These data highly resemble to those of germacrene D [32], a decacyclic sesquiterpene isolated from cubebe pepper (*Piper cubeba*) [33]. This structure is consistent with the predictions of the NIST database (Figure S14). However, the absolute configuration of the compound has not been determined previously. Since compounds **6**–**11** were all enzymatic products of LrhTS4, which implied that the absolute configuration of compound **8** was consistent with compound **7**. Therefore, compound **8** was elucidated as (7*R*)-germacrene D.

Compound **9** was isolated as colorless oil from the cultures of *E. coli* LR4 (containing LrhTS4). The retention time and fragmentation pattern of the isolated compound **9** in GC–MS analysis was same as the peak detected in in vitro assay of LrhTS4. The ^13^C NMR spectrum of compound **9** showed the presence of six double bond carbons, while the ^1^H NMR spectrum displayed three methyl singlets, three olefinic protons, including two of which were assigned to a *trans* double bond by the large coupling constants (*J* = 15.7 Hz). By comparing to the reported NMR data, it is determined that the compound **9** is germacra-1(10),5-dien-4*β-*ol [34]. This result is also consistent with the prediction of the NIST database (Figure S15).

Compound **10** was isolated as a colorless oil. This compound was detected in in vitro enzymatic reaction of LrhTS4. The ^13^C NMR and DEPT revealed that compound **10** was a cadinene-type sesquiterpene, and the data was same as those of *α*-murolene [35]. Therefore, compound **10** was elucidated. The EI-MS data of compound **10** was same as the best-matched record in NIST database (Figure S16).

Compound **11** was isolated as a colorless oil from the cultures of *E. coli* LR4 (containing LrhTS4). This compound was detected in in vitro assay of LrhTS4. The ^13^C NMR data of compound **11** was same as the reported sesquiterpene (–)-torreyol [36], a sesquiterpene isolated from the plant *Pilgerodendron uvifera*, and ( +)-torreyol, isolated from the fungus *Stereum frustulosum* (Pers.) Fr [37]. The specific optical rotation of compound **11** was measured, the result ([α] + 50.8, *c* 0.05, CHCl_3_) suggested that compound **11** was ( +)-torreyol. Consequently, compound **11** was identified This structure is consistent with the predictions of the NIST database (Figure S17).

## Conclusion

*Lignosus rhinoceros* (Tiger Milk Mushroom) is a traditionally used medicinal fungus of considerable pharmacological importance. Elucidating the chemical basis of its bioactivity is essential for understanding its therapeutic potential. However, due to the scarcity of wild resources, progress in this area has been limited. In this work, genome mining, in vitro enzymatic assays, and heterologous expression were employed to functionally characterize terpene synthases from *L. rhinocerotis*. Four type I terpene synthases (LrhTS1–LrhTS4) were investigated, leading to the identification of 11 sesquiterpenoid metabolites. LrhTS1 and LrhTS3 exhibited high catalytic specificity, each affording a single product, whereas LrhTS2 and LrhTS4 showed broader substrate promiscuity. Structural elucidation revealed diverse carbon skeletons, including protoilludane, cadinane, tremulane, and germacrane sleketons. Compounds **1**–**10** are reported from *L. rhinoceros* for the first time. During structural determination, compounds **1**, **5**, and **7** escaped predictions from NIST database, while other compounds were identified by NIST prediction and NMR spectroscopic analysis.

Although the genus *Lignosus* comprises multiple species, the absence of publicly available genomic and transcriptomic data for other members restricted this investigation to a single species. Overall, this work expands current knowledge of the sesquiterpenoid diversity in *L. rhinoceros* and provides a foundation for future studies on its biosynthesis and potential pharmaceutical applications.

## Experimental

### Engineering of* Escherichia coli* with recombinant mevalonate pathway

Firstly, the change of Origin of Replication (*ori*) sequence of the plasmid pBBRMCS1 was finished by following steps. Fragment I was amplified from the plasmid pACYCDuet-1 by the primer pair p15A_F/R. The plasmid pBBRMCS1 was linearized by PCR with the primers pBBR1_15A_F/R to obtain fragment II. Then fragments I and II were ligated by In-Fusion Cloning to obtain the plasmid pBBRMCS1-p15A (chloramphenicol resistance). Secondly, the truncated *hmg1* (*thmg1*) gene fragment was amplified from the genomic DNA of *Saccharomyces cerevisiae* INVSc1 (primers pET28a_tHMG1_F/R), and it was inserted into the EcoRI and HindIII-linearized pET28a to obtain the plasmid pET28a_tHMG1. After confirmation of the soluble expression of tHMG1 in *E. coli* BL21(DE3) used in this study, the fragment of *thmg1* was amplified from pET28a_tHMG1 by the primers tHMG_28a_F/tHMG_pBBR1_R to give fragment III. The *atoB* was amplified from the genomic DNA of *E. coli* ATCC25922 by the primers AtoB_F/R to give fragment IV, the *erg13* was amplified from the gDNA of *S. cerevisiae* INVSc1 by the primers Erg13_F/R to afford fragment V. Fragments III-V were inserted into the SmaI-digested pBBRMCS1-p15A by Gibson cloning to obtain the plasmid pMU02. Thirdly, the codon-optimized sequence of *ScPMK* (designated as *ScPMKop* in this study) was synthesized and ligated into the plasmid pET28a to give pET28a_ScPMKop. After confirmation of the soluble expression of ScPMKop in *E. coli* BL21(DE3) used in this study, the ScPMKop was amplified by the primers ScPMK_F/R to obtain fragment VI. *EcIDI* was amplified from the gDNA of *E. coli* ATCC25922 (primers pET28a_EcIDI_F/R) and ligated into the NdeI-HindIII-digested pET28a, and further been amplified by the primers EcIDI_28a_F/R to give fragment VII. The *erg12*, *mvd1* were each amplified from the gDNA of *S. cerevisiae* INVSc1 by the primers ERG12_F/R and MVD1_F/R, respectively, and were inserted into the PCR-linearized pBBRMCS2 (primers pBBRMCS2_F/R) together with fragment VI and VII to obtain the plasmid pMU01 (ampicillin resistance). The plasmids pMU02 and pMU01 were introduced into *E. coli* BL21(DE3). One positive clone was selected and been made as competent cells for further use. Primes used here were listed in Table S1.

### Bioinformatics analysis

The genome sequence of *Lignosus rhinocerus* TM02® was downloaded from NCBI (GCA_000743315.1). The genomic coding sequences were annotated by AUGUSTUS (the reference species used was *Phanerochaete chrysosporium* and *Aspergillus fumigatus*). The genome sequence was subjected to online antiSMASH analysis (fungal version) by using Cluster Blast and Cluster Pfam analysis. The *Agrocybe pediades* linalool synthase ApLS was used as query sequence to blast against the annotated protein sequences of *L. rhinocerus* TM02^®^ by local BlastP program. The resulted amino acid sequences were subjected to phylogenetic analysis with the reported terpene synthases. Multiple amino acid sequences alignment were performed using MAFFT program [38]. The phylogenetic tree was built by the FastTree program and visualized by the web-based TVBOT application [39]. Eight postulated terpene synthase were selected for further study. The introns of the selected coding sequences were revised by blast against the RefSeq Selected protein and PDB recorded sequence databases. The revised coding sequences of *LrhTS1*-*LrhTS4* were ordered from the Universe Gene Technology (Tianjin) Co., Ltd without codon optimization. The synthetic genes were inserted into the pET22b vector linearized by NdeI and HindIII. The DNA sequences of the terpene synthase gene were listed in Table S2.

### Protein expression and purification

The recombinant plasmids were transformed into *E. coli* BL21(DE3) for protein expression. A single colony of *E. coli* harboring the corresponding plasmids were inoculated into 15 mL centrifuge tubes contained 4 mL LB medium supplemented with 100 mg/L ampicillin and cultivated overnight at 37 °C, 180 rpm. The seeding cultures were used to innoculate the 400 mL fresh LB cultures supplemented with 100 mg/L ampicillin, which were grew at 37 °C until the OD_600_ reached 0.6–0.8. After the cultures were submerged in ice water for 30 min, protein expression was induced with 300 mM of isopropyl thiogalactoside (IPTG) at 16 °C, 180 rpm for 20 h.

The protein purification protocols were attached in Supplementary Information.

### In vitro enzymatic assays

In vitro enzymatic reactions were performed in which it was expressed in *E. coli*. Standard enzyme assays (200 μL) were performed in Tris buffer (50 mM, pH 7.5) containing farnesyl pyrophosphate FPP (0.1 mM), Mg^2+^ (2 mM), and purified protein (10 μM) at 30 °C for overnight. Four sets of control experiments were set up, including groups as follows: (a) without enzyme, (b) with boiled enzyme, (c) without substrate, and (d) without Mg^2+^. The overnight reactions were quenched by adding 200 μL of *n*-hexane. The organic layers were subjected to GC–MS analysis.

### In vivo heterologous expression

The two plasmids pMU01 and pMU02 harboring the necessary genes of the mevalonate pathway were constructed as described in the literature. The two plasmids were transformed sequentially into *E. coli* BL21(DE3) host to obtain the chasis cell *E. coli* FM with a high titre of DMAPP and IPP.

The farnesyl diphosphate synthase gene *IspA* (GenBank: CAQ30890.1) and the isopentenyl-diphosphate delta-isomerase gene *Idi* (GenBank: WP_001192790.1) were amplified from the genomic sample of *E. coli* BL21(DE3) with the primers listed in Table S1. The amplified fragments of *IspA* and *Idi* with the corresponding overhangs were introduced into the linearized of pET22b-*LrhTS1-4* plasmids to generate the plasmids pMX-1–4, respectively. Among those, pET22b-*LrhTS3* was linearized using NotI, while the other four plasmids were linearized using HindIII. Plasmid DNA were isolated from the positive clones grown on LB-ampicillin by using the AxyPrep Plasmid Miniprep Kit and further verifed by Sanger sequencing.

The plasmids pMX1-4 was each transformed into *E. coli* FM, and the resulting strains were grown in 200 mL TB media (containing 20 g/L peptone, 24 g/L yeast extracts, 4 mL glycerol, 12.54 g of K_2_HPO_4_, 2.31 g of KH_2_PO_4_) supplied with 100 mg/L ampicillin, 50 mg/L kanamycin, and 34 mg/L chloramphenicol in 500 mL flasks. At an OD_600_ of 0.6−0.8, protein expression were induced by adding 300 μM IPTG for 20 h at 16 °C. The cultures were fermented for an additional 72 h at 28 °C, followed by extraction with ethyl acetate.

### Compounds isolation

The culture of the recombinant *E. coli* LR1 (containing pMX1) strain was grown in TB media. After a 72-h growth, the culture was extracted using ethyl acetate. The extracts were subsequently dried under vacuum and the residues were subjected to a silica gel column eluted with petroleum ether to yield compound **1**.

*E. coli* LR2 (containing pMX2) was cultivated in TB medium using the similar steps. The extracts were then subjected to a silica gel column eluted with petroleum ether − EtOAc (from v/v 100:0 to 20:80) to yield four fractions (fractions 1−4). Fraction 1 was further purified by HPLC (CH_3_CN 100%, 4 mL/min) to obtain compounds **2–4** (t_R_ = 15.016 min, t_R_ = 15.602 min, t_R_ = 17.444 min). Fraction 2 was further purified by HPLC (CH_3_CN/H_2_O from v/v 80:20 to 96:4, 20 min, 4 mL/min) to obtain subfraction 2–1 (t_R_ = 13.625 min). GC–MS detected compounds with ion fragmentation patterns identical to those of the major compound (marked with asterisk symbols in Fig. 2) in secondary fraction 2–1. However, none of the separation methods used produces a product that was pure.

The culture of *E. coli* LR3 (containing pMX3) was also extracted with ethyl acetate and separated by column chromatography over silica gel eluted with petroleum ether−EtOAc (from v/v 100:0 to 25:75) to afford compound **5**.

*E. coli* LR4 (containing pMX4) was cultured in TB medium for 72 h, generating 1 L culture broth. The solution was extracted thrice by EtOAc to obtain extract. Six fractions (fractions 1–6) and compounds **10** and **5** (the same as the product of pMX3) were obtained by subjecting the resultant extract to CC over silica gel eluted with petroleum ether − EtOAc (from 100:0 to 20:80). Fraction 1 was isolated by prep-HPLC (CH_3_CN, 100%, 4 mL/min) to obtain compounds** 6–9** (t_R_ = 10.970 min, t_R_ = 12.132 min, t_R_ = 13.158 min, t_R_ = 16.099 min). Fraction 4 was purified on Sephadex LH-20 (MeOH) to obtain compound **11**.

## Supplementary Information

Supplementary Material 1: The NMR chemical shift assignments and spectra, GC-EI-MS data, primers, DNA sequences, phylogenetic tree, and SDS-PAGE analysis of the purified proteins.

## Data Availability

The datasets generated during and/or analyzed during the current study are available from the corresponding author on reasonable request.
