# Supplementary material for: Genome mining and functional characterization of four type I sesquiterpene synthases from the Tiger Milk Mushroom Lignosus rhinocerus
Source: Nat Prod Bioprospect. 2026 Feb 3;16(1):27. doi: 10.1007/s13659-025-00578-9 (PMC12864622; doi:10.1007/s13659-025-00578-9)
Supplement: Supplementary file 1 — Supplementary Material 1: The NMR chemical shift assignments and spectra, GC-EI-MS data, primers, DNA sequences, phylogenetic tree, and SDS-PAGE analysis of the purified proteins. [file 13659_2025_578_MOESM1_ESM.pdf]

Supplementary Information for

Genome Mining and Functional Characterization of Four Type I Sesquiterpene Synthases from the Tiger Milk Mushroom *Lignosus rhinocerus*

Ming-Xuan Gao[a], Li-Li Guo[a], Meng-Ting Wang[a], Xin-Yi Zhang[a], Xinyang Li[a], Shin-Yee Fung[b], He-Ping Chen[a,*], Ji-Kai Liu[a,*]

[a]International Cooperation Base for Active Substances in Traditional Chinese Medicine in Hubei Province, School of Pharmaceutical Sciences, South-Central Minzu University

[b]Medicinal Mushroom Research Group (MMRG), Mycological Sciences Laboratory, Department of Molecular Medicine, Faculty of Medicine, Universiti Malaya, 50603 Kuala Lumpur, Malaysia

*Corresponding authors:

chenhp@mail.scuec.edu.cn (H.-P. Chen)

liujikai@mail.scuec.edu.cn (J.-K. Liu)

Contents

1. Experimental

2. The chemical shift assignments of the isolated compounds

3. Supplementary Tables

4. Supplementary Figures

1. Experimental

1.1 General

Optical rotations were obtained on an Autopol IV-T digital polarimeter (Rudolph, Hackettstown, USA). CD spectra were measured on a Chirascan Circular Dichroism Spectrometer (Applied Photophysics Limited, Leatherhead, Surrey, UK). 1D and 2D NMR spectra were obtained on a Bruker Avance III 600 MHz spectrometer (Bruker Corporation, Karlsruhe, Germany). Structural assignments were made with additional information from gHSQC experiments. Chemical shifts were given in δ (ppm) with solvent peaks as references ($CDCl_3$, δ_H 7.26, δ_C 77.16). All GC-MS analyses were performed on an Agilent 8890 GC with a 5977C MSD instrument with a HP-5MS column (30 m, 250 μm i.d., 0.25 μm film, Agilent Technologies). A flow rate of 1 mL min-1 of helium was used as carrier gas and electronic impact was 70 eV. Oven temperature was programmed from 50°C to 190°C in 14 min, and then to 250°C in 8 min, and hold at 250°C for further 5 min. Preparative high performance liquid chromatography (prep-HPLC) was performed on an Agilent 1260 Infinity II liquid chromatography system equipped with a YMC Triart C18 column (particle size 5 *μ*m, dimension 4.6 mm i.d. × 250 mm, flow rate 4 mL·min^{-1}) and a DAD detector (Agilent Technologies, Santa Clara, CA, USA). Silica gel (200-300 mesh, Qingdao Haiyang Chemical Co., Ltd., China) and Sephadex LH-20 (GE Healthcare, Sweden) were used for column chromatography (CC). Silica gel GF_{254} plates (Qingdao Haiyang Chemical Co., Ltd., China) was used for thin layer chromatography (TLC). The spots on TLC were visualized by immersing the plate into vanillin-sulfuric acid-ethanol, and then heating.

Oligonucleotide primers (Table S1) and *E. coli* TOP10 were ordered from Beijing Tsingke Biotech Co., Ltd (Wuhan Branch). *E. coli* BL21(DE3) (Novagen) used for protein expression and chassis cell was purchased from Sigma-Aldrich (No. 69450). AxyPrep Plasmid Miniprep Kit (AP-MN-P-250G) was used for plasmid extraction. Restriction enzymes Hind III and NotI were purchased from Takara Bio Inc. The PrimeSTAR® Max DNA Polymerase (Cat. No. R045A), used for PCR was purchased from Takara Bio Inc.

PCR reactions were performed using a Bio-Rad C1000 Touch™ Thermal Cycler. ClonExpress Ultra One Step Cloning Kit V2 (Cat. No. C116) used for cloning was purchased from Vazyme Biotech Co., Ltd (Nanjing, China). Sanger sequencing was performed by Beijing Tsingke Biotech Co., Ltd (Wuhan Branch). The LB medium used for protein expression (containing 5 g/L yeast extract, 10 g/L tryptone, 10 g/NaCl). The TB medium used for terpene production (containing 20 g/L peptone, 24 g/L yeast extracts, 4 mL glycerol, 12.54 g of K_2HPO_4, 2.31 g of KH_2PO_4). gDNA of *S. cerevisiae* INVSc1 was purified by CTAB method.

1.2 Protein purifications

The *E. coli* cells were collected by centrifugation at 6000 g, 4°C for 20 min, and resuspended in lysis buffer (50 mM Tris-HCl, pH 7.5, 200 mM NaCl, 5 % glycerol) with lysozyme (Biofroxx) and lysed by sonication on ice. The lysate were centrifuged at 9000 rpm, 4°C for 20 min to remove the cellular debris. The supernatants were loaded on a Ni-NTA affinity column, which was then washed with 120 mL wash buffer (50 mM Tris-HCl, pH 7.5, 200 mM NaCl, 30 mM imidazole, 5% glycerol). His_6-tagged proteins were eluted from the column by using 15 mL elute buffer (50 mM Tris-HCl, pH 7.5, 200 mM NaCl, 300 mM imidazole, 5% glycerol). The protein solution was ultrafiltrated and concentrated using 30 kDa Amicon Ultra-15 Centrifugal Filter (Millipore) at 4°C, 4000 rpm with imidazole-free buffer (50 mM Tris-HCl, pH 7.5, 200 mM NaCl, 5% glycerol). The purity of each purified protein was examined by sodium dodecyl sulfate-polyacrylamide gel electrophoresis (SDS-PAGE) (Figure S2). The protein concentrations were determined by the A_{280} value which were measured by a NanoDrop One spectrophotometer (Thermo Scientific). The purified proteins were flash frozend in liquid nitrogen and stored at −80°C for subsequent enzyme assay *in vitro*.

2. The chemical shift assignments of the isolated compounds.

Δ^6-protoilludene (1): $C_{15}H_{24}$, colorless oil. ^{1}H NMR (600 MHz, $CDCl_3$) δ_H 1.34 (1H, ddd, *J* = 12.5, 8.0, 1.8 Hz, H-1a), 1.29 (1H, m, H-1b), 2.16 (1H, m, H-2), 1.80 (1H, m, H-4a), 1.74 (1H, m, H-4b), 2.72, (1H, m, H-5a), 2.53 (1H, m, H-5b), 1.85 (1H, m, H-8a), 1.65 (1H, m, H-8b), 2.34 (1H, m, H-9), 1.53 (1H, ddd, *J* = 12.2, 7.4, 1.8 Hz, H-10a), 0.97 (1H, dd, *J* = 12.2, 2.0 Hz, H-10b), 1.05 (3H, s, H-12), 1.04, (3H, s, H-13), 0.93 (3H, s, H-14), 1.57 (3H, dd, *J* = 1.0, 1.0 Hz, H-15); ^{13}C NMR (150 MHz, $CDCl_3$) δ_C 41.2 (t, C-1), 47.2 (d, C-2), 45.8 (s, C-3), 37.0 (t, C-4), 25.7 (t, C-5), 141.9 (s, C-6), 123.3 (s, C-7), 34.3 (t, C-8), 40.7 (d, C-9), 48.7 (t, C-10), 39.4 (s, C-11), 20.6 (q, C-12), 29.9 (q, C-13), 27.6 (q, C-14), 17.5 (q, C-15).

Nephthene (2): $C_{15}H_{24}$, colorless oil. ^{1}H NMR (600 MHz, $CDCl_3$) δ_H 1.95 (1H, m, H-1), 2.32 (1H, m, H-2a), 2.16 (1H, m, H-2b), 1.71 (1H, m, H-3a), 1.54 (1H, m, H-3b), 5.99 (1H, s, H-5), 1.69 (1H, m, H-7), 1.65 (2H, m, H-8), 1.82 (1H, m, H-9a), 1.32 (1H, m, H-9b), 2.36 (1H, m, H-10), 1.85 (1H, m, H-11), 0.92 (3H, d, *J* = 6.6 Hz, H-12), 0.75 (3H, d, *J* = 6.6 Hz, H-12), 4.68 (1H, s, H-14a), 4.63 (1H, s, H-14b), 0.87 (3H, d, *J* = 7.0 Hz, H-15); ^{13}C NMR (150 MHz, $CDCl_3$) δ_C 35.0 (d, C-1), 29.7 (t, C-2), 27.3 (t, C-3), 144.5 (s, C-4), 126.6 (d, C-5), 144.8 (s, C-6), 51.3 (d, C-7), 22.9 (t, C-8), 29.5 (t, C-9), 37.1 (d, C-10), 27.4 (d, C-11), 21.5 (q, C-12), 21.9 (q, C-13), 108.1 (t, C-14), 15.0 (q, C-15).

(–)-*Trans*-cadina-1(6),4-diene (2a): $C_{15}H_{24}$, colorless oil. ^{1}H NMR (600 MHz, $CDCl_3$) δ_H 1.91-2.20 (2H, ov, H-2), 1.91-2.20 (2H, ov, H-3), 5.61 (1H, s, H-5), 1.91-2.20 (1H, ov, H-7), 1.62 (1H, m, H-8a), 1.38/1.16 (1H, m, H-8b), 1.62 (1H, m, H-9a), 1.38/1.16 (1H, m, H-9b), 1.91-2.20 (1H, ov, H-10), 1.91-2.20 (1H, ov, H-11), 0.70 (3H, d, *J* = 6.6 Hz, H-12), 0.92 (3H, d, *J* = 6.6 Hz, H-13), 1.79 (3H, s, H-14), 0.96 (3H, d, *J* = 7.0 Hz, H-15); ^{13}C NMR (150 MHz, $CDCl_3$) δ_C 133.0 (s, C-1), 28.9 (t, C-2), 30.4 (t, C-3), 132.4 (s, C-4), 122.7 (d, C-5), 130.1 (s, C-6), 42.8 (d, C-7), 20.7 (t, C-8), 27.3 (t, C-9), 33.7 (d, C-10), 29.7 (d, C-11), 17.5 (q, C-12), 21.3 (q, C-13), 23.3 (q, C-14), 19.2 (q, C-15).

(+)-*δ*-Cadinene (3): $C_{15}H_{24}$, colorless oil. ^{1}H NMR (600 MHz, $CDCl_3$) δ_H 2.70 (1H, ddd, *J* = 12.8, 4.8, 2.4 Hz, H-2a), 2.03-1.86 (1H, ov, H-2b), 2.03-1.86 (2H, ov, H-3), 5.45 (1H, br s, H-5), 2.52 (1H, d, *J* = 9.8 Hz, H-6), 1.05 (1H, m, H-7), 1.61 (1H, m, H-8a), 1.16 (1H, m, H-8b), 2.03-1.86 (2H, ov, H-9), 2.05 (1H, m, H-11), 0.78 (3H, d, *J* = 6.9 Hz, H-12), 0.96 (3H, d, *J* = 6.9 Hz, H-13), 1.67 (3H, s, H-14), 1.65 (3H, s, H-15); ^{13}C NMR (150 MHz, $CDCl_3$) δ_C 130.1 (s, C-1), 26.9 (t, C-2), 32.1 (t, C-3), 134.4 (s, C-4), 124.8 (d, C-5), 39.6 (d, C-6), 45.5 (s, C-7), 21.3 (t, C-8), 32.5 (t, C-9), 124.7 (s, C-10), 26.8 (d, C-11), 15.8 (q, C-12), 21.9 (q, C-13), 23.7 (q, C-14), 18.7 (q, C-15).

(+)-Cubenene (4): $C_{15}H_{24}$, colorless oil. ^{1}H NMR (600 MHz, $CDCl_3$) δ_H 5.33 (1H, s, H-2), 2.58 (2H, m, H-3), 5.54 (1H, s, H-5), 2.38 (1H, m, H-6), 1.13 (1H, m, H-7), 1.66 (1H, m, H-8a), 0.96 (1H, m, H-8b), 1.84 (1H, m, H-9a), 1.22 (1H, m, H-9b), 1.91 (1H, m, H-10), 2.09 (1H, m, H-11), 0.88 (3H, d, *J* = 7.0 Hz, H-12), 0.84 (3H, d, *J* = 7.0 Hz, H-13), 1.70 (3H, m, H-14), 1.03 (3H, d, *J* = 6.5 Hz, H-15); ^{13}C NMR (150 MHz, $CDCl_3$) δ_C 143.3 (s, C-1), 112.7 (t, C-2), 36.9 (t, C-3), 131.5 (s, C-4), 121.3 (d, C-5), 51.3 (d, C-6), 37.6 (d, C-7), 24.7 (t, C-8), 31.6 (t, C-9), 42.0 (d, C-10), 26.7 (d, C-11), 15.2 (q, C-12), 21.6 (q, C-13), 23.5 (q, C-14), 18.3 (q, C-15).

Iltremulanol A (5): $C_{15}H_{26}O$, colorless oil. ^1H NMR (600 MHz, $CDCl_3$) δ_H 2.28 (1H, m, H-3), 1.79 (1H, m, H-4a), 1.69 (1H, m, H-4b), 1.88 (1H, m, H-5a), 1.56 (1H, m, H-5b), 1.73 (1H, m, H-6a), 22.94 (1H, m, H-7), 1.54 (1H, ddd, J = 12.2, 8.2, 2.3 Hz H-8), 1.84 (1H, m, H-10), 2.07 (1H, dd, J = 14.9, 2.2 Hz H-10), 1.69 (3H, t, J = 2.0 Hz, H-11), 3.77 (1H, dd, J = 10.7, 9.7 Hz, H-12a), 3.69 (1H, dd, J = 10.7, 6.2 Hz H-12b), 0.8 (3H, d, J = 6.9 Hz, H-13), 0.83 (3H, s, H-14), 1.05 (3H, s, H-15); ^{13}C NMR (150 MHz, $CDCl_3$) δ_C 139.9 (s, C-1), 127.7 (s, C-2), 48.8 (d, C-3), 20.7 (t, C-4), 32.0 (t, C-5), 31.9 (d, C-6), 46.0 (s, C-7), 45.9 (t, C-8), 37.0 (s, C-9), 49.0 (t, C-10), 23.8 (q, C-11), 61.4 (t, C-12), 12.2 (q, C-13), 27.2 (q, C-14), 28.8 (q, C-15).

E-*β*-farnesene (6): $C_{15}H_{24}$, colorless oil. ^1H NMR (600 MHz, $CDCl_3$) δ_H 1.68 (3H, s, H-1), 5.16 (2H, ddd, J = 5.9, 5.8,1.2 Hz, H-3), 2.06 (2H, m, H-4), 1.99 (2H, m, H-5), 5.10 (1H, ddd, J = 5.9, 5.8,1.2 Hz, H-7), 2.19 (2H, m, H-8), 1.60 (3H, s, H-9), 1.60 (3H, s, H-10), 2.22 (2H, m, H-11), 6.38 (1H, dd, J = 17.6, 10.8 Hz, H-13), 5.25 (1H, d, J = 17.6 Hz, H-14a), 5.06 (1H, d, J = 10.8 Hz, H-14b), 5.01 (2H, d, J = 10.8 Hz, H-15); ^{13}C NMR (150 MHz, $CDCl_3$) δ_C 25.8 (q, C-1), 131.5 (s, C-2), 124.2 (d, C-3), 26.7 (t, C-4), 39.8 (t, C-5), 135.6 (s, C-6), 124.5 (d, C-7), 26.8 (t, C-8), 17.8 (q, C-9), 16.2 (q, C-10), 31.5 (t, C-11), 146.3 (s, C-12), 139.1 (d, C-13), 113.2 (t, C-14), 115.9 (t, C-15).

Germacrene A (7): $C_{15}H_{24}$, colorless oil. ^1H NMR (600 MHz, $CDCl_3$) δ_H 4.91 (1H, dd, J = 11.8, 2.8 Hz, H-1), 2.94-1.93 (2H, ov, H-2), 2.94-1.93 (2H, ov, H-3), 4.67 (1H, br d, J = 10.0 Hz, H-5), 2.94-1.93 (2H, ov, H-6), 2.94-1.93 (1H, ov, H-7), 2.94-1.93 (2H, ov, H-8), 2.94-1.93 (2H, ov, H-9), 1.71 (3H, s, H-12), 4.80 (1H, br s, H-13a), 4.70 (1H, br s, H-13b), 1.53 (3H, s, H-14), 1.66 (3H, s, H-15); ^{13}C NMR (150 MHz, $CDCl_3$) δ_C 126.5 (d, C-1), 26.8 (t, C-2), 39.7 (t, C-3), 129.1 (s, C-4), 131.8 (d, C-5), 35.0 (t, C-6), 51.5 (d, C-7), 33.8 (t, C-8), 41.8 (t, C-9), 138.3 (s, C-10), 153.9 (s, C-11), 20.4 (q, C-12), 108.2 (t, C-13), 16.4 (q, C-14), 16.8 (q, C-15).

β-Selinene (7a): $C_{15}H_{24}$, colorless oil. ^1H NMR (600 MHz, $CDCl_3$) δ_H 1.43 (1H, ov, H-1a), 1.27 (1H, ov, H-1b), 1.61 (2H, m, H-2), 2.30 (1H, br d, J = 13.4 Hz, H-3a), 2.00 (1H, m, H-3b), 1.82 (1H, br d, J = 12.4 Hz, H-5), 1.55 (1H, ov, H-6a), 1.29 (1H, ov, H-6b), 1.96 (1H, m, H-7), 1.55 (2H, ov, H-8), 1.49 (1H, m, H-9a), 1.28 (1H, m, H-9b), 4.71 (2H, d, J = 7.8 Hz, H-12), 1.75 (3H, s, H-13), 4.69 (1H, ov, H-14a), 4.43 (1H, br d, J = 1.6 Hz, H-14b), 0.72 (3H, s, H-15); ^{13}C NMR (150 MHz, $CDCl_3$) δ_C 42.1 (t, C-1), 23.6 (t, C-2), 37.0 (t, C-3), 151.1 (s, C-4), 50.0 (d, C-5), 29.8 (t, C-6), 46.0 (d, C-7), 26.9 (t, C-8), 41.3 (t, C-9), 36.1 (s, C-10), 151.2 (s, C-11), 22.8 (q, C-12), 108.3 (t, C-13), 105.5 (t, C-14), 16.5 (q, C-15).

Germacrene D (8): $C_{15}H_{24}$, colorless oil. ^1H NMR (600 MHz, $CDCl_3$) δ_H 5.13 (1H, dd, J = 11.5,4.8 Hz, H-1), 2.40 (1H, m, H-2a), 2.00 (2H, ov, H-2b), 2.43 (1H, m, H-3a), 2.10 (1H, m, H-3b), 5.78 (1H, d, J = 15.9 Hz, H-5), 5.25 (1H, dd, J = 15.9, 9.9 Hz, H-6), 2.00 (1H, ov, H-7), 1.43 (2H, ov, H-8), 2.36 (1H, m, H-9a), 2.23 (1H, m, H-9b), 1.43 (1H, ov, H-11), 0.86 (3H, d, J = 6.7 Hz, H-12), 0.81 (3H, d, J = 6.7 Hz, H-13), 4.79 (1H, d, J = 2.2 Hz, H-14a), 4.74 (1H, d, J = 2.2 Hz, H-14b), 1.51 (3H, s, H-15); ^{13}C NMR (150 MHz, $CDCl_3$) δ_C 129.8 (d, C-1), 29.4 (t, C-2), 34.6 (t, C-3), 149.0 (s, C-4), 135.6 (d, C-5), 133.7 (d, C-6), 53.0 (d, C-7), 26.6 (t, C-8), 40.8 (t, C-9), 134.2 (s, C-10), 32.9 (d, C-11), 19.5 (q, C-12), 20.9 (q, C-13), 109.2 (q, C-14), 16.1 (t, C-15).

Germacra-1(10),5-dien-4*β*-ol (9): $C_{15}H_{26}O$, colorless oil. ^1H NMR (600 MHz, $CDCl_3$) δ_H 4.95 (1H, br d, *J* = 11.5 Hz, H-1), 2.50 (1H, m, H-2a), 1.94 (1H, br d, *J* = 14.5 Hz, H-2b), 2.24 (3H, m, H-3), 5.25 (1H, d, *J* = 15.7 Hz, H-5), 5.17 (1H, dd, *J* = 15.7, 9.6 Hz, H-6), 2.01 (1H, m, H-7), 1.63 (1H, m, H-8a), 1.54 (1H, ov, H-8b), 1.38 (2H, m, H-9), 1.42 (1H, m, H-11), 0.78 (3H, d, *J* = 6.8 Hz, H-12), 0.82 (3H, d, *J* = 6.8 Hz, H-13), 1.19 (3H, s, H-14), 1.54 (3H, br s, H-15); ^{13}C NMR (150 MHz, $CDCl_3$) δ_C 129.0 (d, C-1), 23.8 (t, C-2), 41.4 (t, C-3), 73.3 (d, C-4), 140.2 (d, C-5), 125.8 (d, C-6), 52.9 (d, C-7), 39.7 (t, C-8), 26.1 (t, C-9), 132.7 (s, C-10), 33.1 (d, C-11), 19.1 (q, C-12), 20.7 (q, C-13), 30.8 (q, C-14), 16.9 (q, C-15).

α-Muurolene (10): $C_{15}H_{24}$, colorless oil. ^1H NMR (600 MHz, $CDCl_3$) δ_H 1.80-2.01 (1H, ov, H-1), 1.76 (1H, m, H-2a), 1.42 (1H, ov, H-2b), 1.80-2.01 (2H, ov, H-3), 5.48 (1H, s, H-5), 2.05 (1H, m, H-6), 2.05 (1H, m, H-7), 1.80-2.01 (2H, ov, H-8), 5.41 (1H, s, H-9), 1.80-2.01 (1H, ov, H-11), 0.89 (3H, d, *J* = 6.9 Hz, H-12), 0.83 (3H, d, *J* = 6.9 Hz, H-13), 1.69 (3H, s, H-14), 1.69 (3H, s, H-15); ^{13}C NMR (150 MHz, $CDCl_3$) δ_C 39.2 (d, C-1), 24.8 (t, C-2), 30.6 (t, C-3), 134.6 (t, C-4), 124.3 (d, C-5), 36.8 (s, C-6), 41.1 (d, C-7), 24.6 (t, C-8), 121.6 (d, C-9), 136.6 (s, C-10), 26.7 (d, C-11), 15.9 (q, C-12), 21.5 (q, C-13), 24.1 (q, C-14), 21.8 (q, C-15).

(+)-Torreyol (11): $C_{15}H_{26}O$, colorless oil. ^1H NMR (600 MHz, $CDCl_3$) δ_H 1.59 (1H, m, H-1), 1.99 (2H, m, H-2), 1.49 (1H, m, H-3a), 1.09 (1H, m, H-3b), 5.52 (1H, d, *J* = 5.8 Hz, H-5), 2.01 (1H, m, H-6), 1.30 (1H, m, H-7), 1.88 (1H, m, H-8a), 1.56 (1H, m, H-8b), 1.55 (1H, m, H-9a), 1.51 (1H, m, H-9b), 1.97 (1H, m, H-11), 0.81 (3H, d, *J* = 7.0 Hz, H-12), 0.89 (3H, d, *J* = 7.0 Hz, H-13), 1.65 (3H, s, H-14), 1.29 (3H, s, H-15); ^{13}C NMR (150 MHz, $CDCl_3$) δ_C 45.7 (d, C-1), 31.3 (t, C-2), 21.6 (t, C-3), 134.5 (d, C-4), 124.7 (d, C-5), 36.9 (d, C-6), 44.2 (d, C-7), 18.6 (t, C-8), 35.5 (t, C-9), 72.7 (s, C-10), 26.5 (d, C-11), 15.4 (q, C-12), 21.8 (q, C-13), 23.8 (q, C-14), 28.1 (q, C-15).

3. Supplementary Tables

Table S1. Primers used in this study.

p15A_F	TGCCGCCGTGGGTTTCTCGAACGGGGCGGAGATTTCC
p15A_R	CCACTTTTTGCCGGAGCTCGAGAAATATTTTATCTGATTAA
pBBR1_15A_F	CTCCGGCAAAAAGTGGCCCC
pBBR1_15A_R	GAAACCCACGGCGGCAATGC
pET28a_tHMG1_F	GTCGCGGATCCGAATTCGTTTTAACCAATAAAACAGT
pET28a_tHMG1_R	AGTGCGGCCGCAAGCTTTAGGATTTAATGCAGGTGAC
tHMG1_F	GGAGGATTACACTATGGGCAGCAGCCATCATCA
tHMG1_R	TAGAACTAGTGGATCCTTAGGATTTAATGCAGGTGAC
AtoB_F	CGAATTCCTGCAGCCCGGGGATCCTCTAGAGTCGACTAGGAGGAATATAAAATGAAAAATTGTGTCATCGTC
AtoB_R	CATTTAGCTGTCCTCCTTAATTCAACCGTTCAATCACC
Erg13_F	GGAGGACAGCTAAATGAAACTCTCAACTAAACTTTG
Erg13_R	CATAGTGTAATCCTCCTTATTTTTTAACATCGTAAG
ScPMK_F	TTACCATGGACTTCATAAGAGGCAGATCAAATGTCAGAACTAAGGGCATTTAG
ScPMK_R	GAGTATTACCTCTTATTTGTCCAGGTACGTCTCC
pET28a_EcIDI_F	CGCGCGGCAGCCATATGCAAACGGAACACGTCATTT
pET28a_EcIDI_R	GTGCGGCCGCAAGCTTTATTTAAGCTGGGTAAATGC
EcIDI_28a_F	CTGCAGCCCGGGAGGAGGATTACTATATGGGCAGCAGCCATCATCATC
EcIDI_28a_R	GGGCGAATTGGAGCTCTTATTTAAGCTGGGTAAATGCAG
ERG12_F	TATCGAATTCCTGCAGTAGGAGGAATTAACCATGTCATTACCGTTCTTAACT
ERG12_R	TTATGAAGTCCATGGTAAATTCG
MVD1_F	ATAAGAGGTAATACTCATGACCGTTTACACAGCATCCG
MVD1_R	TCCTCCCGGGCTGCAGTTATTCCTTTGGTAGACCAGTC
LrhTS1_ispA_F	AGCCTGCGGTTGTGAAGCTTTAATTTAAGAAGGAGATATACCATGGACTTTCCGCAGCA
LrhTS2_ispA_F	AACAGCAGCAGTCAAAGCTTTAATTTAAGAAGGAGATATACCATGGACTTTCCGCAGCA
LrhTS3_ispA_F	CTTCATCGAAGCTTGCGGCCTAATTTAAGAAGGAGATATACCATGGACTTTCCGCAGCA
LrhTS4_ispA_F	TTGCTGCGGCGGCGAAGCTTTAATTTAAGAAGGAGATATACCATGGACTTTCCGCAGCA
LrhTS5_ispA_F	CGGATGATGAGGAGAAGCTTTAATTTAAGAAGGAGATATACCATGGACTTTCCGCAGCA
pMX1-seq1	GACGCATGGAGTTTCGAGAG
pMX2-seq1	ATGGAGCTTCAAGACCCAAC
pMX3-seq1	CAGGACTGGATGGTCGGTGC
pMX4-seq1	TCCCGAAGCGTCCGCAGAAC
ispA_seq1	TAGACGCTGAAGGCAAACAC
Idi_pET22b_HindIII_R	GTGCTCGAGTGCGGCCGCAAGCTTTTATTTAAGCTGGGTAAATG
Idi_pET22b_NotI_R	TGGTGGTGCTCGAGTGCGGCCGCTTATTTAAGCTGGGTAAATGCAG
pFZ81_seq_int3	CATGTCGTACCAAAGAGATTT
pMH1_seq_int3	GGTGCCTGTAAGATATGGTTAG
T7_promoter	TAATACGACTCACTATAGGG
T7_terminator	GCTAGTTATTGCTCAGCGGTG

Table S2. DNA sequences used in this study.

>*ScPMKop* (The optimized DNA sequence of *ScPMK*)

ATGTCAGAACTAAGGGCATTTAGTGCTCCCGGAAAAGCTTTGCTGGCGGGTGGCTATCTCGTTCTGGACACCAAATACGAGG
CGTTTGTTGTTGGTCTTTCTGCGCGTATGCATGCAGTTGCACATCCGTATGGCTCCCTGCAGGGTTCTGACAAATTTGAGGT
GCGTGTTAAGTCCAAGCAATTTAAGGACGGCGAATGGCTGTATCACATCAGCCCGAAGTCGGGCTTCATTCCGGTGTCGATA
GGCGGTAGCAAGAACCCGTTTATCGAAAAGGTGATCGCAAACGTTTTTAGCTATTTCAAACCAAATATGGATGACTACTGCAA
CCGCAACTTGTTTGTGATCGATATTTTCTCTGACGACGCCTACCACAGCCAAGAAGATAGCGTGACCGAGCACCGCGGTAAT
CGTCGCCTGTCGTTCCACAGCCACCGTATTGAGGAGGTGCCGAAAACCGGTCTGGGTTCGAGCGCGGGTCTGGTCACTGT
ACTCACAACCGCGTTAGCTAGCTTCTTCGTGAGCGACTTGGAGAACAACGTGGACAAGTACCGCGAAGTGATTCACAACCT
GGCGCAGGTTGCGCATTGTCAGGCGCAGGGTAAAATCGGTAGCGGCTTTGATGTTGCGGCTGCTGCGTATGGTAGCATTCG
TTATCGTCGTTTCCCGCCTGCACTGATTAGCAACCTGCCGGATATCGGTTCAGCTACCTACGGTTCCAAATTGGCGCACCTG
GTCGATGAAGAAGATTGGAATATTACCATTAAATCCAATCATTTACCGTCCGGCTTAACCCTGTGGATGGGTGATATTAAAAAC
GGCTCTGAAACGGTTAAATTGGTGCAAAAAGTTAAGAACTGGTATGACAGCCACATGCCGGAGTCGCTGAAGATCTATACCG
AGCTGGACCACGCCAACAGCCGTTTCATGGACGGCCTGAGTAAGCTGGATCGTCTGCATGAAACCCATGACGACTACAGCG
ACCAGATTTTCGAGAGCCTGGAACGTAATGATTGTACCTGCCAAAAATACCCGGAGATCACCGAAGTACGTGATGCAGTGGC
CACCATCCGCAGAAGCTTTCGTAAGATCACCAAAGAATCTGGTGCGGATATTGAACCGCCAGTTCAGACTTCATTGCTGGAC
GATTGCCAAACCTTGAAGGGTGTTCTTACGTGCCTGATTCCGGGTGCTGGCGGCTACGATGCGATCGCAGTCATCACCAAG
CAAGATGTGGACCTGCGTGCACAGACGGCCAATGATAAACGCTTCAGCAAGGTGCAATGGTTGGACGTGACCCAGGCGGA
CTGGGGTGTTCGCAAAGAGAAGGACCCGGAGACGTACCTGGACAAATAA

>LrTS1

ATGCCTTTGTCTTCGTCTGTTGTCGCTTTCCGCCTTCCTGACACGCTTGGGTGCTGGCCATGGCGACGCTGCCTGAACACG
CATTATGTGGAGGCCAAACAGGATTCGGCATCCTGGCTGGAGTCATTCCACCCGTTCGGCCCCAAGGCACAAAGGGCCTTC
AACAAGTGCGACTTCAACCTTCTCGCGTCCCTAGCGTATCCGGTGGCCAGCAAAGACCAACTGCGTGCAGGGTGTGACCT
GATGAACGTCTTCTTCGTCTTCGACGAGTACTCTGATGTCGAGAACGAGAAGACGGTCCAGCAGCTCGCGGACATCATCAT
GGACGCACTGCGAAACCCTCACAAGCCTCGTCCTGCAGGAGAATCTCTCGTGGGCGAAATCACTAGGCAGTTCTGGGCGC
GCACGATCAAGGTAGCGAGCGAACCGTCGCAGCGCCGATTCATCGAGACCTTTGACGACTACTGCCAGTCCGTAGTCCAAC
AGGCTGCGGACCGGTCGCAGAACCACTTGCGTGACGTCGAGAGCTACTTGGAGAACCGGAGGGAGAACATCGGCGCCAA
GCCGTCGTTTGCACTGCTCGAGTTGGACATGAACCTCCCCGACGAGGTGATCGAGCACCCGACCATCGTCAACCTGACGA
CATGGGCCATTGACATGATCATCCTGGGAAACGACATTGTCTCGTACAACGTGGAGCAGGCGCGTGGCGACGACGGACACA
ACGTCGTGACCATCGTCATGCATCACTACAACGTCGACGTGCAGGGTGCCATGGACCGCATCGCCGAGTGGCACCAGAGG
CTGGCGGACCAGTTCCTCACCAACTACAACAAGCTGCCGTCGTGGGGACGCGAGATTGACGCGCAAGTTGAGCGGTACAT
CCAGGGCATCGGAAACTGGGTACGCGCGAACGACGCATGGAGTTTCGAGAGCGAGAGGTACTTTGGATTGAACGGACGCG
AGATCGAGCAGAGTCGTTGGGTAACCCTACTCCCTCGGGTCTCCGCGGAGAAGCCTGCGGTTGTGTAA

>LrTS2

ATGGCCATCCAACAATTTATCCTCCCTGACCTGCTGGCGATGTGCCCTTTTGAGGGTTCAACAAGTCCACACTACGCCAAGG
CGTCAGCCGAATCCCGAGCATGGGTCAACAGCTACCACCTTTTTAAGGGAAGCAAGCTCGCCTTCTTTCTGCAGGGAAGCA
ACGAGCTTCTCGTCTCCCACACCTATCCCTACGCTGGATACGATCAGTTCAGGACCTGTTGCGACTTTGTCAATCTGCTCTTC
GTCGTGGACGAAGTCAGCGACGACCAGGACGGCAGAGGGGCCCGCAACACCGGCGAGGTGTTCCTGAACGCTATGCGCT
ACCCTGATTGGGATGATGGCTCTGCCCTGGCCAAGATGACTCGCGAGTTCAAACAACGTCTGCTGGAGCACGCCGGGCCC
GACTGCTACCGTCGATTTCTGATTCATTGCGCCGACTACGTTAACGCGGTTGCGCGAGAAGCCGAGCTCAGGGAACGGGG
CGAGGTCCTCGACACGGCGGACTTCCAGACATTACGTCGGGAGAACAGTGCTATTCGACTGTGCTTTGGCCTGTTCGAGTT
CGTGCTCGGTGTCGATTTGCCGGATGTCGTGTTCCAAGACGAGCATTTTATGACATTGTACTGGGCCGCGGCCGATATGGTT
TGCTGGTCTAACGATGTCTACTCGTACAACATGGAGCAAGCGAAGGGTCATACAGGGAACAACATCGTCACCGTTCTCATGT
ACGAGAAAAACATTGGTCTCCAGGCCGCGGCCGACCTCGTTGGCGAGCAGTTCCACCAGCTGATGAACCGATTTGTGGAG

ACTAAGCGACGCCTCCCGTCGTTCGCTGGGGGCGTTGACACCGCCGTCGCCAAGTATGTTGCCGCCATGGAGCATTGGGT
AATCGGGAACCTCGCATGGAGCTTCAAGACCCAACGCTACTTCGGCGCGGAGCATGGGCGGGTAAAAGAGACGCGAGTAG
TCGTGCTGCGCCCGCGCGAGCACGGGGACGATCAACAGCAGCAGTCATAA

>LrTS3
ATGCGCGCTCGATCGTTCATTCTTCCCGACCTCGTGTCGGATTGCCCATATACGCTCAGGTGCAACTCCAATTGCGAAGCTG
TTGCGCGCGCTTCAGAGGCCTGGATGCTCGAAGACGCGAATCTCTCACCGAAACGTCGTGACGCCTTCCTGCGTCTGCGG
GGGGGAGAACTCACGGCAGCGTGCTACCCCGATACGGACGAGGCCTGCCTGCGAGTCGCCGCGGACTTCCTCAATTTCCT
GTTTAGTTTGGACGACTGGTCGGACGAGTTCAGTATGGAGGACACTTGCGGGCTCGCGCAGTGCGTCATGTGTGTGCTCCA
TGATCCTGATGACTTCCAGACTGAAAAGGCTGCTGGCAAGCTCGCCAAAAGCTTTTTCAATCGGTTCCGGCAGACGGCGGG
GCCGAGGTGTACTCGTCGATTCATCGATAGTATGGACCTCTTCTTTCATGCGATTGCACAGCAAGCCCAGGACCGCGCGTCC
GGGTCTGCTCCCTCACTCGAAGAATATGTGGCCCTCCGCGAGGACACGAGCGGGTGCAAGCCCTGCTTCGCTCTCATCGA
ATACGCCGCGGGTATGGACCTTCCAGACCACGTCGCCCATCATCCAACAATCACCGCCCTCGAGCGGGAAGCCAACGCGT
GTATATCATGGTCGAACGATCTCTTCTCGTACAACGTCGAGCAAGCGCGGGGTGACACACACAACATGATTGCGGTGATCAT
GCGCGAGGACGGGCGCAGCTTGCAAGAAGCCGTTGAATACTTGGGCGCTCTCTGCAAGCTCTGCATAGTGCACTTCGAGG
AAAACCGAGCCATGCTGCCATCGTGGGGTCCGGAGATTGACGGGGAAGTCGACAGGTATGTGCTCGGCCTCCAGGACTGG
ATGGTCGGTGCCCTCCATTGGAGCTTCGACACCGCACGTTATTTCGGGGATGAAGGCCCTGCGATCAAGAAGCACGGTGTC
GTGACACTACTACCGCGGAAGTCTTCATCGTAA

>LrTS4
ATGTCTGCTGCCACTACTTTTGCCTCTGCGGTTGCGCCCAAGAAGTCTATCGTCGTCCAGACAGAGGACGGACCCACACAG
AGGTACCTGTACCTCCCGGATACGATGTCCAACTGGCCTTGGCCACGCATGATCAACCCGTACTACGAGGAGGTCACTCTC
GAGTCGAATGCGTGGTTCAAGTCCTTTAAGCCGTTCACTCCTGAGTCGCAGTATGCCTACGACAAATGTGATTTCGGCCGCC
TCGCATCCCTCGCCTACCCGGATATCTCACGGGAGGCGCTTCGAACCGGAATTGATTTGATGAACGTGTTCTTCGTCGTCGA
CGAATACACCGACGTCGAGCCTGCCCCCGTCGTCCGCGAGATGATCGAGATTGTTATTGACGCTCTGCACAACCCCGACAA
GCCCCGTCCTGAGGGCGAGATTCTCCTTGGCGAGATAACCAGACAGTTTTGGGCTCGTGGCCGCACAACGGCAACGCCCG
AGGCGGCGAAACACTTTGTTGAAGCGTTCACCGACTACCTGCGGTCGGTGATCTACCAGGCCGAGGACCGTGACAGCAAC
ACCGTCCGCACGATCGACAGCTACCTTGAAACGCGACGGGAGAACATTGGCGCCCGCCCCTCATACGTTCCGGGCGAGCT
GCATCTTTCCATCCCGGACGAGGCCTTCTACCACCCTGTCATCAAGGAACTCGAGTATCTTATCGCGGACCTTATCATCCTCG
ACAACGACATCGCGTCGTACAACAAAGAGCAGGCGACTGGCGACGATCGGCACAACATTCTCACGATCGCCATGCACCAGT
TCAACTGCGACTTCGACACCGCGATGGAGTGGGTCGTCAACTACCACAAGGACGTCGAGATGCGCTTCATAGACGGGCTCA
AGCGCGTGCCGTCCTGGGGCCCTAAGGTTGACGCGGAAGTGCAGGTGTACATCACGCACCTTGCCAACTGGCCACGTTGT
AACGACTGCTGGAACTTCGAGTCTGGGAGGTACTTTGGCAGCAAGGGCCTCGAGTACCAGAGGACCCGTCTCGTGCCGAT
GCTCCCGAAGCGTCCGCAGAACCCGACCCTGCGCCGCGAGCTCGTCGAAGTTCCGCTAGTTGAGAAGCTCGAACAGCTC
CCGTTGGAGCCCGCAGGCACAATTATTGCTGCGGCGGCGTAA

4. Supplementary Figures

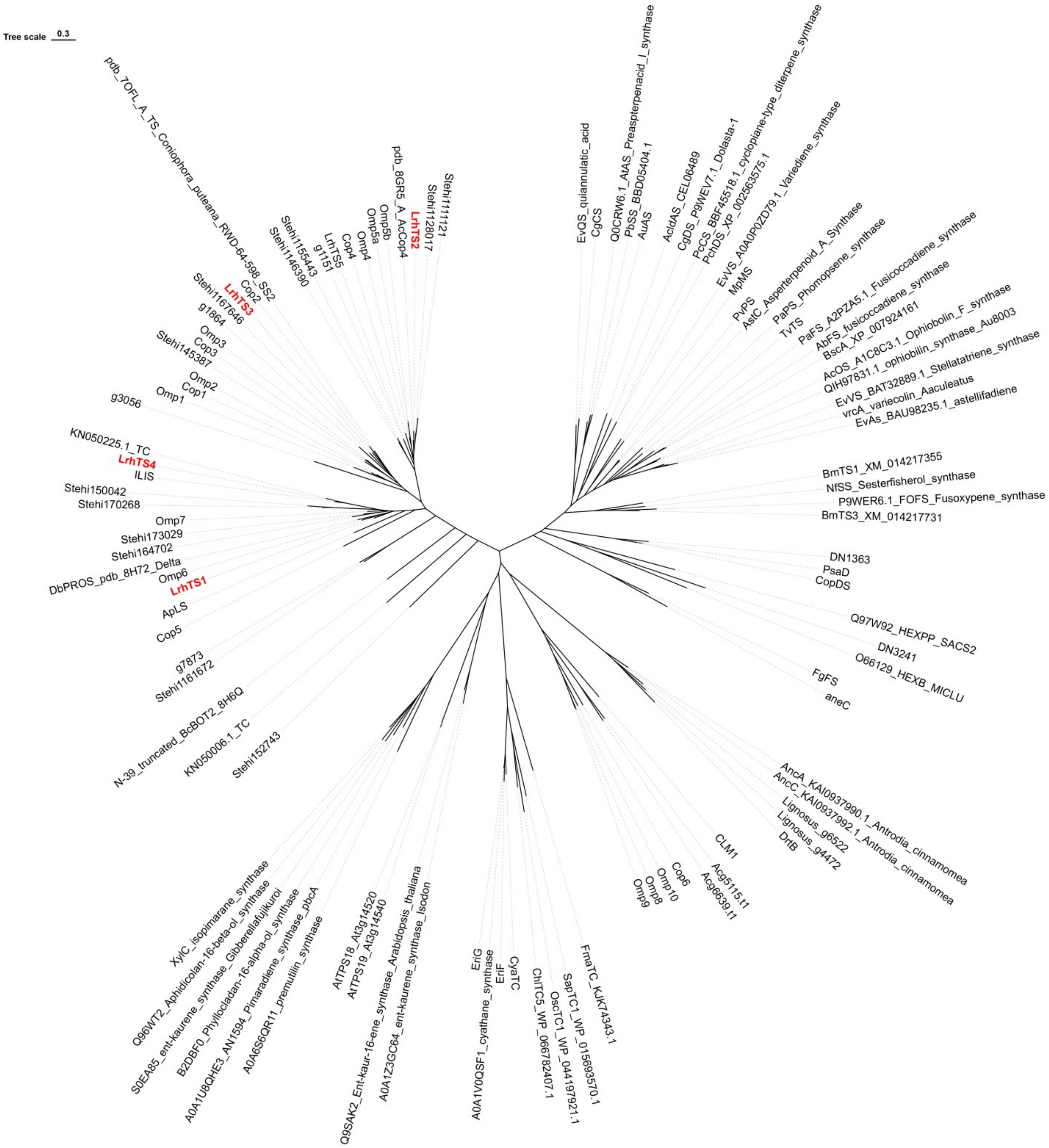

Figure S1. Phylogenetic tree.

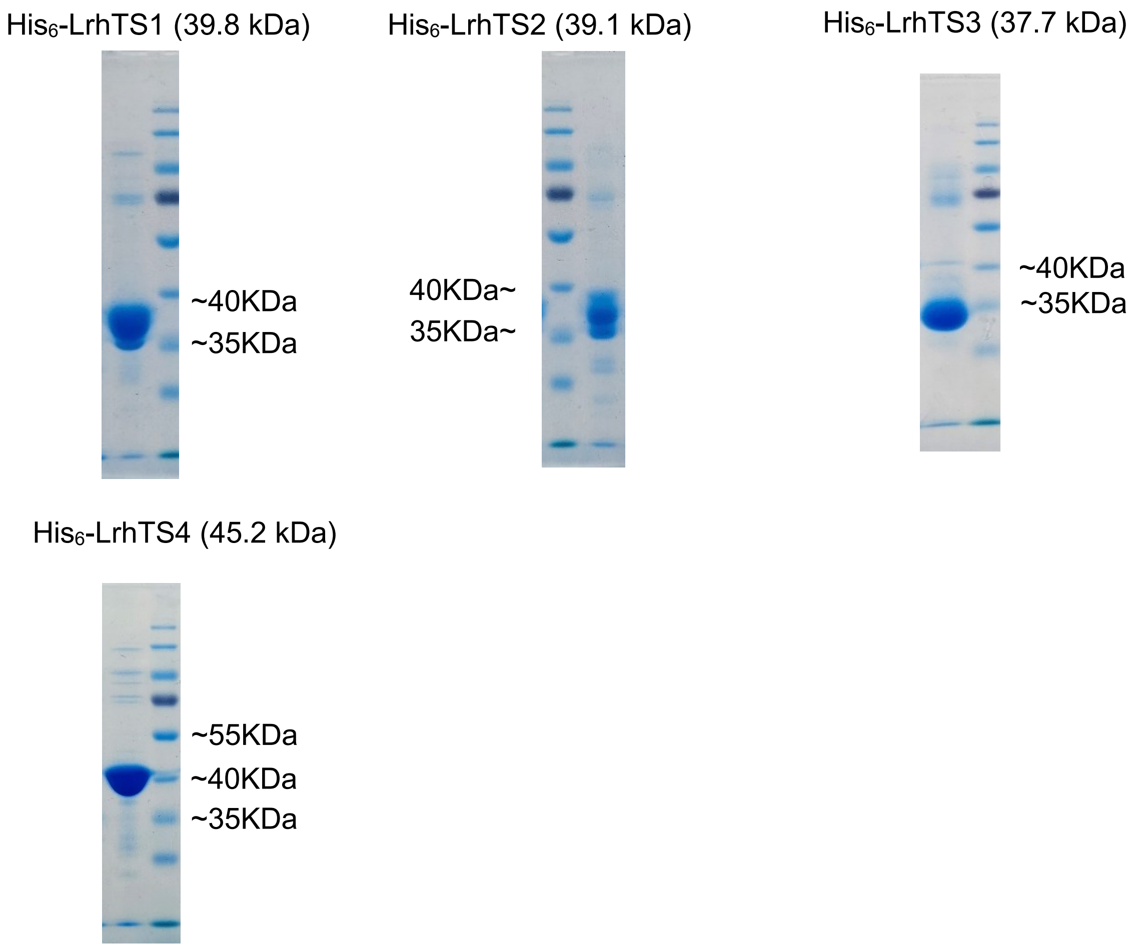

Figure S2. SDS-PAGE analysis of the purified proteins.

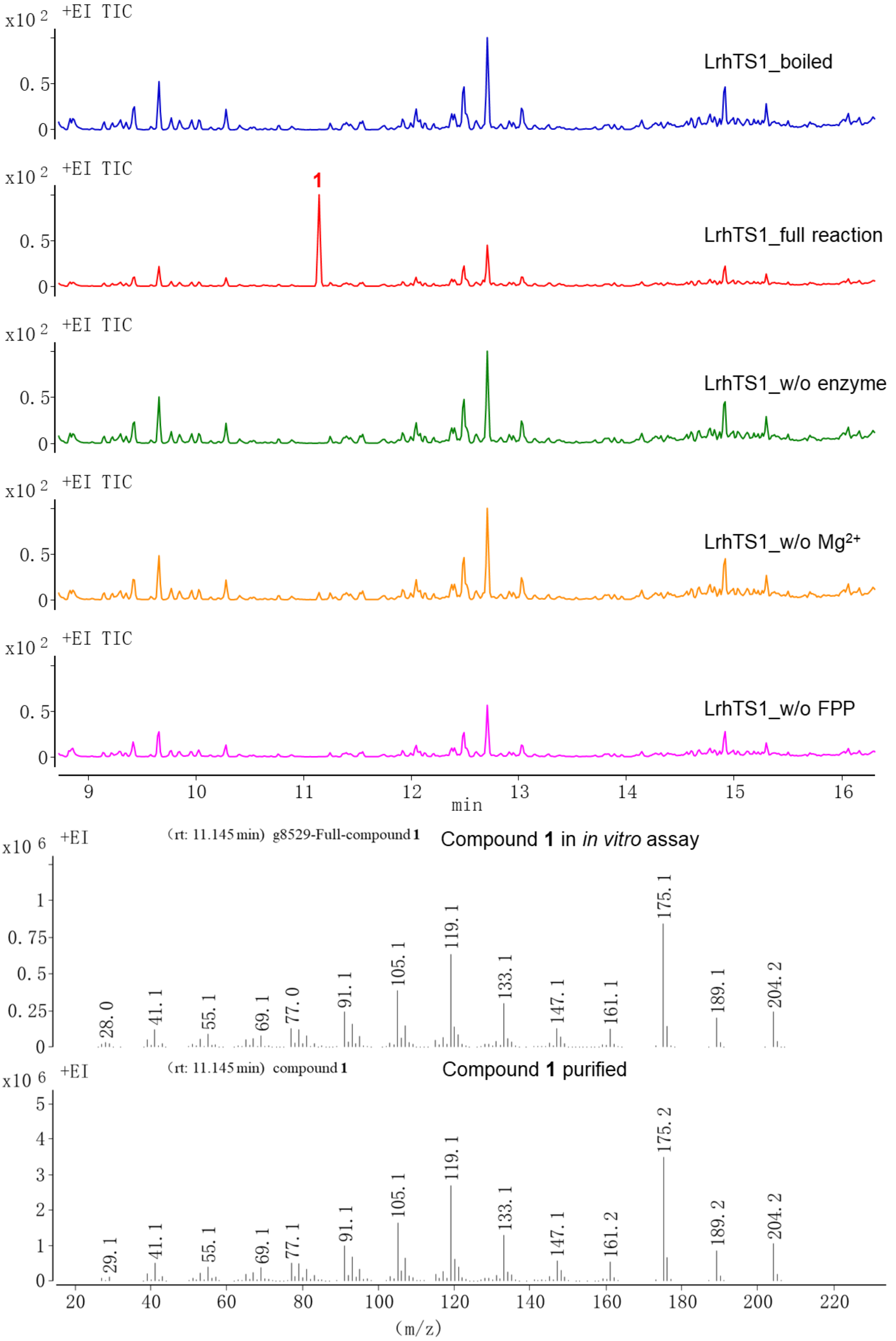

Figure S3. GC-MS chromatograms of the *in vitro* enzymatic assay of LrhTS1 and the EI-MS of the product.

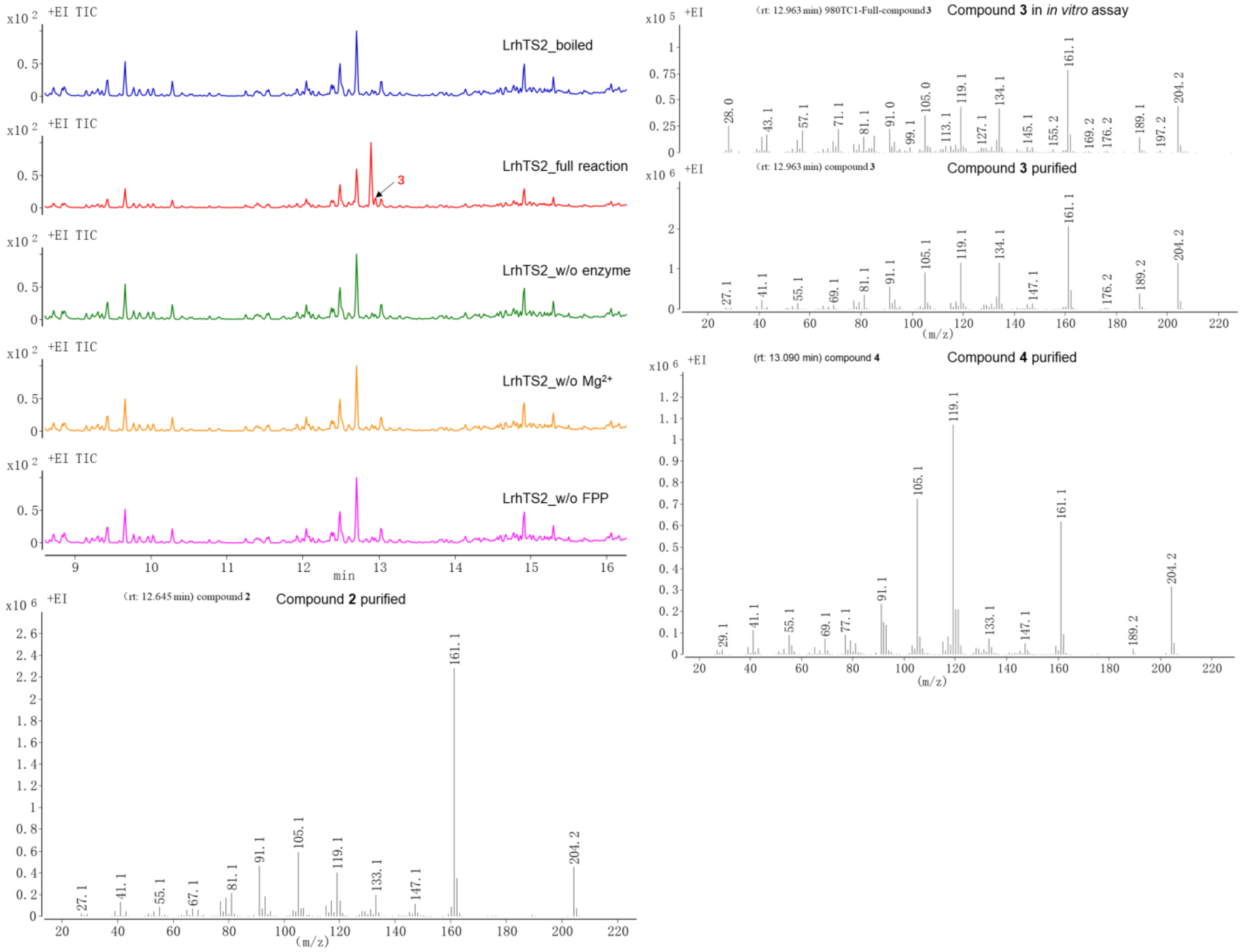

Figure S4. GC-MS chromatograms of the *in vitro* enzymatic assay of LrhTS2 and the EI-MS of the products.

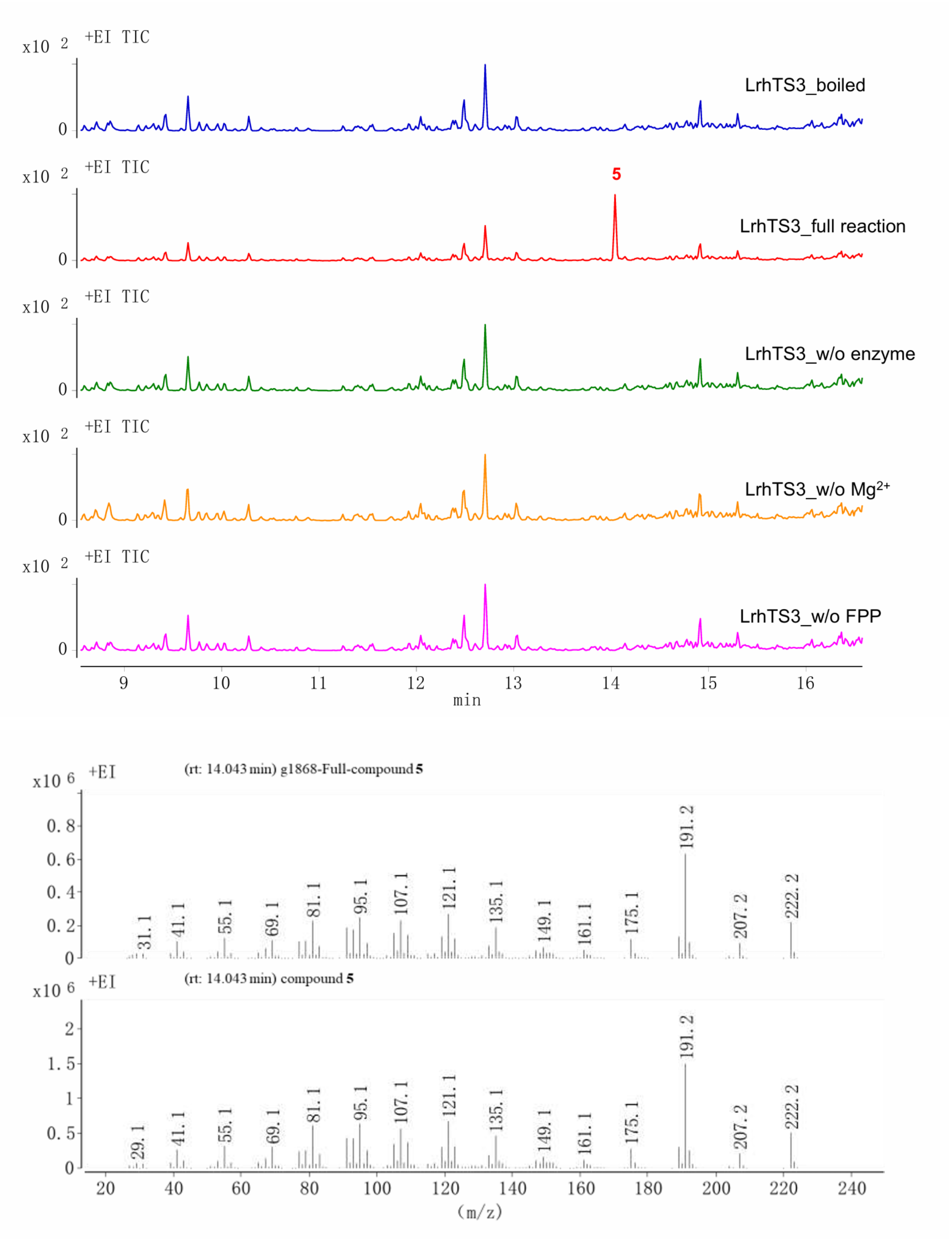

Figure S5. GC-MS chromatograms of the *in vitro* enzymatic assay of LrhTS3 and the EI-MS of the product.

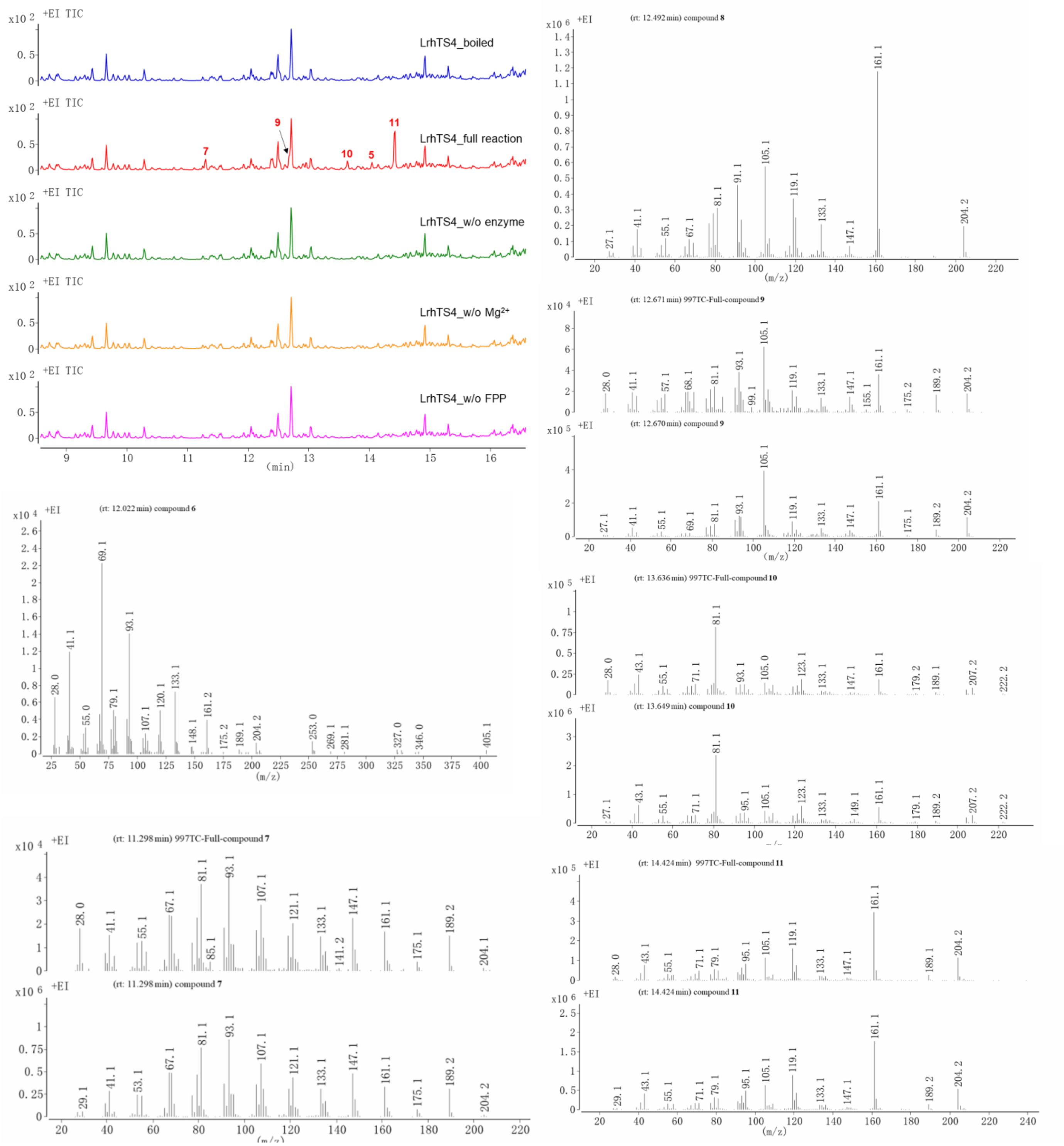

Figure S6. GC-MS chromatograms of the *in vitro* enzymatic assay of LrhTS4 and the EI-MS of the products.

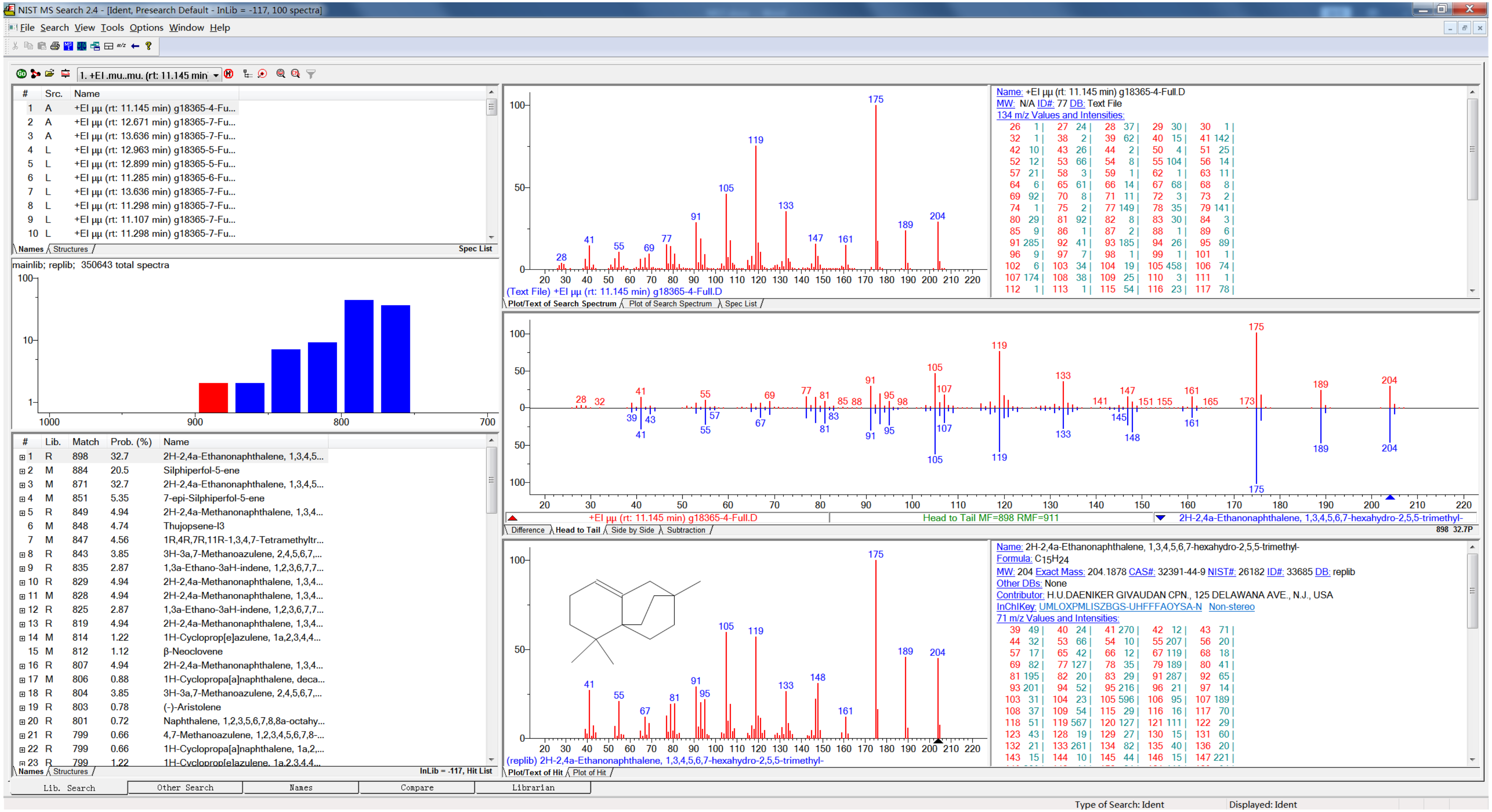

Figure S7. Prediction results of compound **1** mass spectrometry using the NIST database.

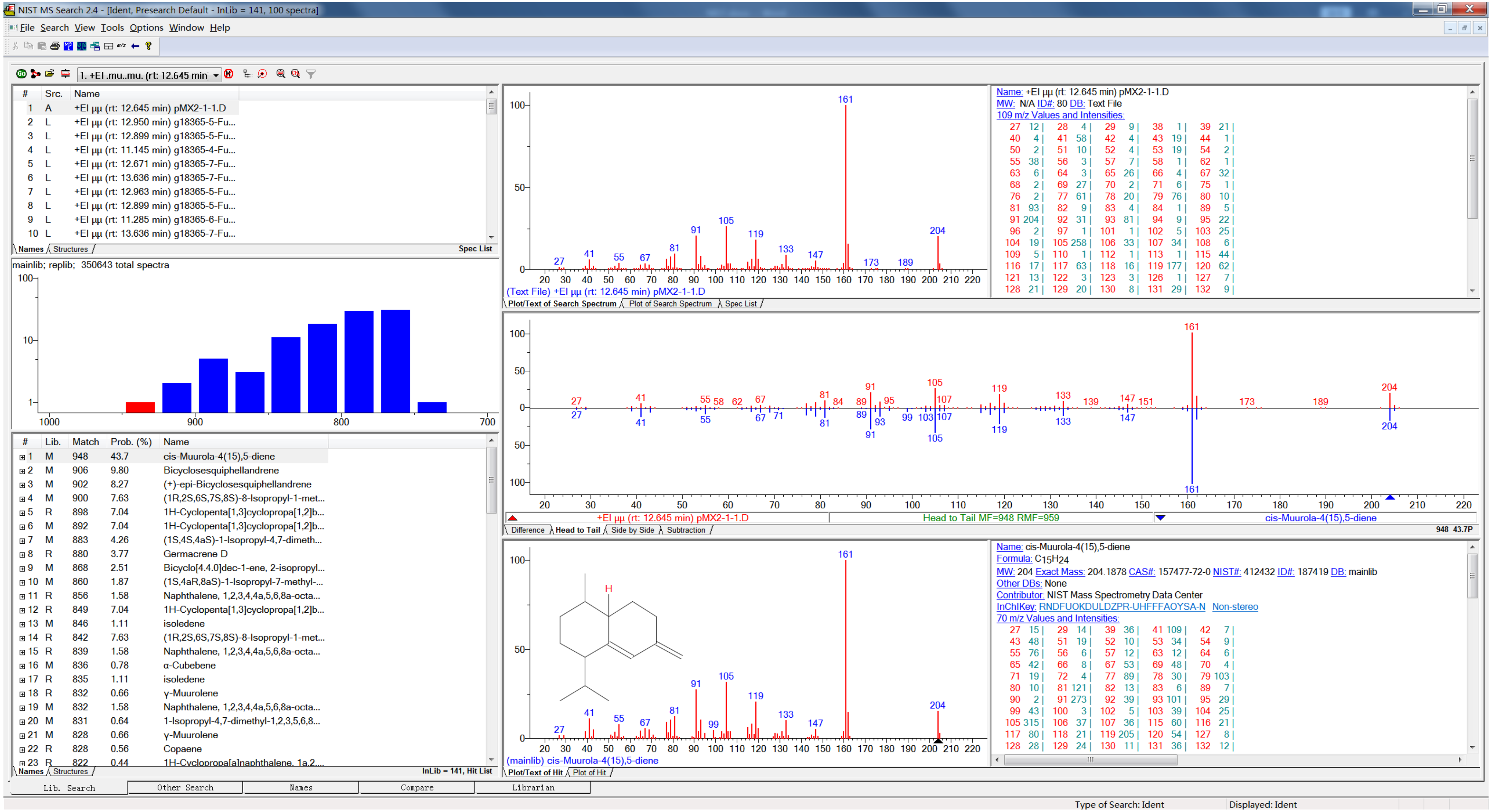

Figure S8. Prediction results of compound **2** mass spectrometry using the NIST database.

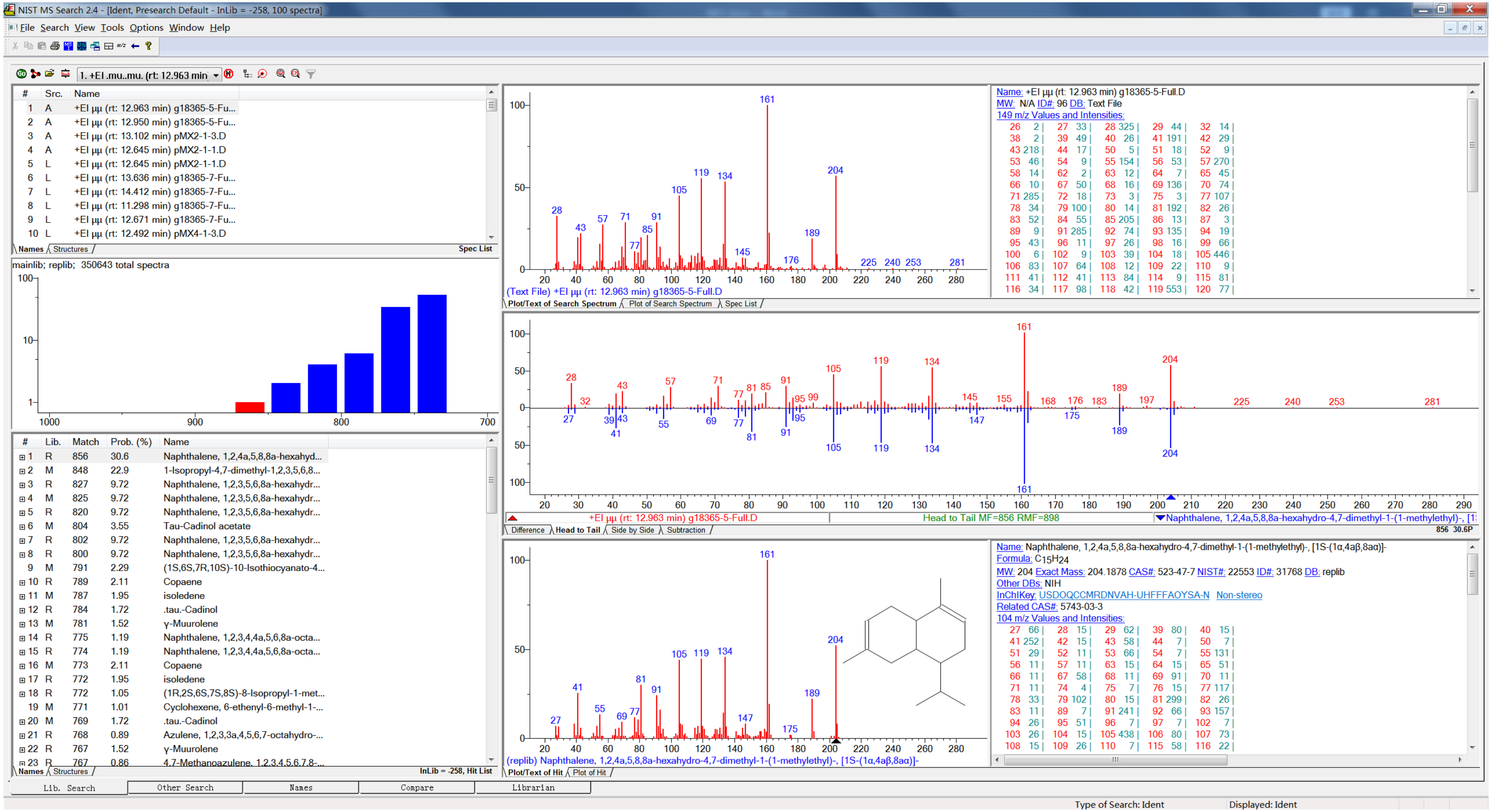

Figure S9. Prediction results of compound **3** mass spectrometry using the NIST database.

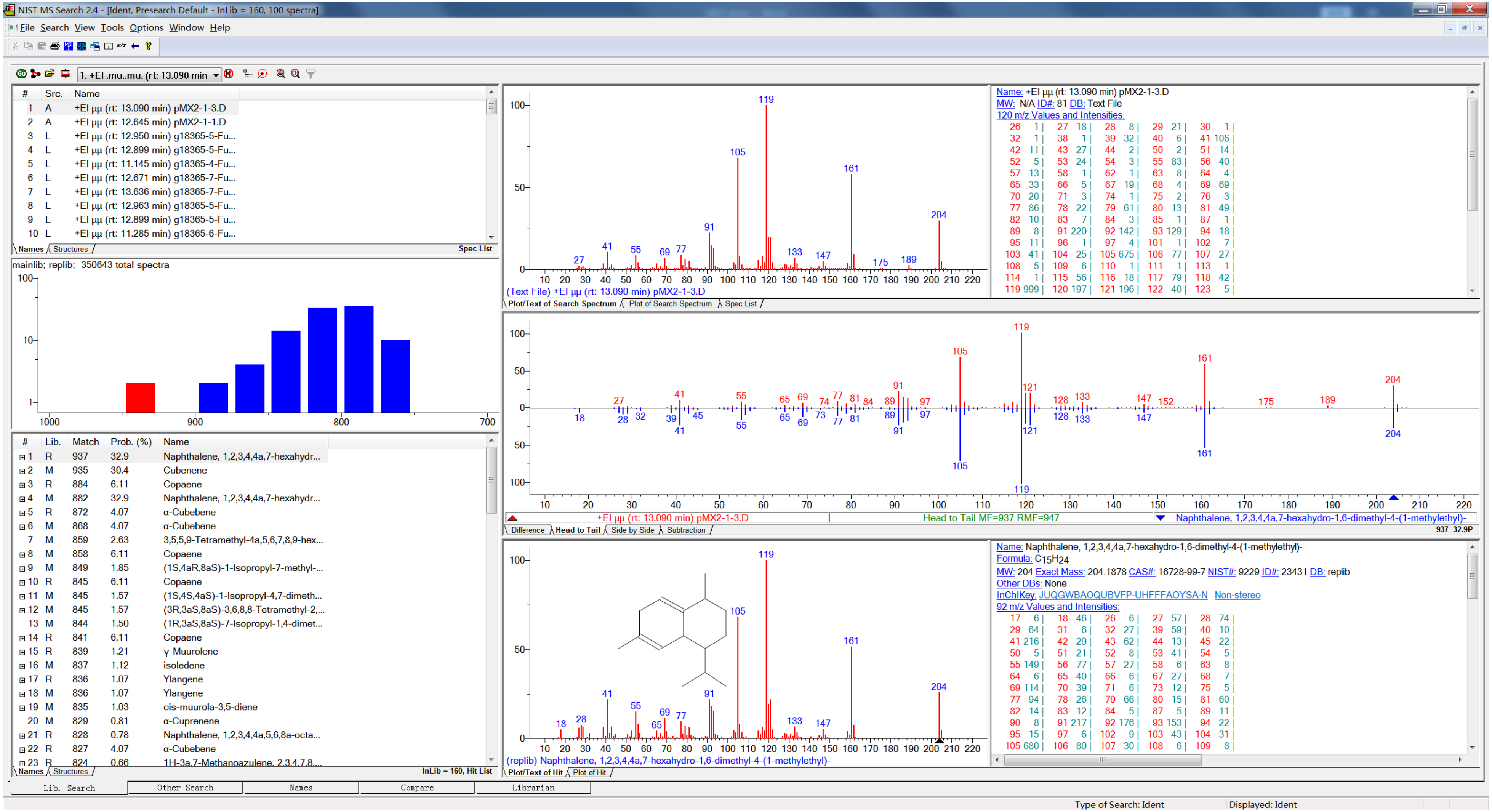

Figure S10. Prediction results of compound **4** mass spectrometry using the NIST database.

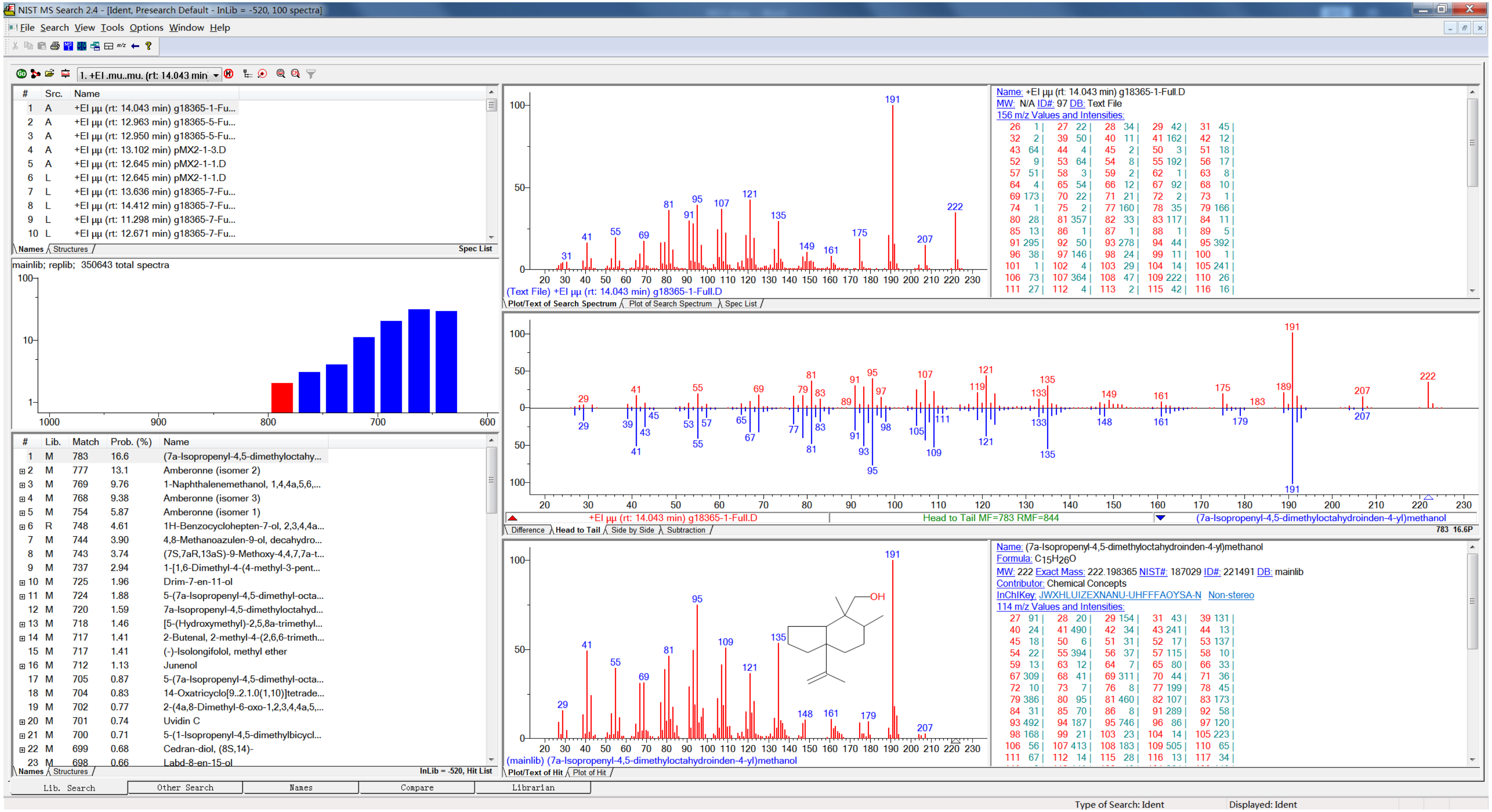

Figure S11. Prediction results of compound **5** mass spectrometry using the NIST database.

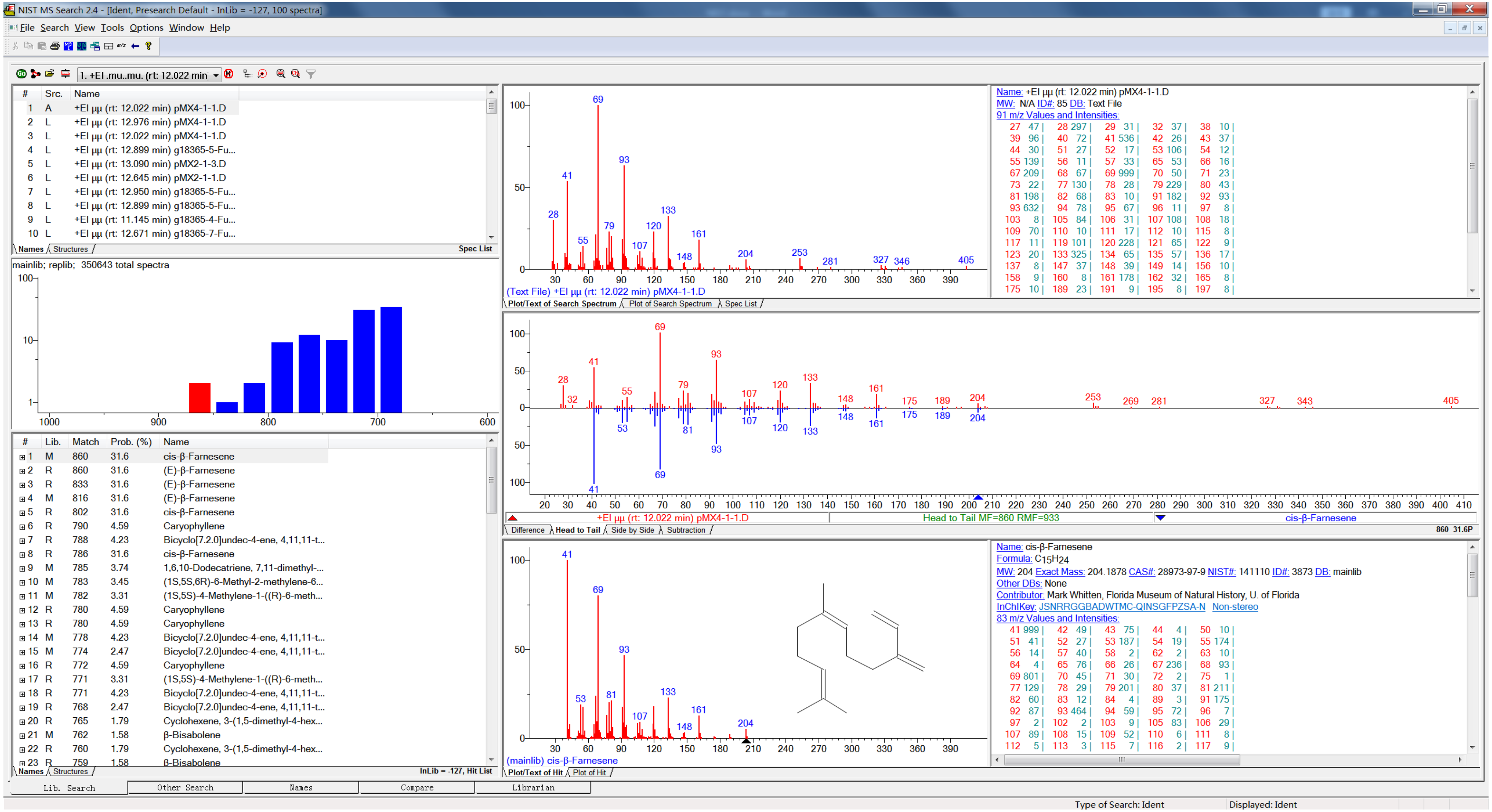

Figure S12. Prediction results of compound **6** mass spectrometry using the NIST database.

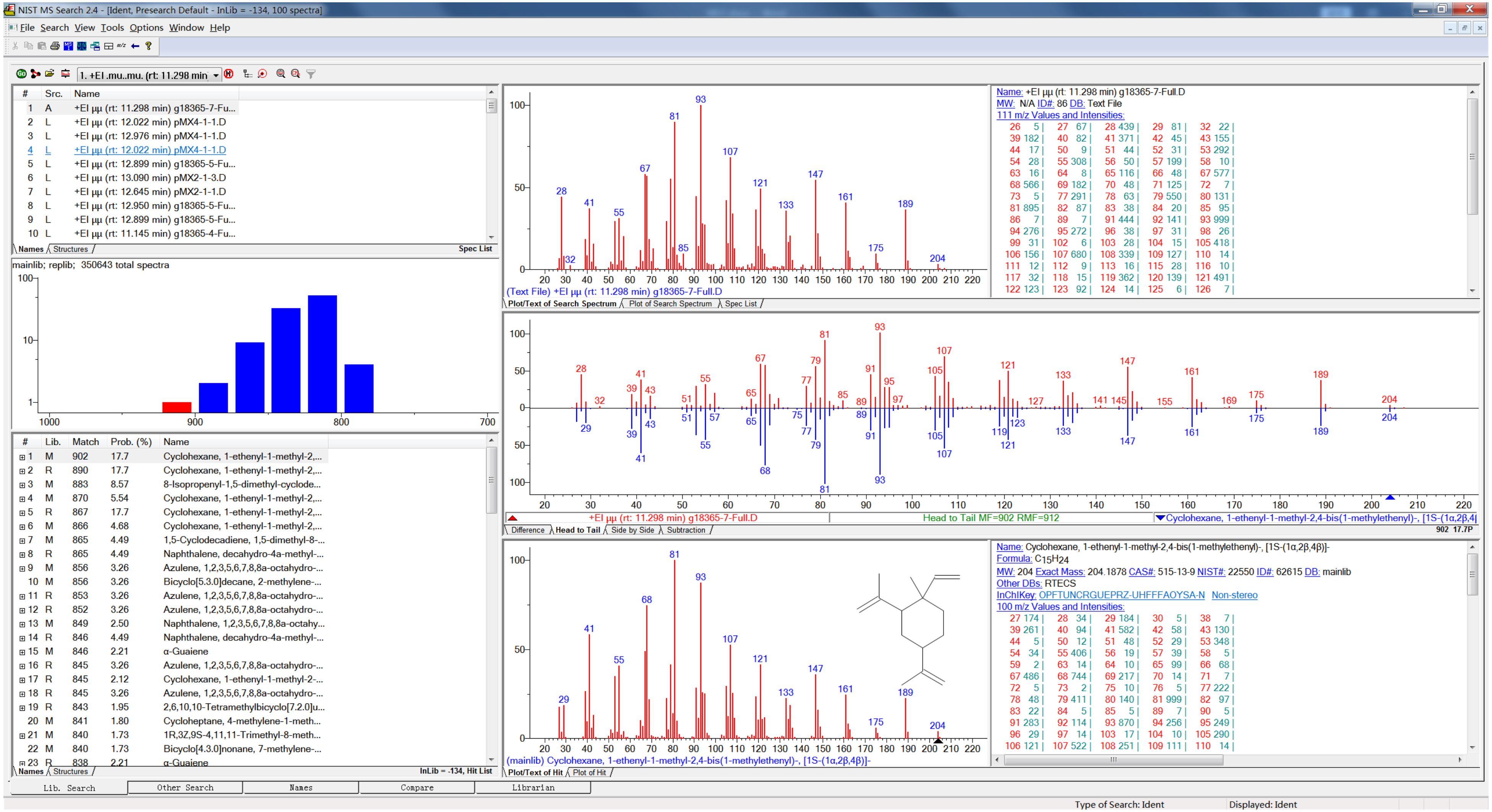

Figure S13. Prediction results of compound **7** mass spectrometry using the NIST database.

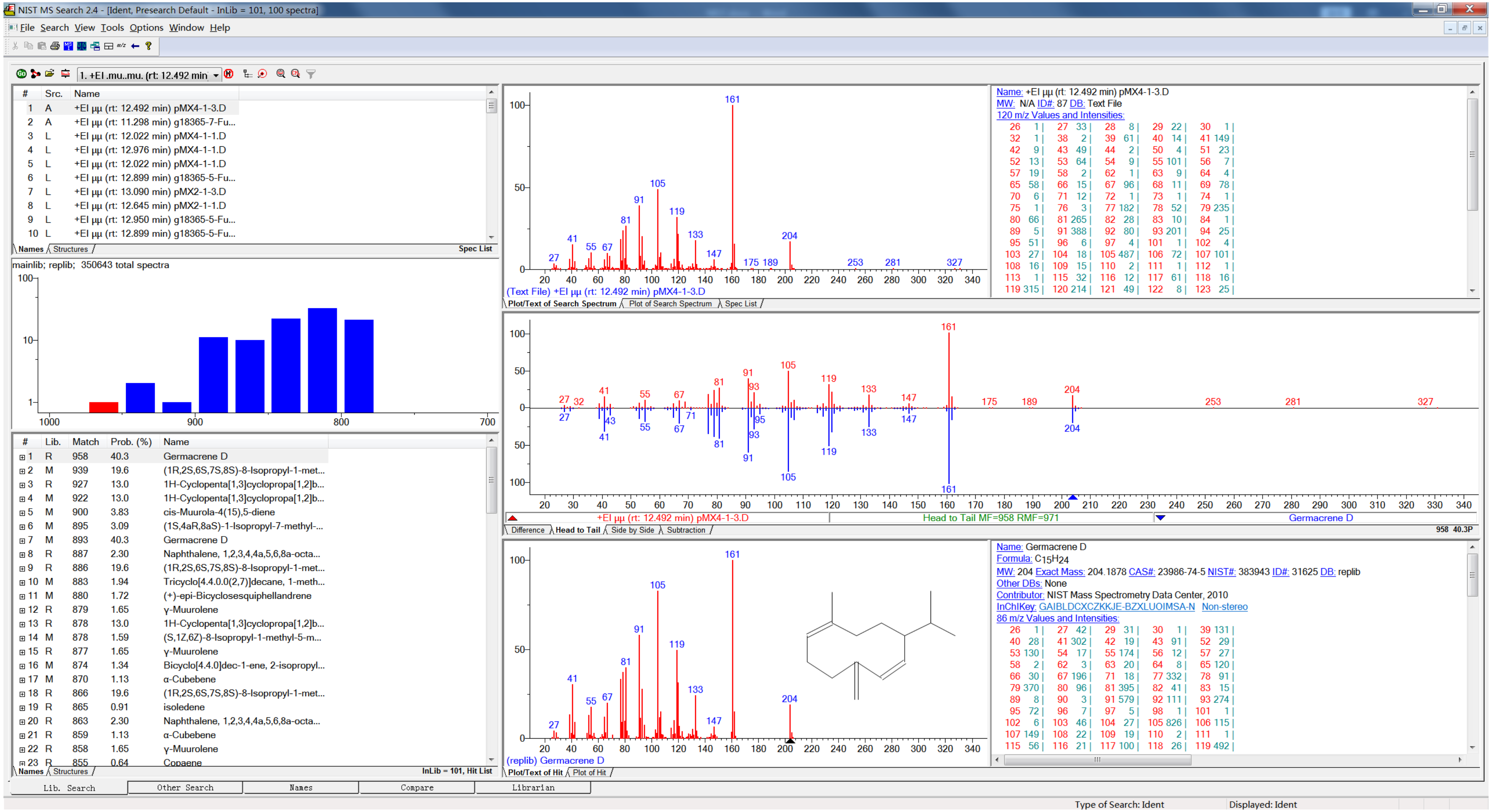

Figure S14. Prediction results of compound **8** mass spectrometry using the NIST database.

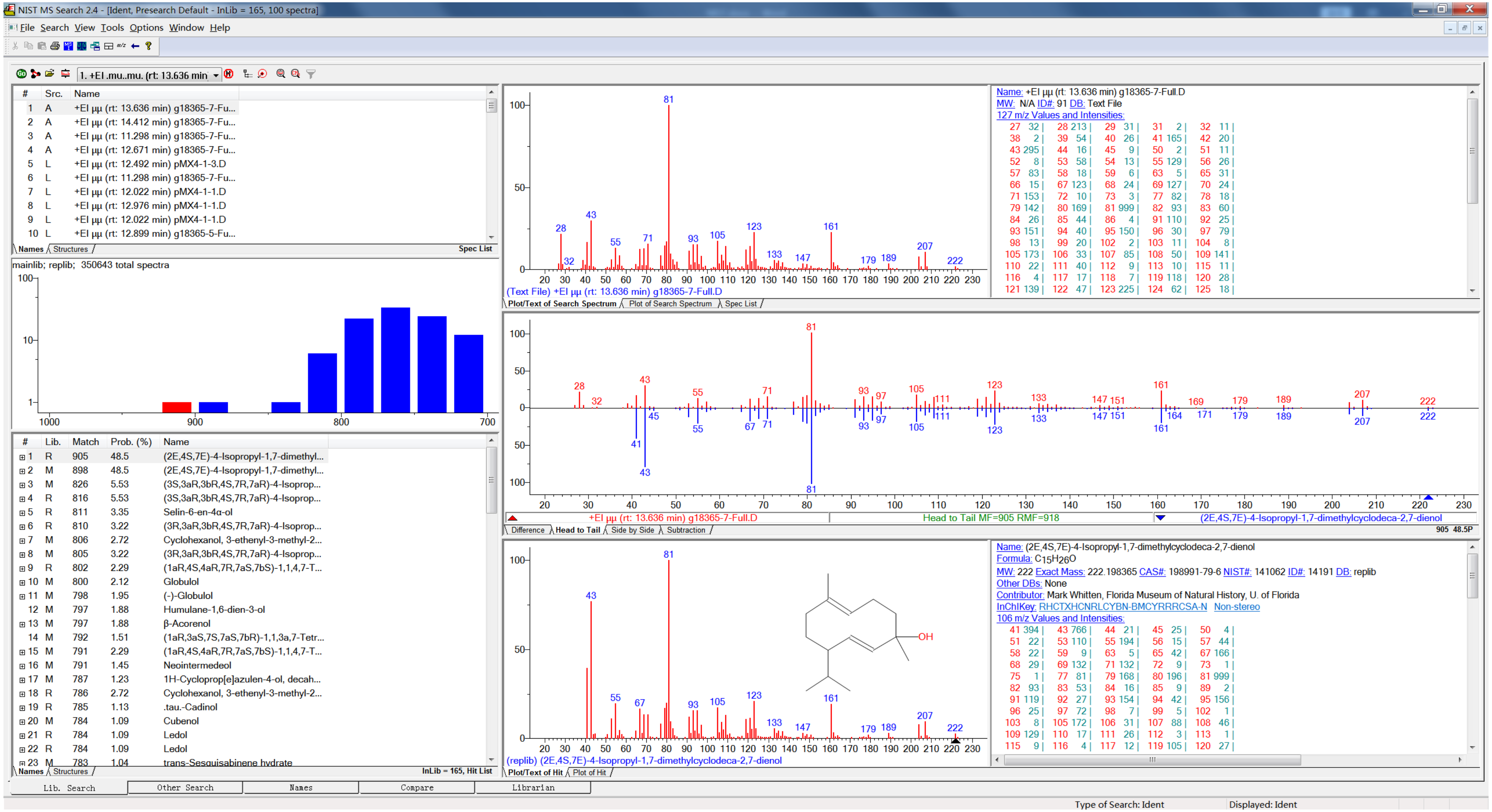

Figure S15. Prediction results of compound **9** mass spectrometry using the NIST database.

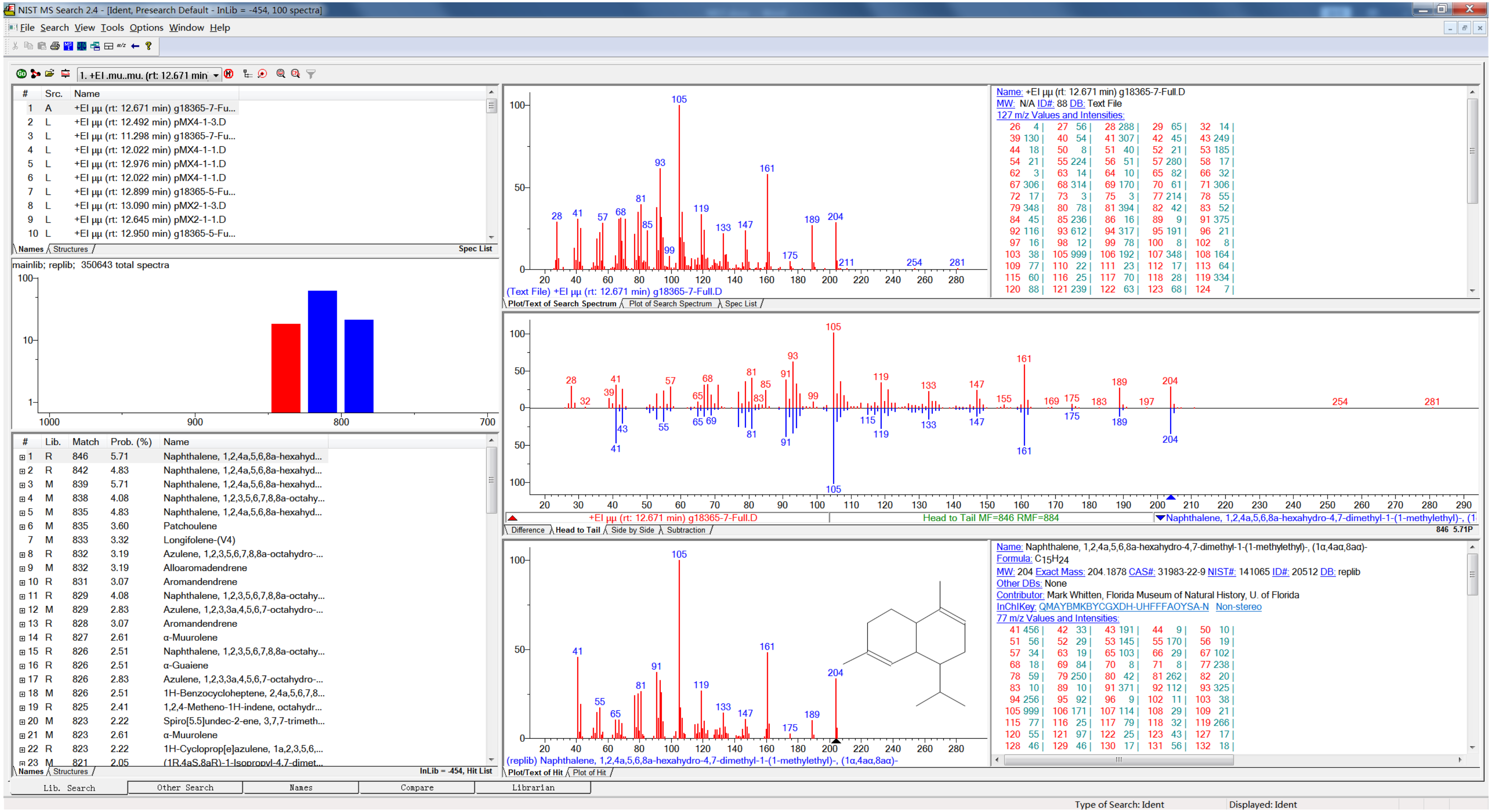

Figure S16. Prediction results of compound **10** mass spectrometry using the NIST database.

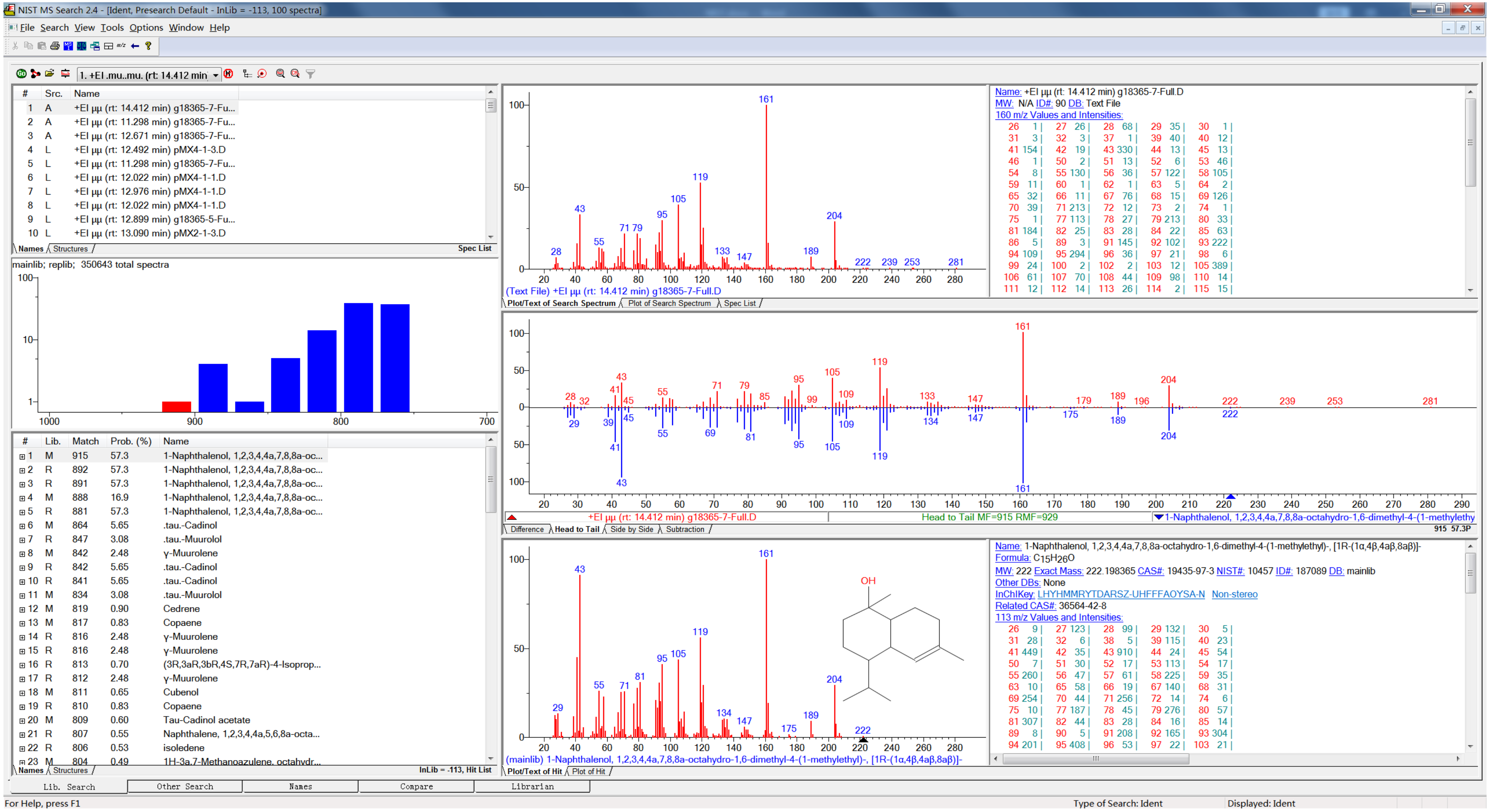

Figure S17. Prediction results of compound **11** mass spectrometry using the NIST database.

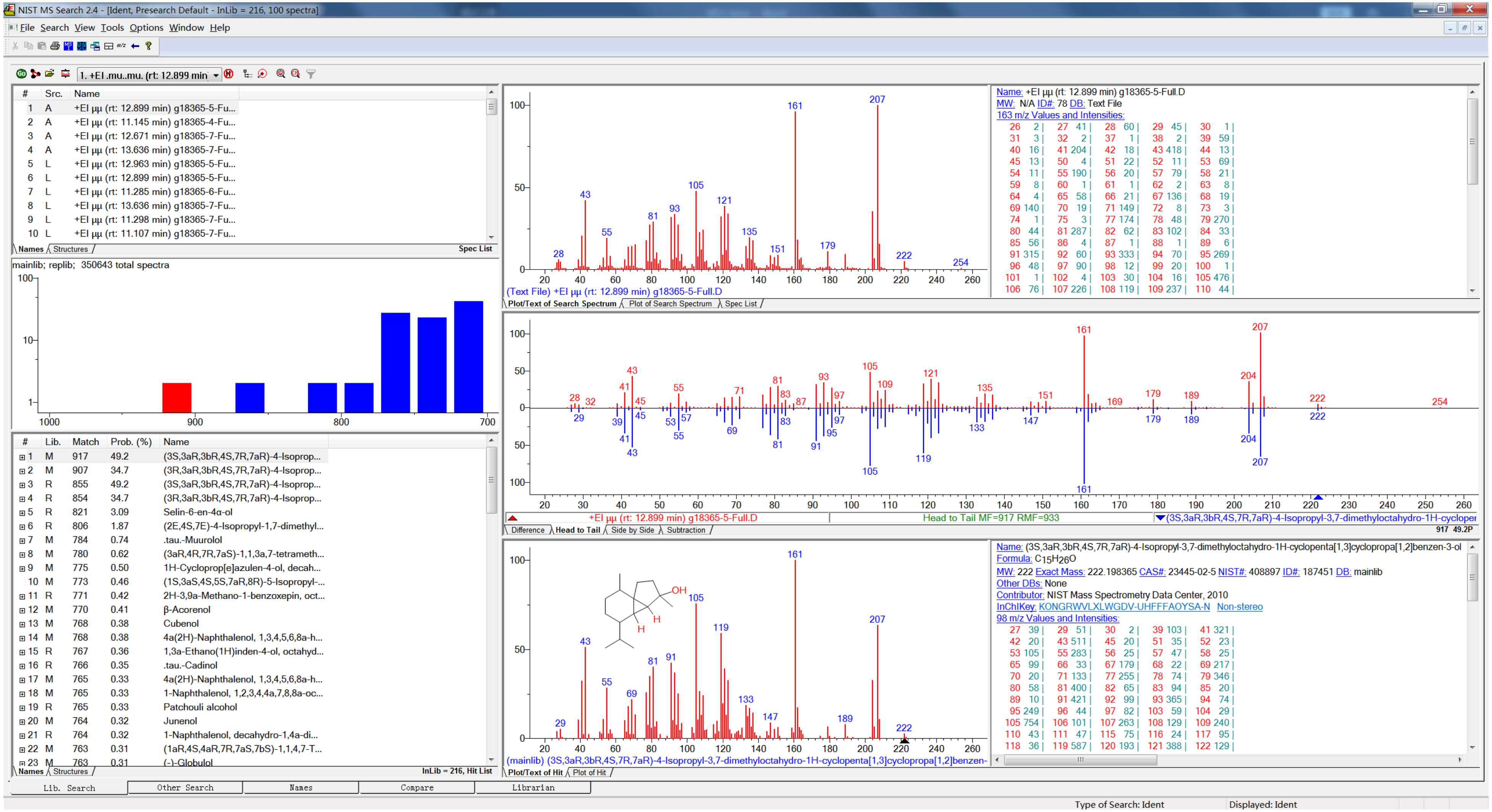

Figure S18. Prediction results of compound marked with asterisk symbols in Figure 2 mass spectrometry using the NIST database.

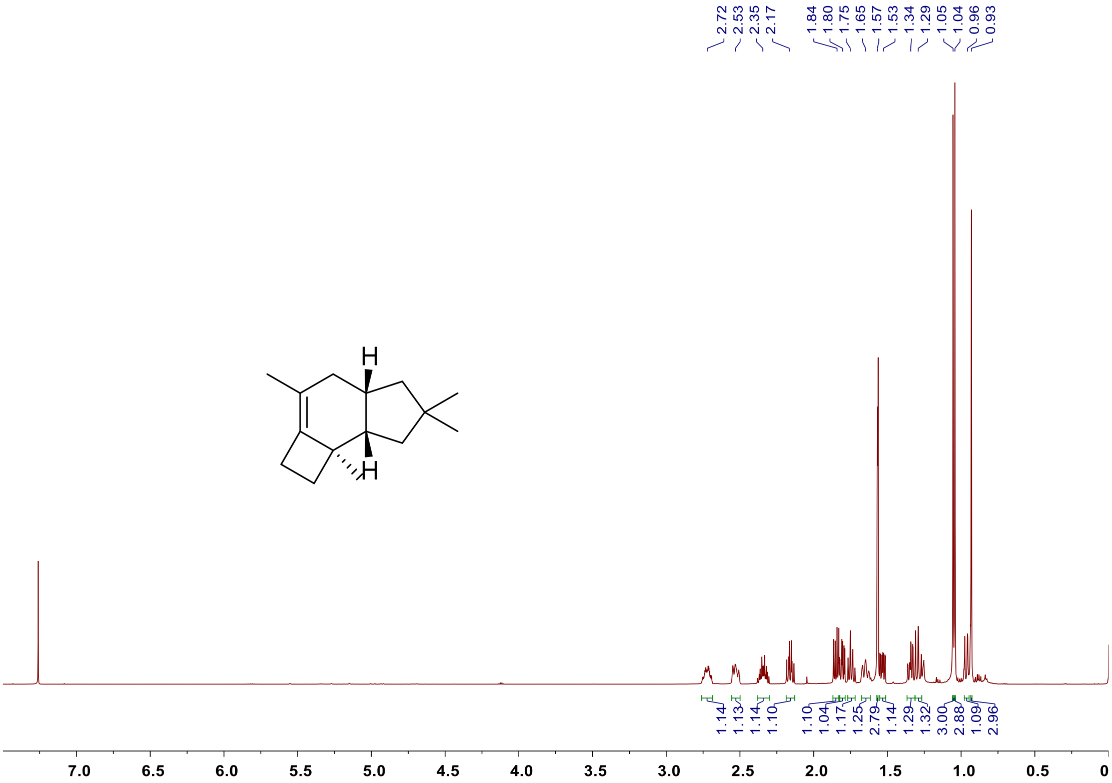

Figure S19. ^{1}H NMR spectrum of **1** in $CDCl_3$ at 600 MHz.

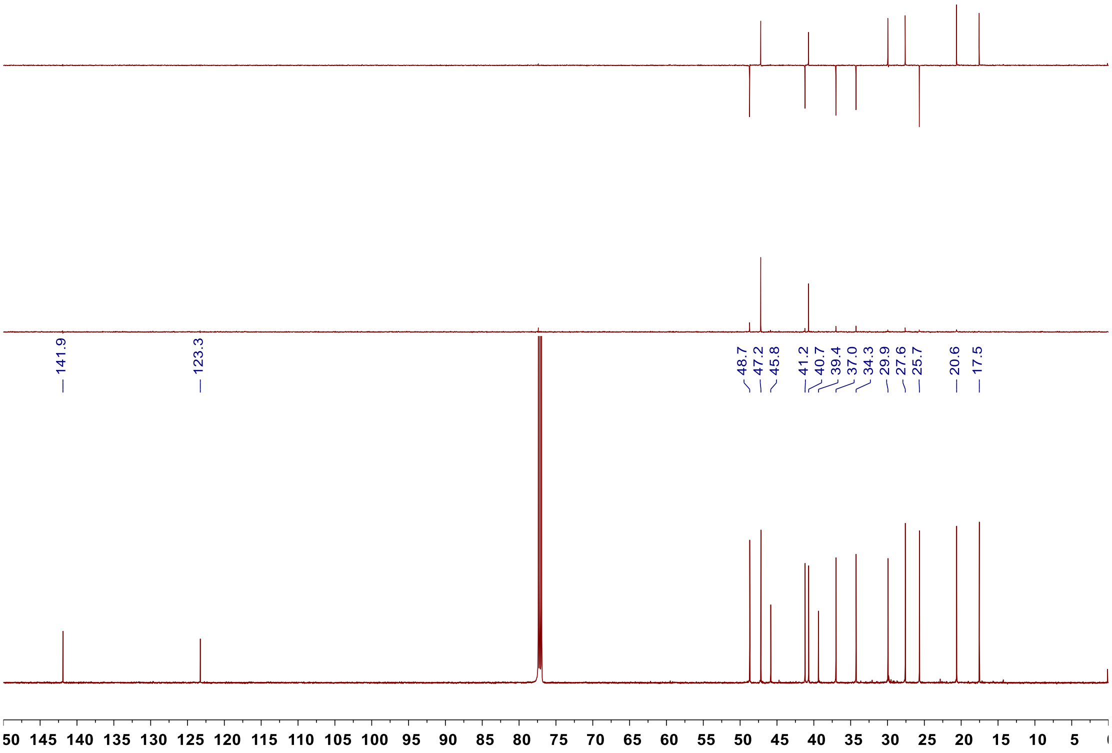

Figure S20. ^{13}C NMR spectrum of **1** in $CDCl_3$ at 600 MHz.

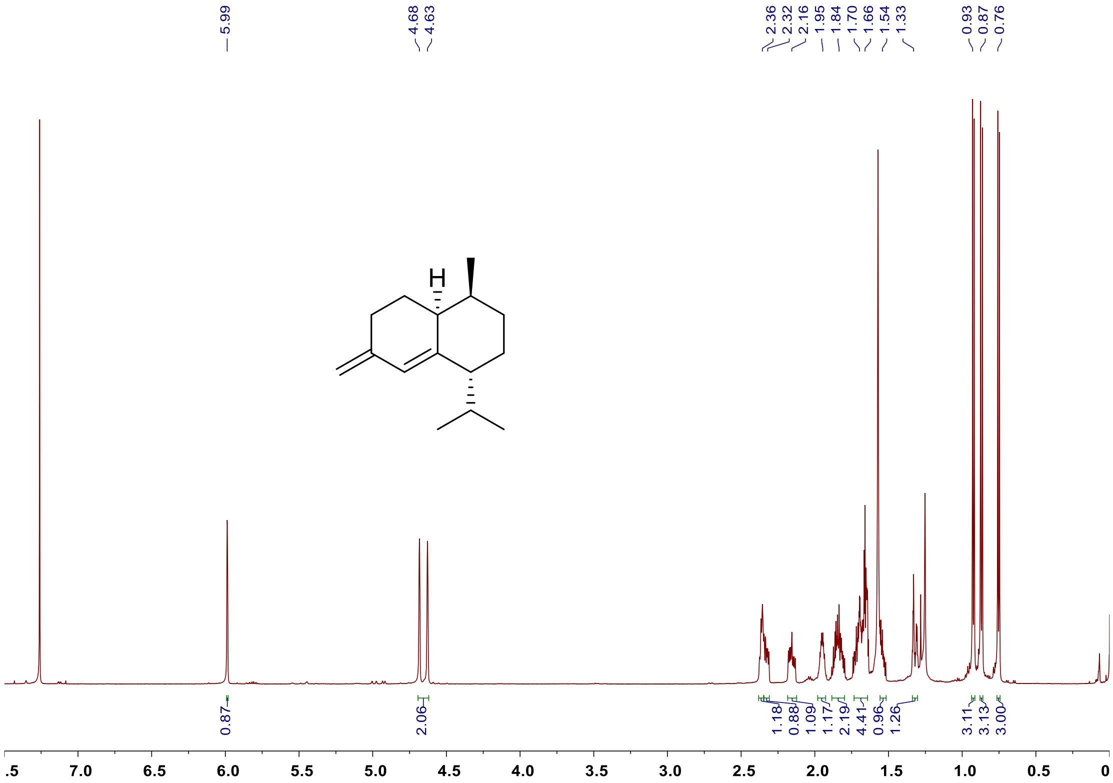

Figure S21. ^{1}H NMR spectrum of **2** in $CDCl_3$ at 600 MHz.

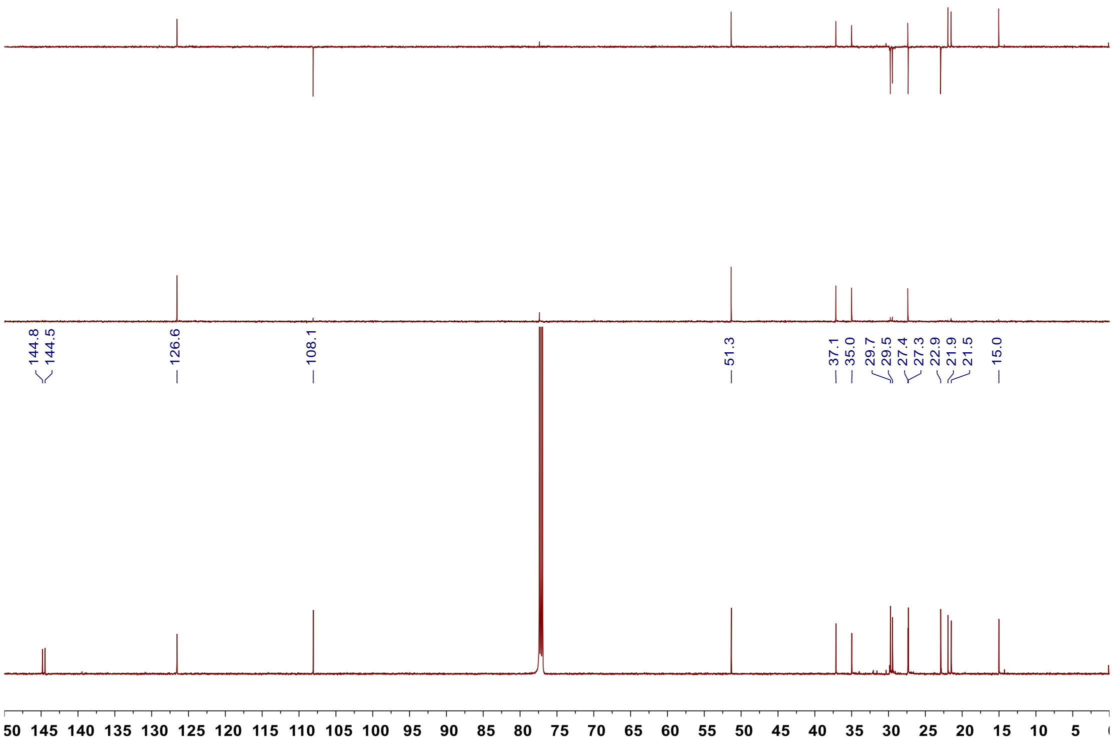

Figure S22. ^{13}C NMR spectrum of **2** in $CDCl_3$ at 600 MHz.

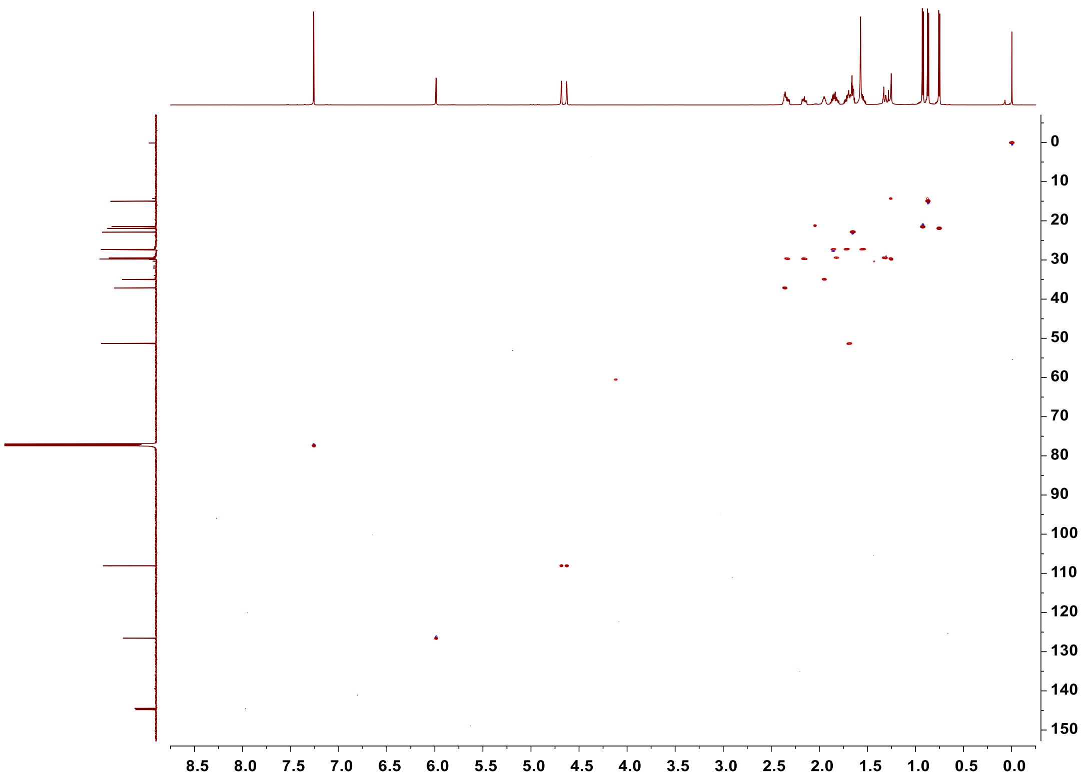

Figure S23. HSQC spectrum of **2** in $CDCl_3$ at 600 MHz.

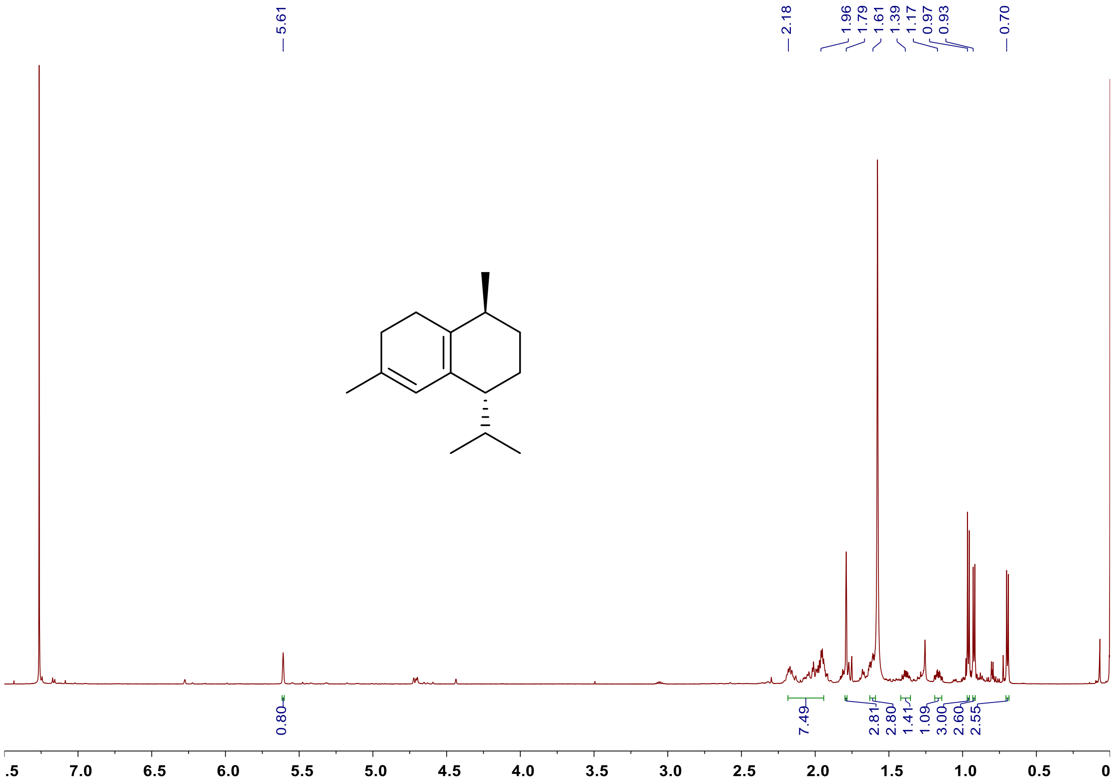

Figure S24. ^{1}H NMR spectrum of **2a** in $CDCl_3$ at 600 MHz.

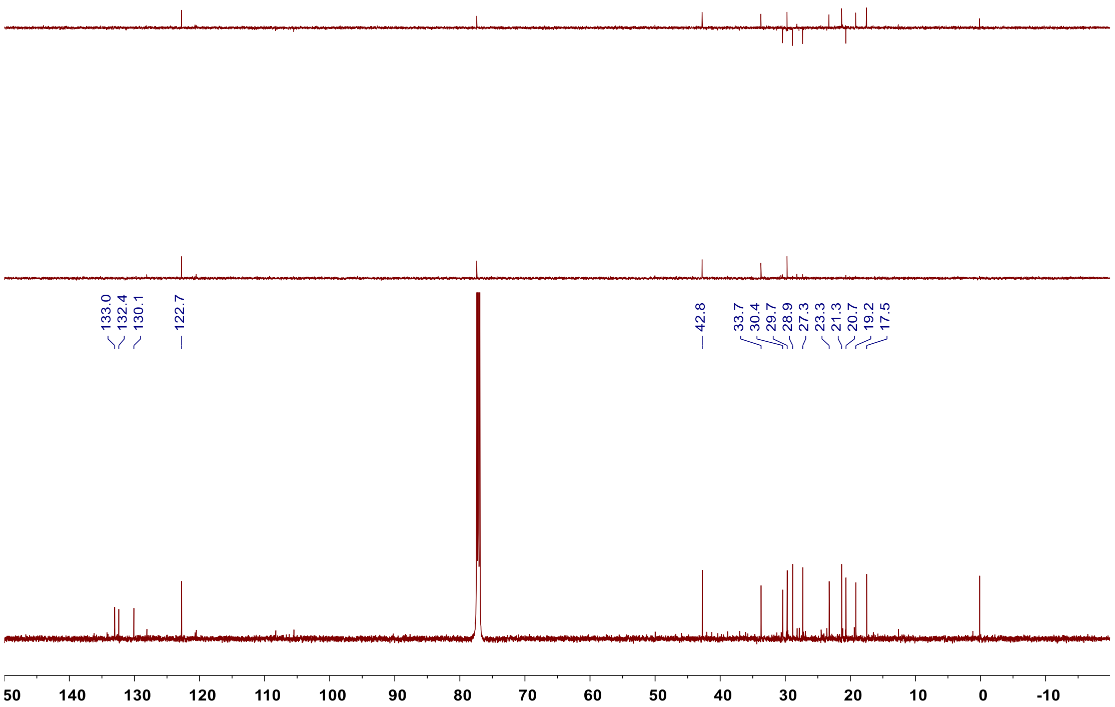

Figure S25. ^{13}C NMR spectrum of **2a** in $CDCl_3$ at 600 MHz.

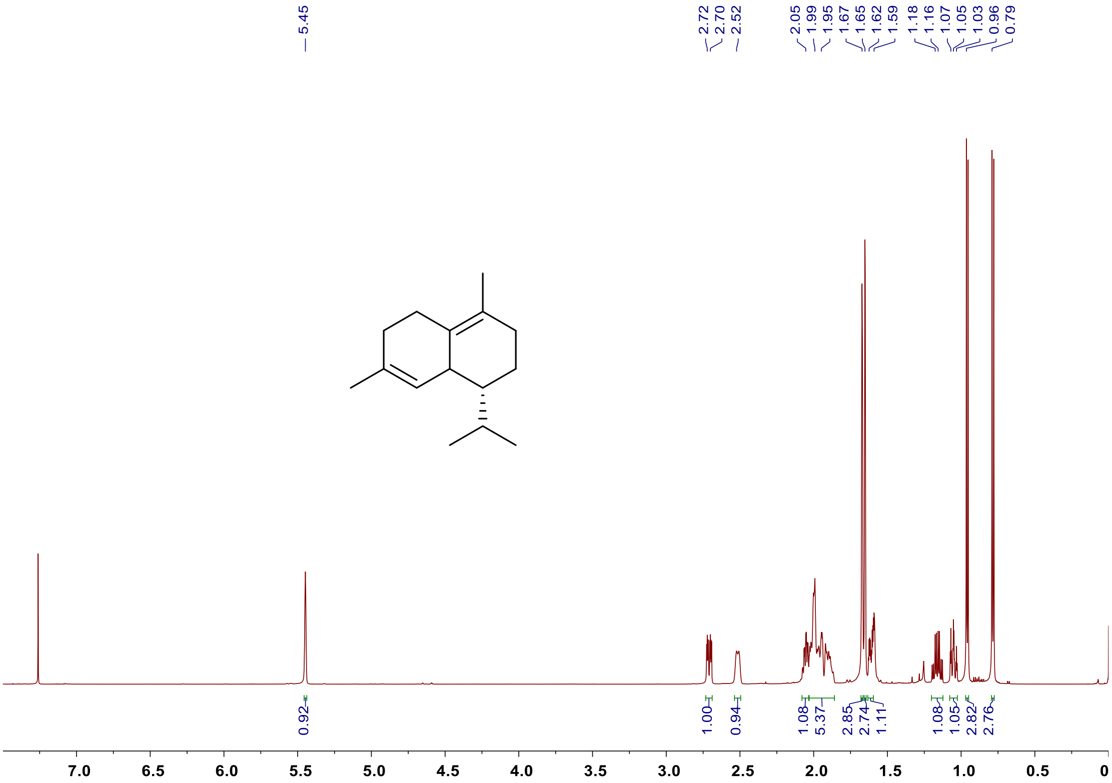

Figure S26. ^{1}H NMR spectrum of **3** in $CDCl_3$ at 600 MHz.

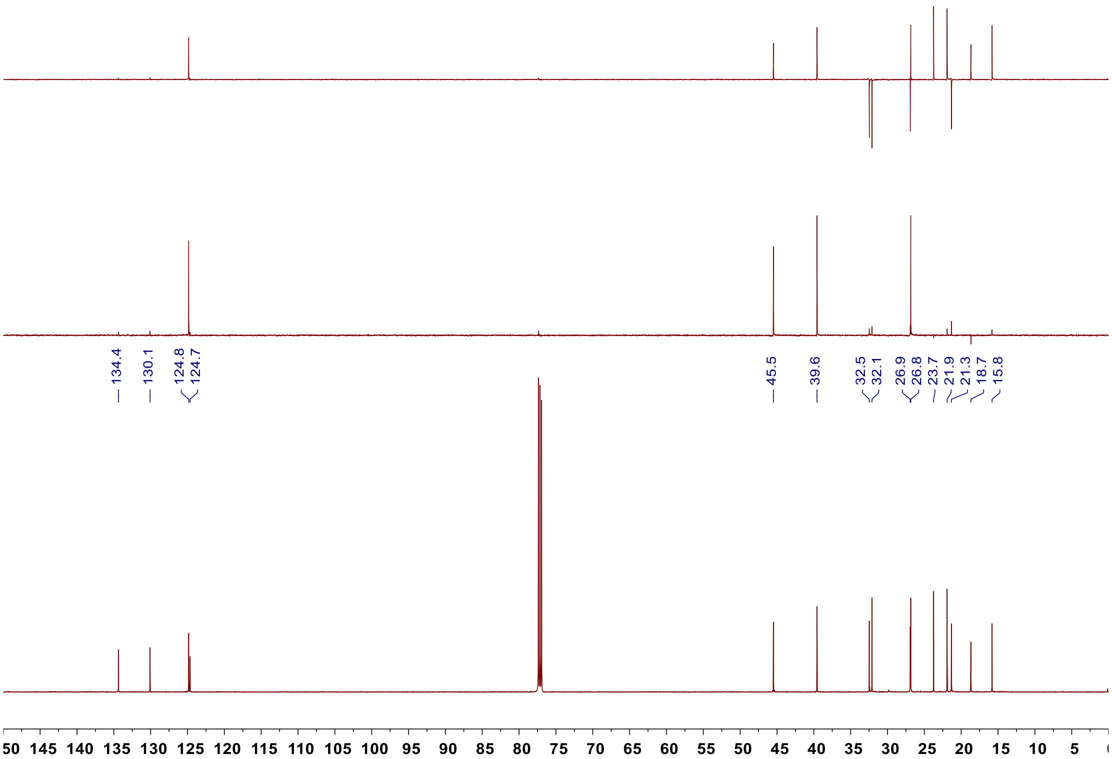

Figure S27. ^{13}C NMR spectrum of **3** in $CDCl_3$ at 600 MHz.

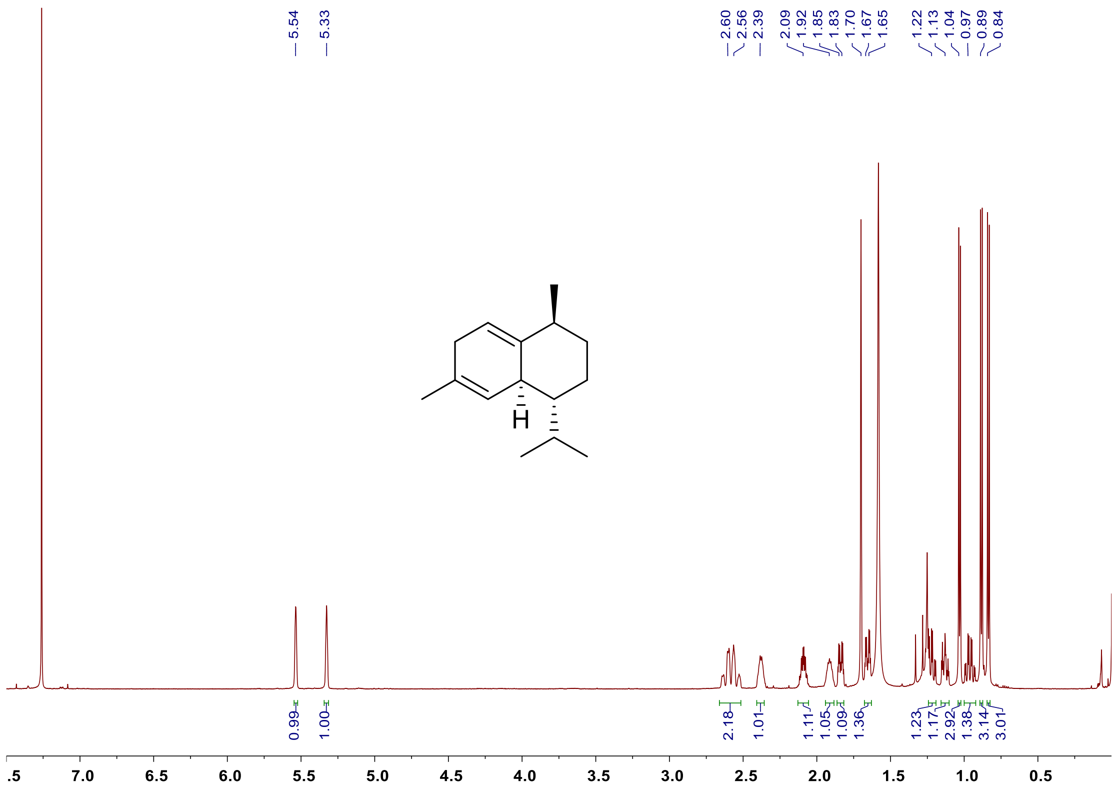

Figure S28. ^{1}H NMR spectrum of **4** in $CDCl_3$ at 600 MHz.

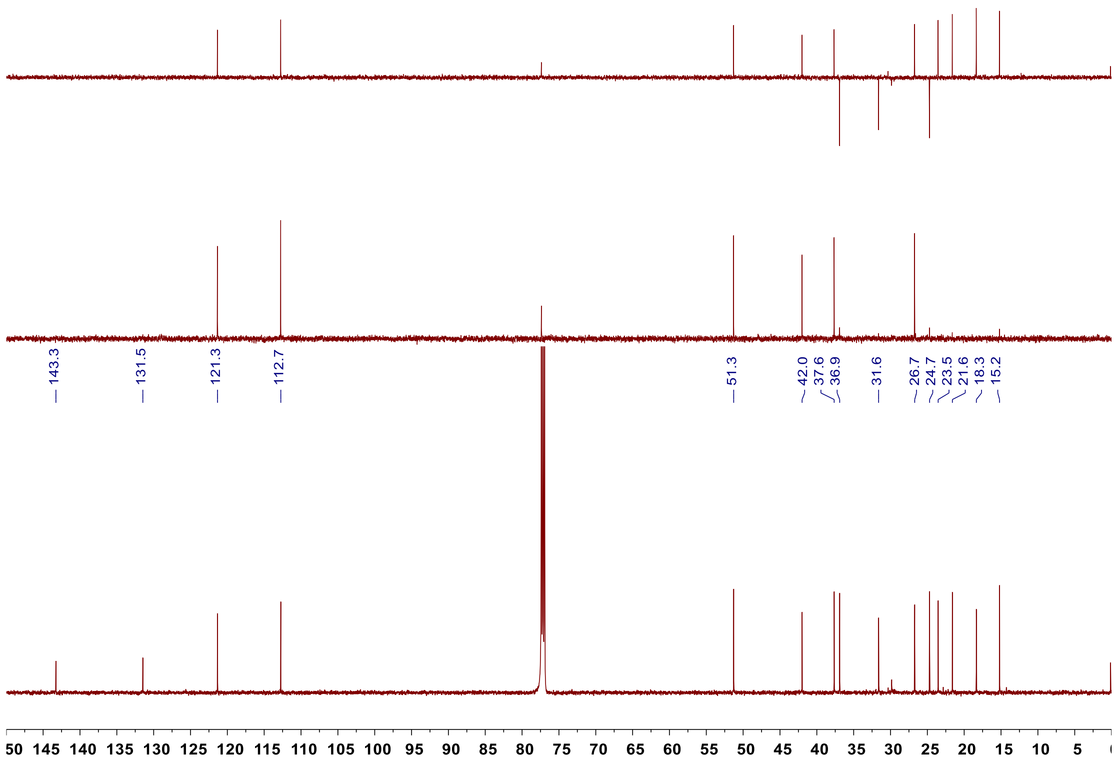

Figure S29. ^{13}C NMR spectrum of **4** in $CDCl_3$ at 600 MHz.

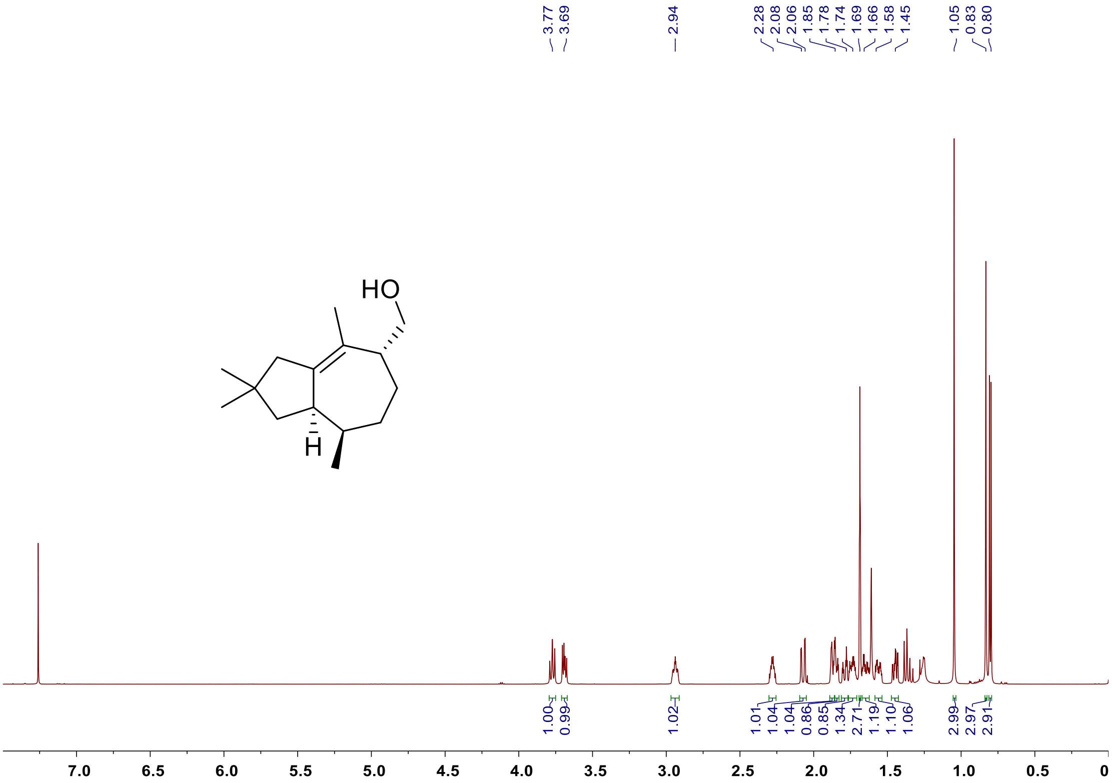

Figure S30. ^{1}H NMR spectrum of **5** in $CDCl_3$ at 600 MHz.

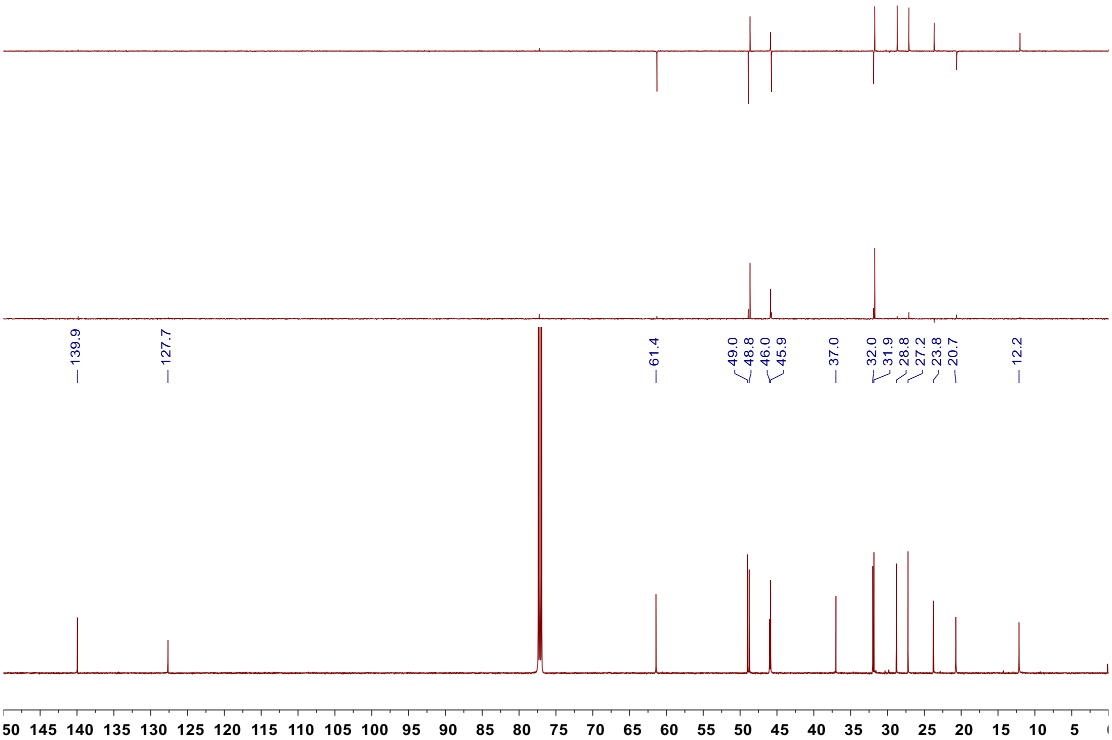

Figure S31. ^{13}C NMR spectrum of **5** in $CDCl_3$ at 600 MHz.

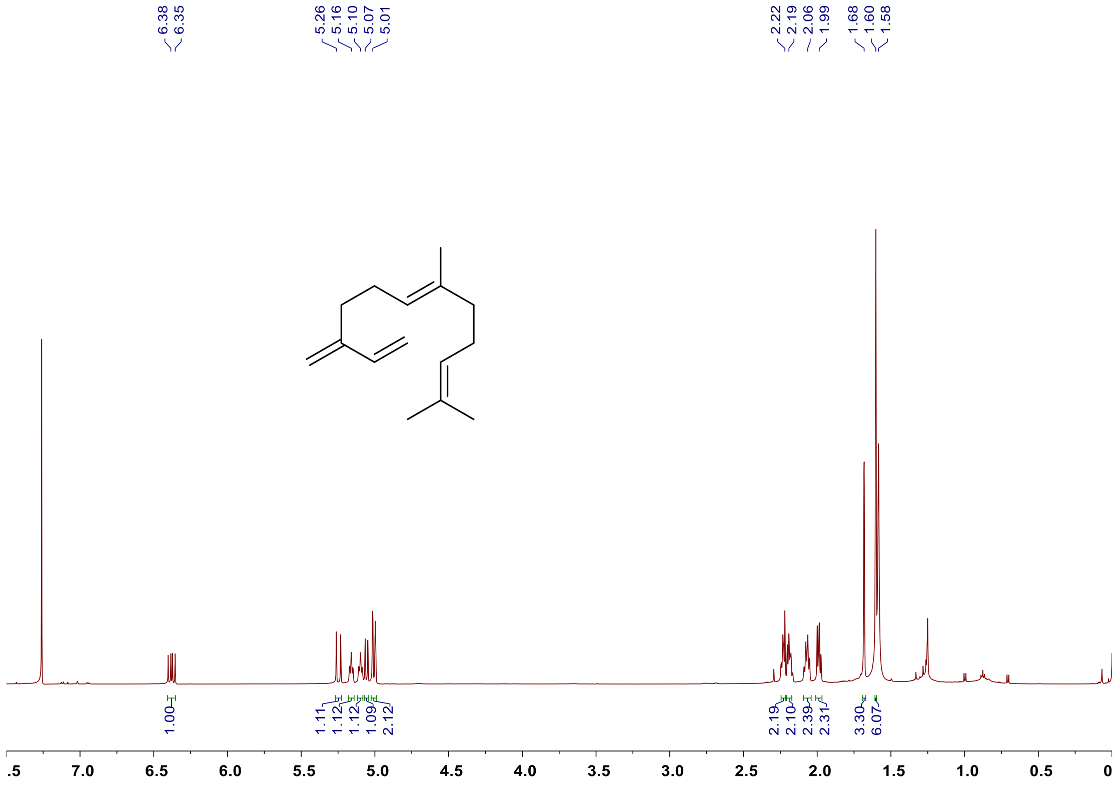

Figure S32. ^{1}H NMR spectrum of **6** in $CDCl_3$ at 600 MHz.

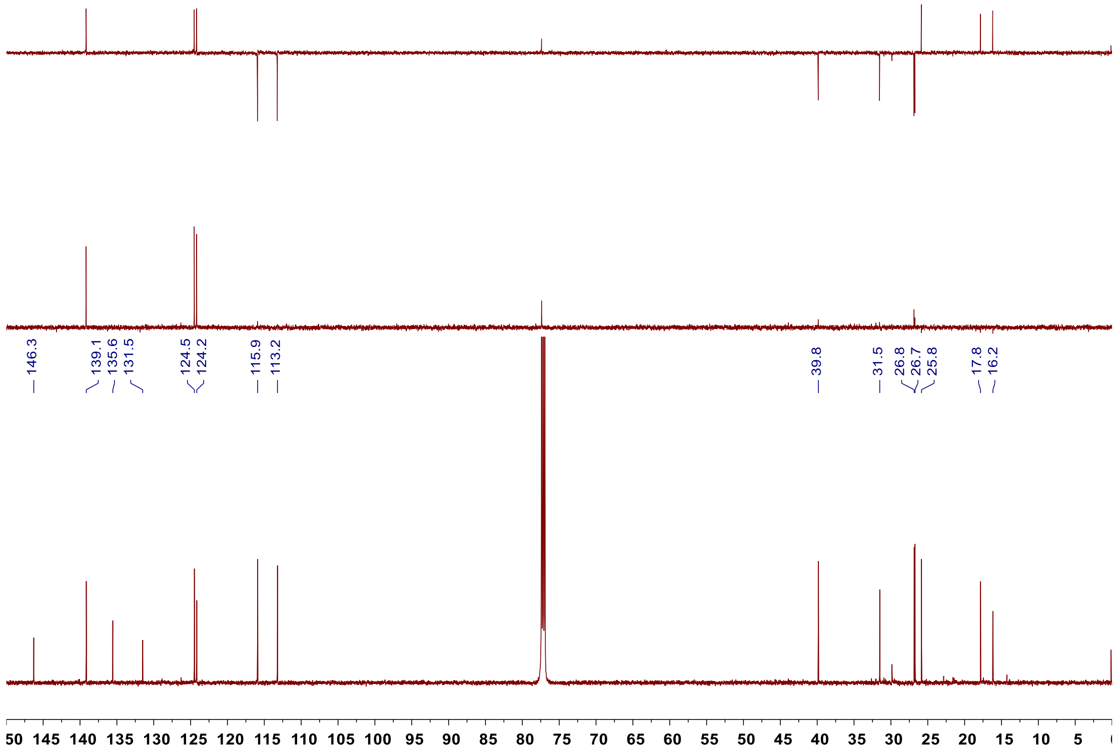

Figure S33. ^{13}C NMR spectrum of **6** in $CDCl_3$ at 600 MHz.

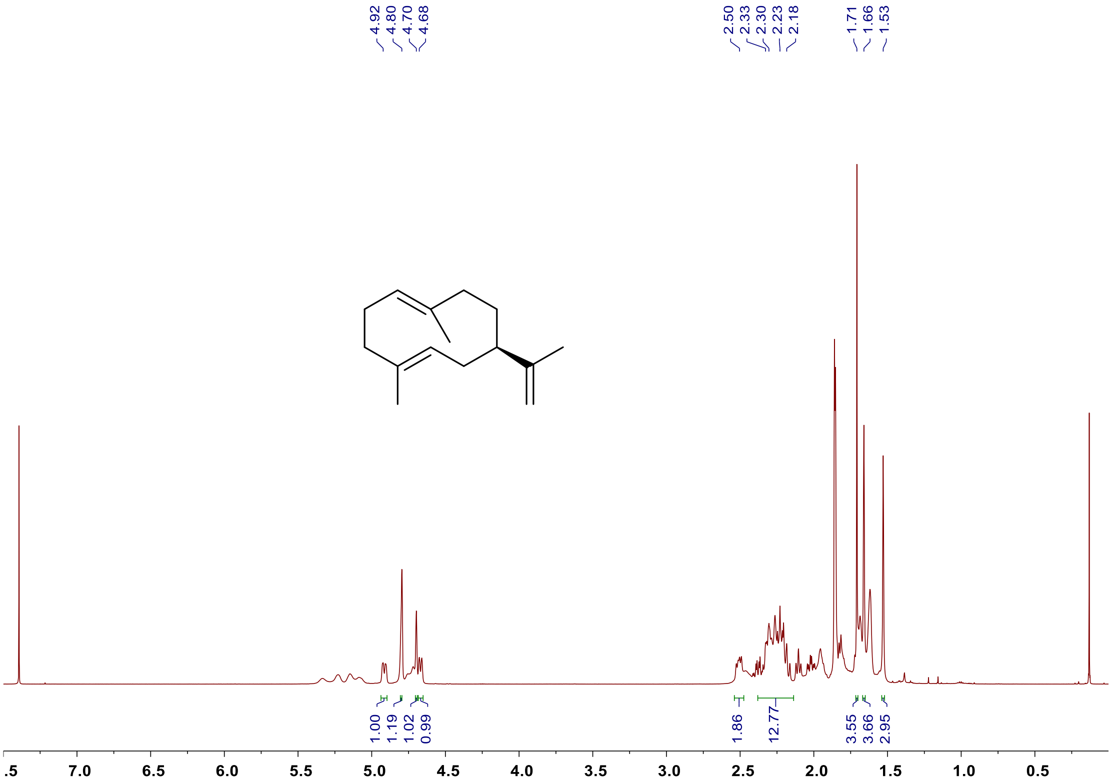

Figure S34. ^{1}H NMR spectrum of **7** in $CDCl_3$ at 600 MHz.

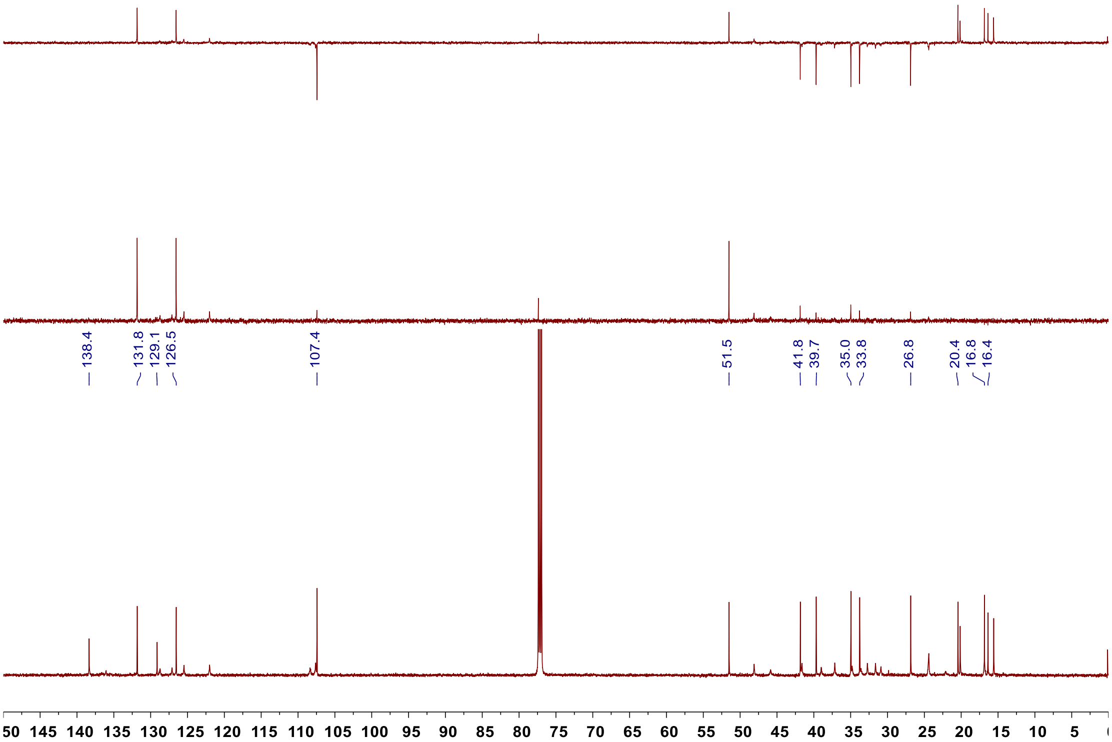

Figure S35. ^{13}C NMR spectrum of **7** in $CDCl_3$ at 600 MHz.

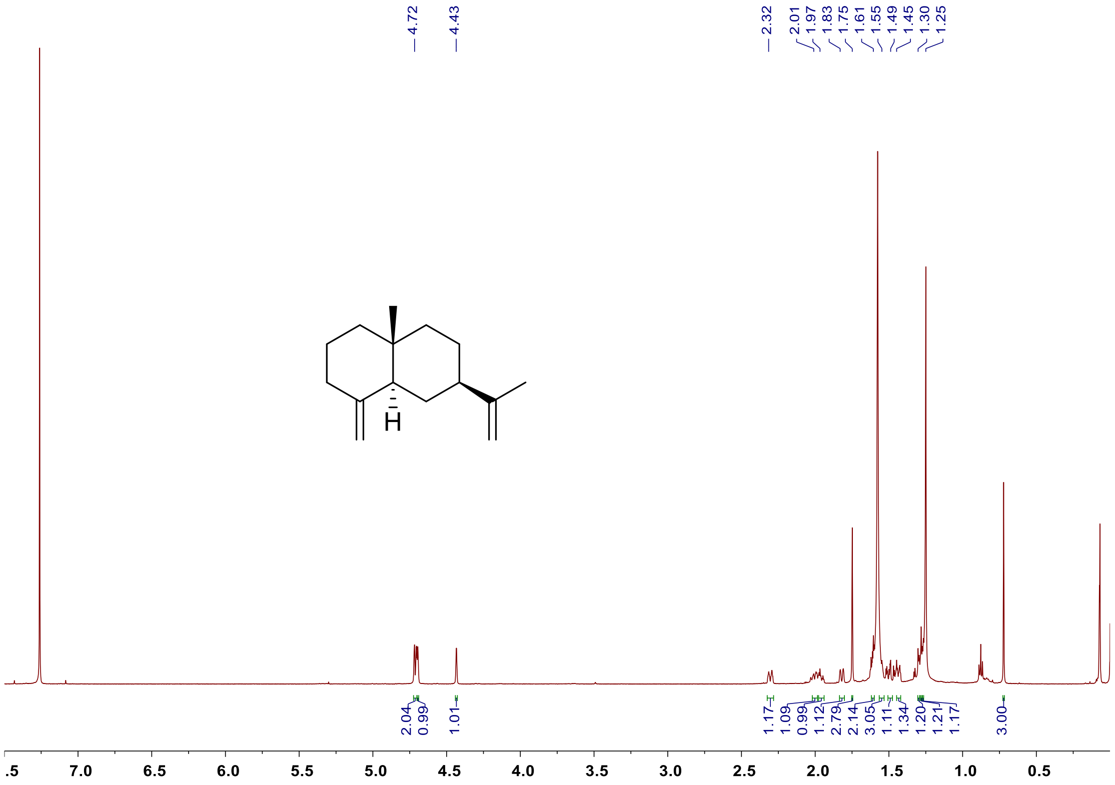

Figure S36. ^{1}H NMR spectrum of **7** 变 in $CDCl_3$ at 600 MHz.

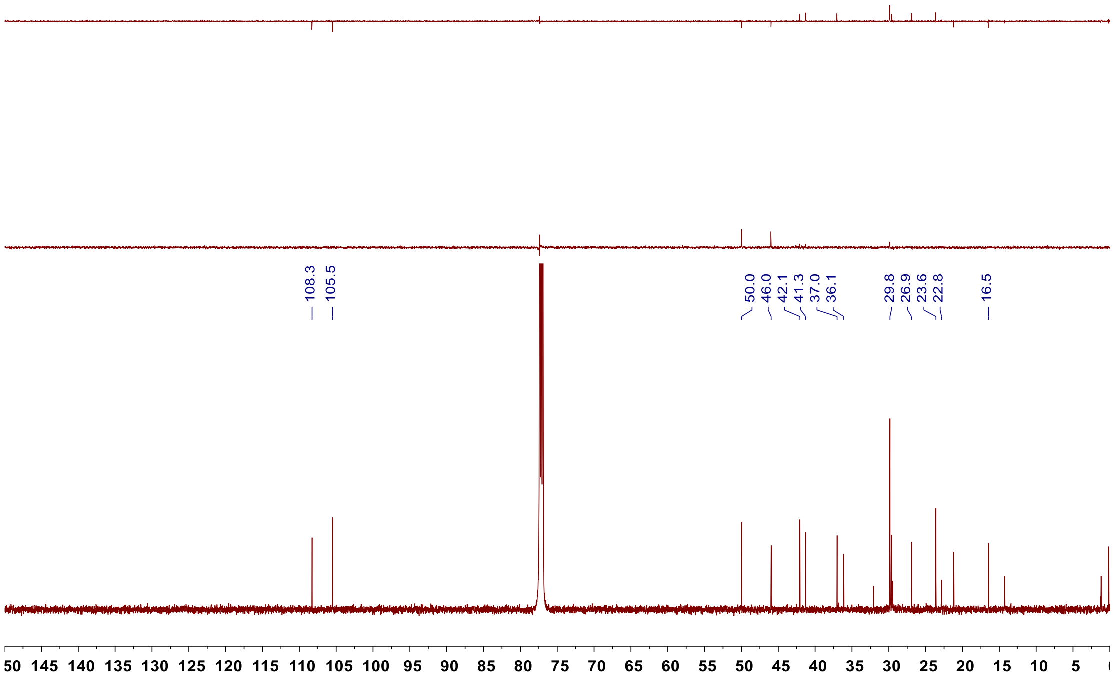

Figure S37. ^{13}C NMR spectrum of **7** 变 in $CDCl_3$ at 600 MHz.

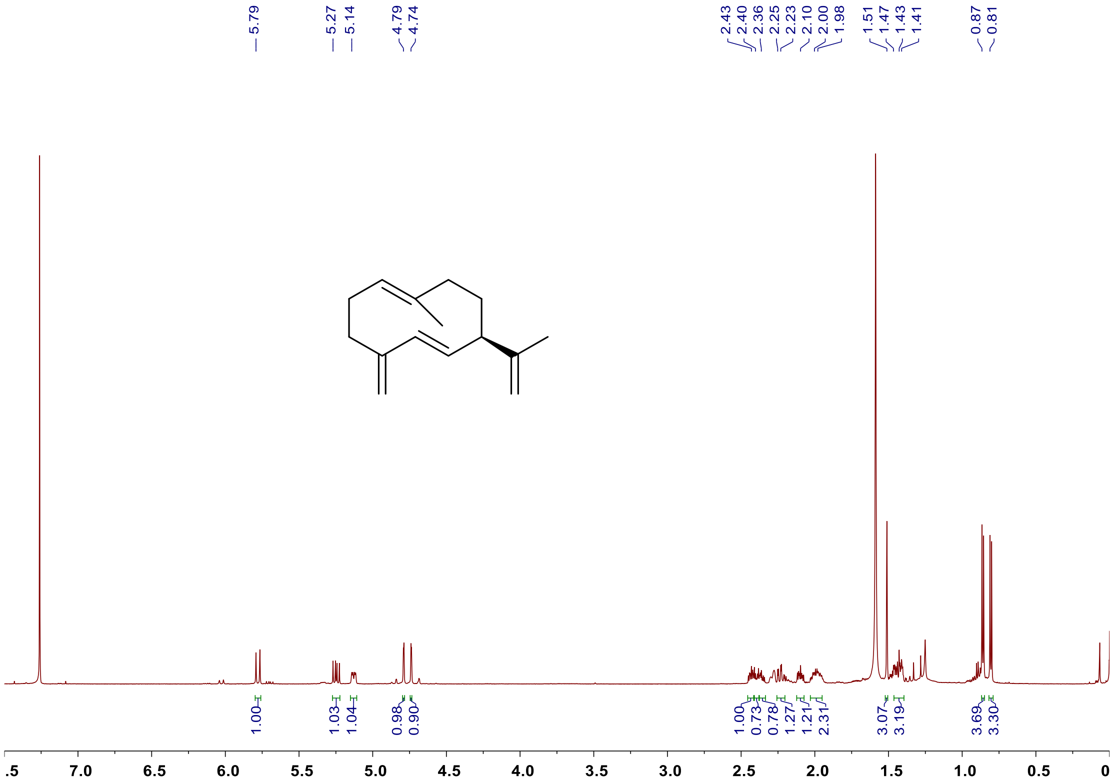

Figure S38. ¹H NMR spectrum of **8** in CDCl3 at 600 MHz.

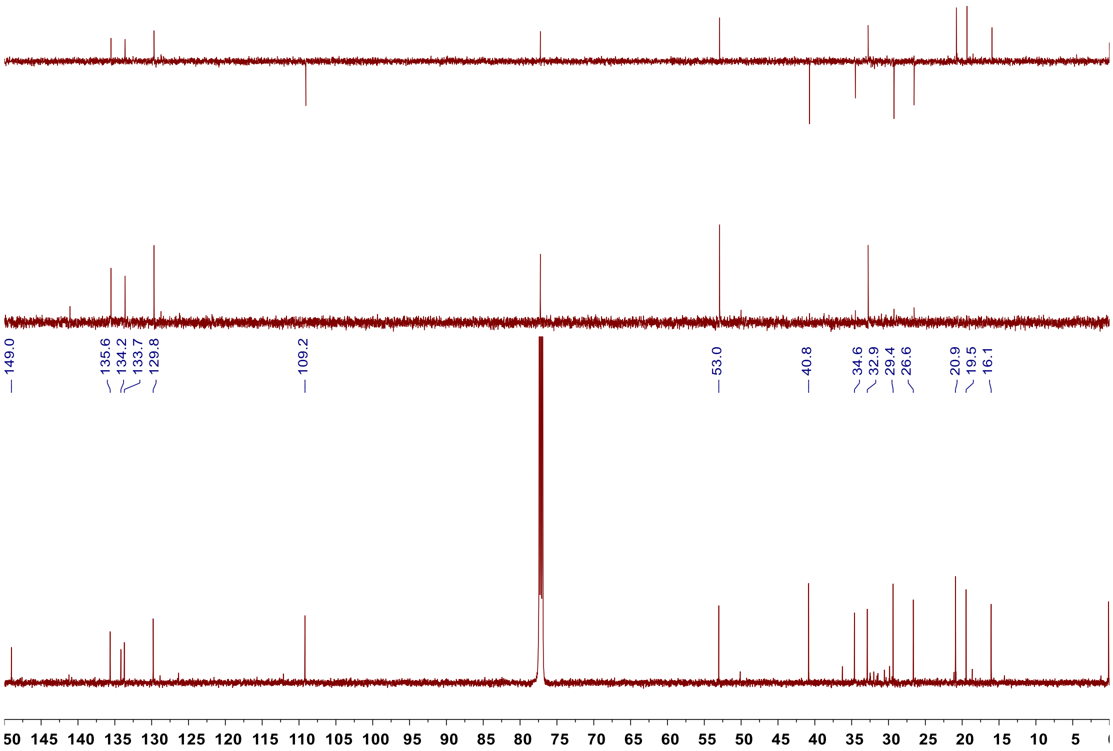

Figure S39. ¹³C NMR spectrum of **8** in CDCl3 at 600 MHz.

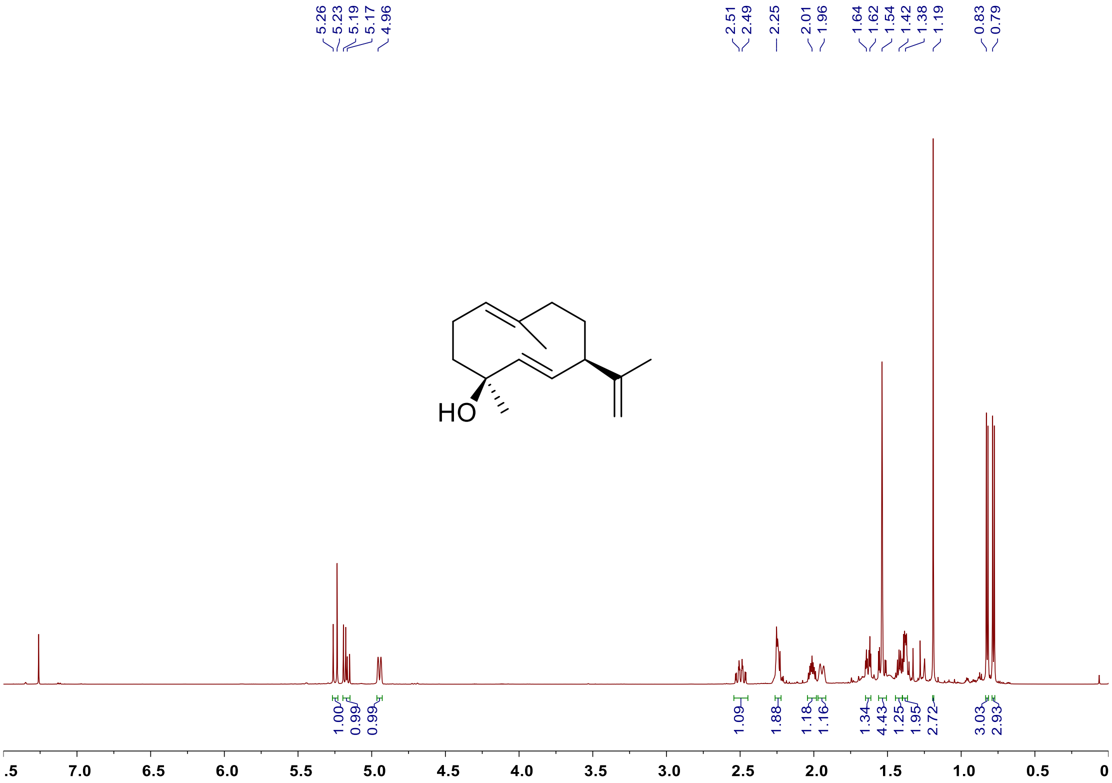

Figure S40. ^{1}H NMR spectrum of **9** in $CDCl_3$ at 600 MHz.

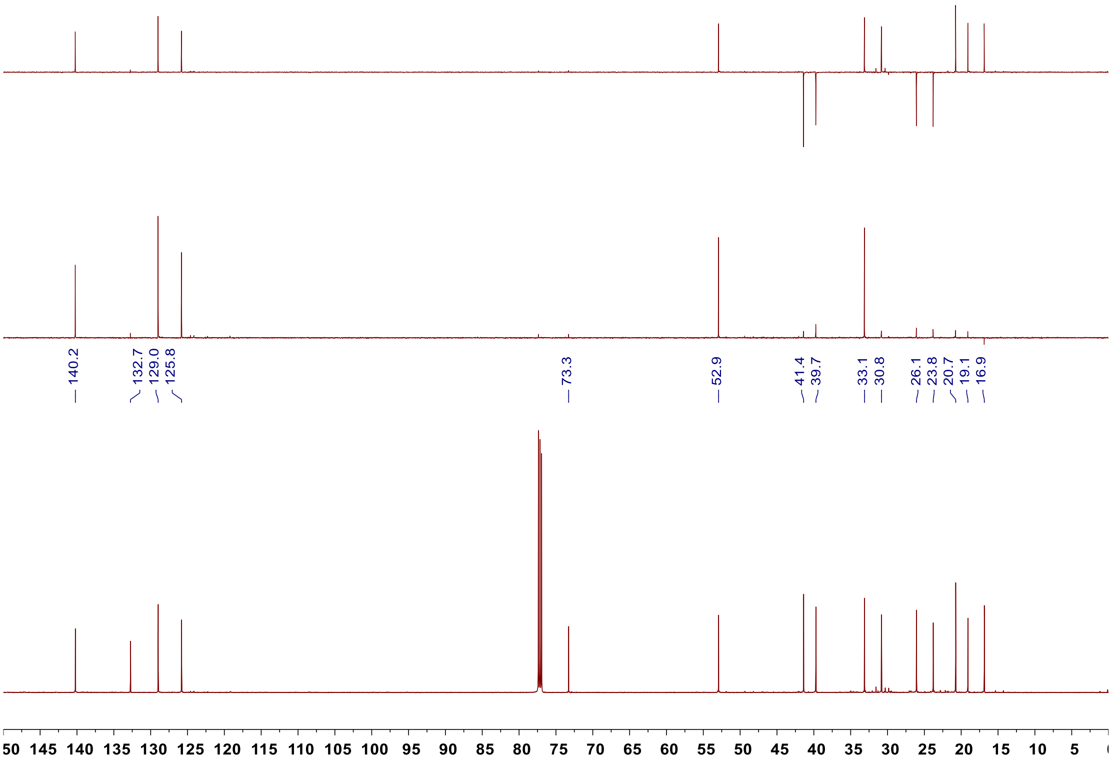

Figure S41. ^{13}C NMR spectrum of **9** in $CDCl_3$ at 600 MHz.

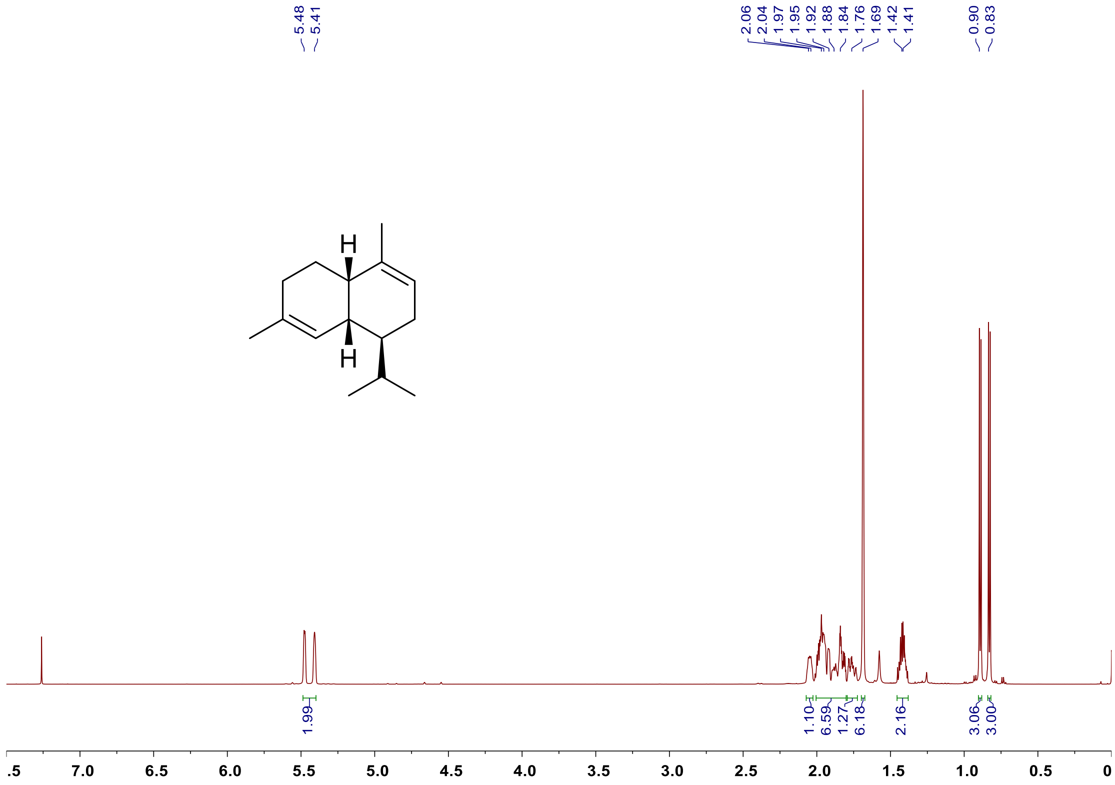

Figure S42. ^{1}H NMR spectrum of **10** in $CDCl_3$ at 600 MHz.

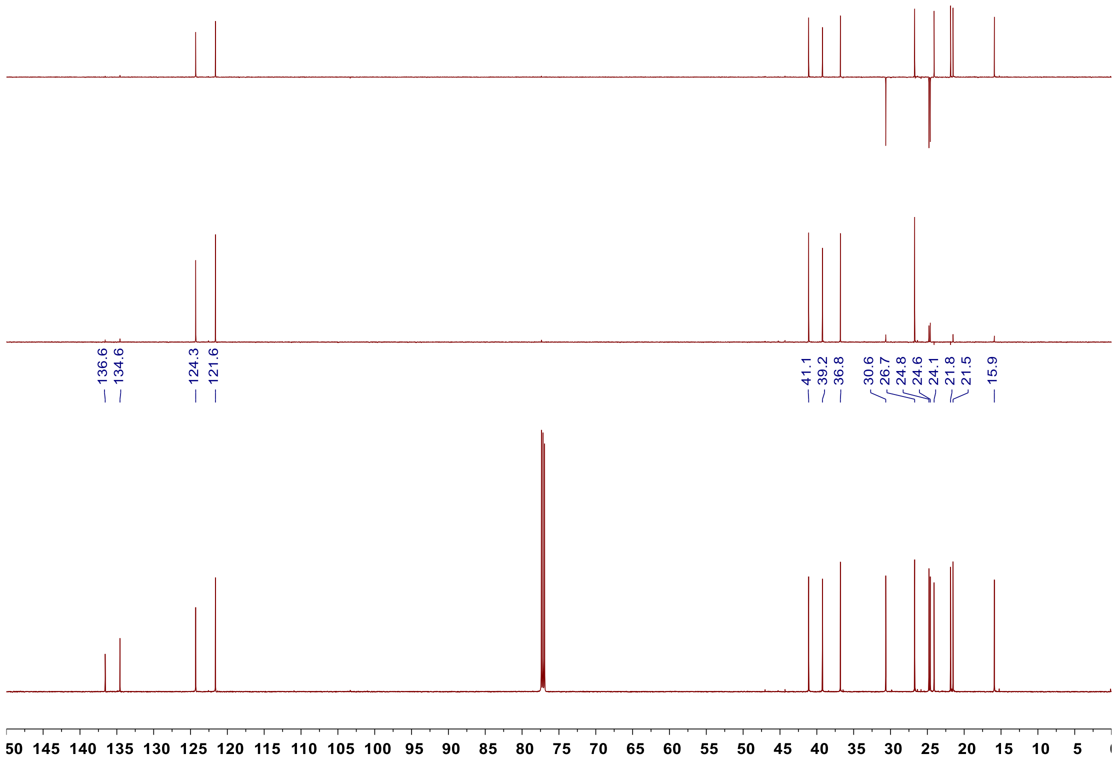

Figure S43. ^{13}C NMR spectrum of **10** in $CDCl_3$ at 600 MHz.

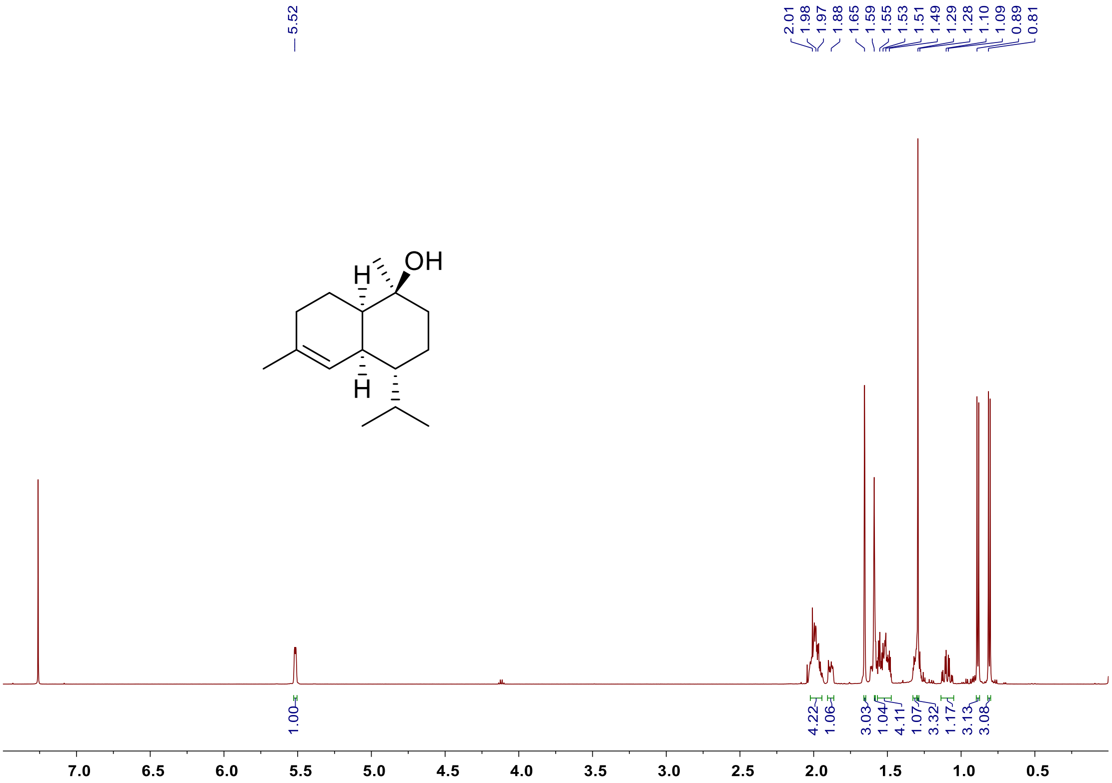

Figure S44. ^{13}C NMR spectrum of **11** in $CDCl_3$ at 600 MHz.

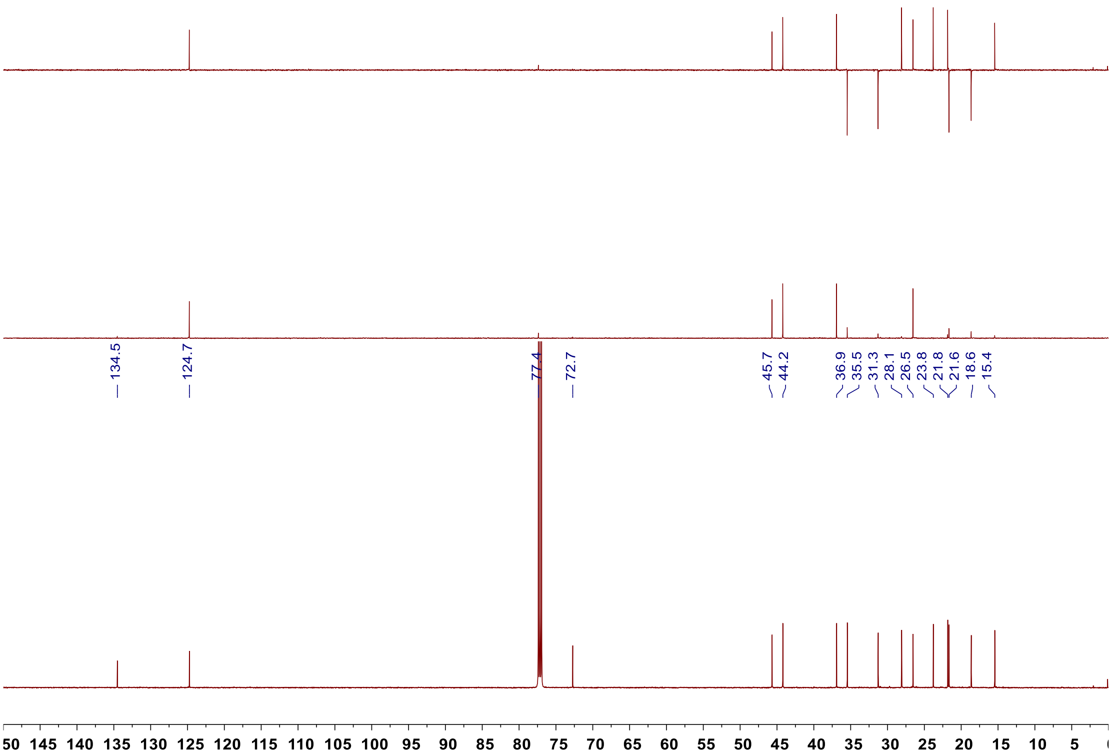

Figure S45. ^{13}C NMR spectrum of **11** in $CDCl_3$ at 600 MHz.

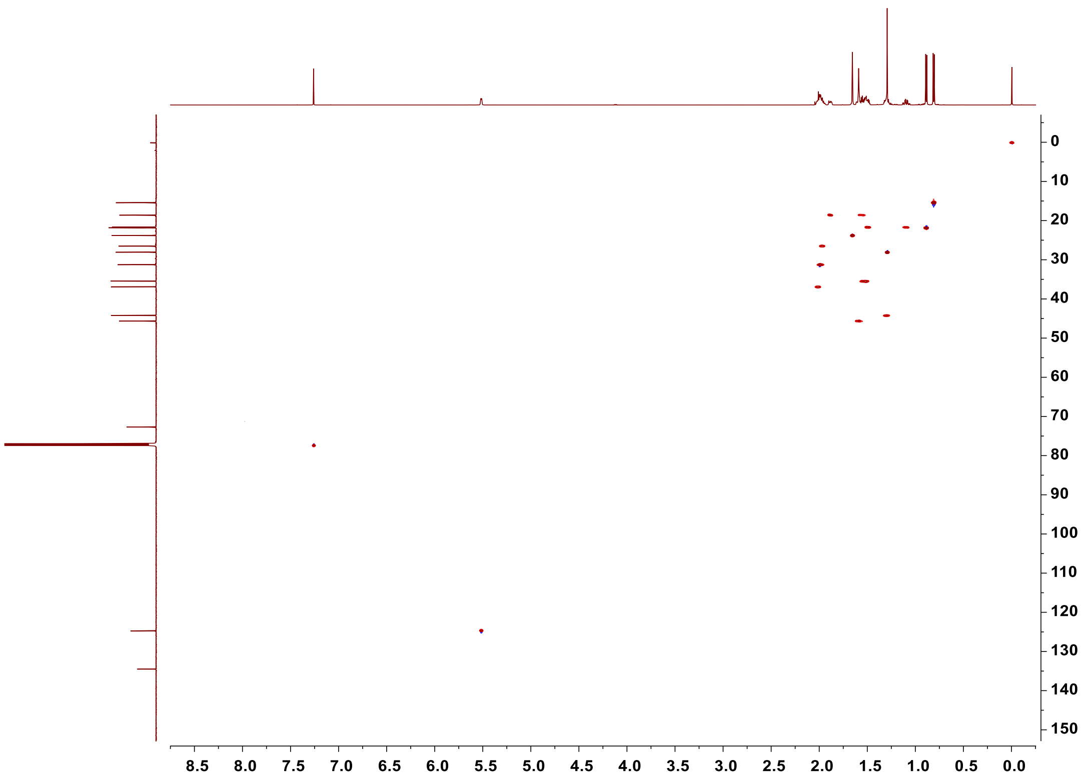

Figure S46. HSQC spectrum of **11** in $CDCl_3$ at 600 MHz.
